# Biomembrane‐coated nanosystems as next‐generation delivery systems for the treatment of gastrointestinal cancers

**DOI:** 10.1002/btm2.70006

**Published:** 2025-02-26

**Authors:** Joana Lopes, Daniela Lopes, Mahzad Motallebi, Mengguang Ye, Yuxiang Xue, Amélia C. F. Vieira, Sachin Kumar Singh, Kamal Dua, Francisco Veiga, Gautam Sethi, Ana Cláudia Paiva‐Santos, Pooyan Makvandi

**Affiliations:** ^1^ Department of Pharmaceutical Technology, Faculty of Pharmacy of the University of Coimbra University of Coimbra Coimbra Portugal; ^2^ LAQV/REQUIMTE, Department of Pharmaceutical Technology Faculty of Pharmacy of the University of Coimbra, University of Coimbra Coimbra Portugal; ^3^ Nanomedicine Research Association (NRA) Universal Scientific Education and Research Network (USERN) Tehran Iran; ^4^ Institute for Bioengineering, School of Engineering The University of Edinburgh Edinburgh UK; ^5^ School of Pharmaceutical Sciences Lovely Professional University Phagwara Punjab India; ^6^ Faculty of Health, Australian Research Centre in Complementary and Integrative Medicine University of Technology Sydney Sydney New South Wales Australia; ^7^ Discipline of Pharmacy, Graduate School of Health University of Technology Sydney Sydney New South Wales Australia; ^8^ Department of Pharmacology, Yong Loo Lin School of Medicine National University of Singapore Singapore; ^9^ The Quzhou Affiliated Hospital of Wenzhou Medical University Quzhou People's Hospital Quzhou China; ^10^ University Centre for Research & Development Chandigarh University Mohali India; ^11^ School of Chemistry Damghan University Damghan Iran; ^12^ Centre for Research Impact & Outcome, Chitkara College of Pharmacy Chitkara University Rajpura Punjab India

**Keywords:** biomimetic cell membrane‐coated nanosystems, cell membrane coatings, colorectal cancer, esophageal cancer, gastric cancer, gastrointestinal cancers, liver cancer, pancreatic cancer

## Abstract

Gastrointestinal cancers, a major global cause of cancer‐related mortality and disease burden, are a heterogeneous group of malignant aliments involving different organs of the digestive system. The late clinical diagnosis, genomic tumor heterogeneity, high complexity of the gastrointestinal tumor microenvironment, along with increasing treatment resistance have been recognized as the main contributing factors to the current inadequacy of the clinical interventions and poor prognosis of the gastrointestinal cancer patients. In the coming years, gastrointestinal cancer‐related global mortality is unfortunately predicted to increase due to the absence of early detection and effective therapeutic options. Biomembrane‐coated biomimetic nanoparticles (NPs) have recently been appointed as advanced nanotechnological tools for the clinical management of gastrointestinal cancers. These comprise not only cell‐mimicking nanodevices (the pioneers of this top‐down coating technology), but also exosome and bacterial mimetics. Due to their enhanced bio‐interfacing features, biocompatibility, immune evasion, and specific targetability to tumorous tissues, these biomimetic nanostructures have been successfully exploited to provide safer, effective, and targeted gastrointestinal cancer applications. This review highlights the latest research on biomembrane‐coated nanosystems for the clinical therapy and diagnosis of the most common and deadliest subtypes of gastrointestinal cancers, namely colorectal cancer, gastric cancer, liver cancer, esophageal cancer, and pancreatic cancer. The current challenges toward their clinical translation are also mentioned.

**Abbreviations**
5‐FU: 5‐fluorouracil
5‐FU ZGGO@SiO_2_
: ZGGO@SiO_2_ carrying anti‐cancer dug 5‐FU
5‐FU ZGGO@SiO_2_@LRM: 5‐FU ZGGO@SiO_2_ coated with *Lactobacillus reuteri* biofilms
AIE: aggregation‐induced emission
Apt‐RBC‐HMOS@DOX: MUC‐1 aptamer‐surface functionalized RBC‐HMOS@DOX
As: artesunate
BCL2: B‐cell lymphoma‐2
BF: BSA‐modified Fe_3_O_4_ NP
BFC: Ce6‐loaded BF
BiOI: bismuth oxyiodide
BiOI@M: BiOI nanorods camouflaged with M1 macrophage membrane (BiOI@M)
BLIPO‐I/D: LIPO‐I/D camouflaged by SW1990 pancreatic cancer cell membranes to yield a biomimetic core‐shell nanosystem
CaC: Cur‐loaded CaCO_3_ NP
CaCO_3_ NP: mesoporous calcium carbonate nanoparticle
CaCO_3_
: mesoporous calcium carbonate
CAR: chimeric antigen receptor
CDT: chemodynamic therapy
Ce6: chlorin e6
CMnMPt (also named CCM@Mn@MSN‐Pt(IV)): gastric cancer cell membrane‐coated MnMPt
CQ: chloroquine
CS‐6: gamabufotalin
CT: computed tomography
Cur: curcumin
Cy7: cyanine 7
DCPy: 2,6‐dichloropyridine
DLS: dynamic light scattering
DMEM: dulbecco's modified eagle medium
DOX: doxorubicin
*E. coli*
: *Escherichia coli*
EAC: esophageal adenocarcinoma
ECaC: EXO‐coated CaC
ECM: extracellular matrix
EGF: epidermal growth factor
EGFR: epidermal growth factor receptor
ELS: electrophoretic light scattering
EM: ESCC membrane
EPR: enhanced permeability and retention
ESCC: esophageal squamous cell carcinoma
EXO: CT26 colon cancer cell‐derived exosomal membrane
EYLN: pro‐inflammatory leukocyte membrane‐cloaked lipid nanovector
Fe_3_O_4_ NP: iron oxide nanoparticle
FLT: fluorescence imaging
FOLFIRINOX: 5‐fluorouracil, leucovorin, irinotecan, and oxaliplatin
GA: gambogic acid
GN: gelatin nanogel
GN/IRN: irinotecan‐loaded gelatin nanogel
GNR: gold nanorod
GOQDs: graphene oxide quantum dots
Gox: glucose oxidase
GPX4: glutathione peroxidase‐4
GSH: glutathione
HCC: hepatocellular carcinoma
hCTL: human cytotoxic CD8+ T lymphocyte
HMOS: hollow mesoporous organosilica nanoparticle
HMOS@DOX: DOX‐loaded HMOS
HPA: hydroxymethyl phenylboronic acid
ICD: immunogenic cell death
ICG: indocyanine green
ICG@MOF: ICG‐loaded MOF nanoparticle
ICG@RB‐MOF: red blood cell membrane‐coated ICG@MOF
IONP: iron‐oxide nanoparticle
Irgd: RGD internalizing peptide (a tumor‐penetrating peptide)
IRN: irinotecan
IV: intravenous
Jug: jOJuglone
JUNB: junB Proto‐oncogene
KRAS: Kirsten RAS
LIPO‐I/D: liposomes co‐loading chemotherapeutic drug doxorubicin and ICG (a photothermal agent for PTT and photosensitizer for PDT)
LPO: lipid peroxides
LPS: lipopolysaccharide
LRM: *Lactobacillus reuteri* (*L. reuteri*) biofilm
LTA: lipoteichoic acid
M@NPs/miR365 (also named M@NPs): NPs/miR365 coated with MC38 colon cancer cell membranes
Man‐EM: mannose‐functionalized red blood cell membrane
MBFC: CT26 colon cancer cell membrane‐coated BFC
MD: DCPy‐loaded MnO_2_ NP
miRNA: microRNA
MnM (also named Mn@MSN): Mn2 + −doped MSN
MnMPt (also named Mn@MSN/Pt(IV)): Pt(IV)‐loaded MnM MPt/Pt(IV)‐loaded MSN
MOF: metal–organic framework
MRI: magnetic resonance imaging
mRNAs: messenger RNAs
MSN: mesoporous silica nanoparticle
mTOR: mammalian target of rapamycin
MVP: major vault protein
NF‐ĸb: nuclear factor kappa‐light‐chain‐enhancer of activated B cells
NIR: near‐infrared
NP: nanoparticle
NPs/miR365 (also named NPs): PCPs loaded with miR365
nsPEF: nanosecond pulsed electric field
NTA: nanoparticle tracking analysis
OMVs: outer membrane vesicles
Oxa: oxaliplatin
PCL: poly(ε‐caprolactone)
PCL‐PEG: poly(ε‐caprolactone)‐poly(ethylene glycol)
PCPs: ε‐poly‐l‐lysine polypeptide‐functionalized poly (citrate‐peptide) nanoparticles
PDAC: pancreatic ductal adenocarcinoma
PDT: photodynamic therapy
PEG: polyethylene glycol
PEG‐PLGA: PEGylated‐poly (lactic‐*co*‐glycolic acid)
PEI: polyethylenimine
PET: positron emission computed tomography
PET‐CT: positron emission computed tomography‐computed tomography
PGMA: poly(glycidyl methacrylate)
PHBV: poly(3‐hydroxybutyrate‐*co*‐3‐hydroxyvalerate)
PIPD: PROTAC‐induced PDEẟ degrader
PLGA: poly (lactic‐*co*‐glycolic acid)
PLGA@GA: GA‐loaded PLGA nanosystem
PMD + L (EI): PMD plus external laser irradiation
PMD + L: PMD plus abdominal laser irradiation
PMD: platelet membrane‐coated MD
PMPNs: distearoyl phosphatidylethanolamine‐polyethylene glycol
PRNP: paclitaxel‐loaded PEG‐modified bovine serum albumin nanoparticle coated with a red blood cell membrane
PRNP‐Gel: injectable hydrogel
PROTAC: proteolysis‐targeting chimeric
PTM/GN/IRN: irinotecan‐loaded gelatin nanogel coated with platelets membranes
PTT: photothermal therapy
PTX: paclitaxel
PV: platelet membrane‐derived nanovesicle
R837: imiquimod a toll‐like receptor 7 (TLR 7) agonist
R837@PLGA: R837‐loaded PLGA
R837@PLGA@Neu: neutrophil membrane‐coated R837@PLGA
RBC: red blood cell
RBC‐HMOS@DOX: red blood cell membrane‐coated HMOS@DOX
RBCm: red blood cell membrane
RBCm‐(Jug, Oxa)‐iRGD‐NPs: iRGD‐modified red blood cell membrane‐coated nanoparticles co‐loaded with juglone and oxiplatin
RBCm‐(Jug, Oxa)‐NPs: red blood cell membrane‐coated nanoparticles co‐loaded with juglone and oxiplatin
RMD: red blood cell membrane‐coated MD
ROS: reactive oxygen species
RT: radiation therapy (or radiotherapy)
SDS‐PAGE: sodium dodecyl sulfate‐polyacrylamide gel electrophoresis
SDT: sono‐dynamic therapy
SEM: scanning electron microscopy
siIRAK4: siRNA targeting the IRAK4 gene
siLPCAT1: siRNA targeting the LPCAT1 gene
siRNA: small interfering RNA
SLC7A11: channel protein solute carrier family 7 member 11
TACE: transarterial chemoembolization
TAMs: tumor‐associated macrophages
TEM: transmission electron microscopy
TEP: TM‐EM‐coated PLGA
TGF‐β: transforming growth factor‐β
TGZ: zeolitic imidazolate framework‐8 NP co‐loaded with GOx and prodrug tirapazamine
TGZ@Em: red blood cell membrane‐coated TGZ
TiO_2_
: titanium dioxide nanoparticle
TLR 7: toll‐like receptor 7
TM: thylakoid membrane
TME: tumor microenvironment
TM‐EM: hybrid thylakoid‐ESCC membrane
TM‐EM@PLGA@GA (also named TEPG): TM‐EM‐coated PLGA@GA
TNF‐α: tumor necrosis factor‐α
TPG: TM‐coated PLGA@GA
TPGS: vitamin E polyethylene glycol succinate
TPZ: tirapazamine
US: ultrasound
VEGFR2: vascular endothelial growth factor receptor‐2
WBC: white blood cell
X‐PDT: x‐ray‐induced PDT
ZGGO@SiO_2_
: persistent luminescence mesoporous silica NPs
ZGGO@SiO_2_
: persistent luminescence mesoporous silica
ZIF‐8: zeolitic imidazolate framework‐8 (ZIF‐8)
ZIF‐90: zeolitic imidazolate framework‐90
ZTC: Ce6 and TPZ co‐loaded ZIF‐8 nanoparticle
ZTC: tirapazamine
ZTC@M: gastric cancer cell membrane‐coated ZTC
β‐CD: β‐cyclodextrin

**Translational Impact Statement**

This paper reviews biomembrane‐coated biomimetic nanoparticles for managing gastrointestinal cancers, a major cause of global cancer mortality. These nanostructures, including cell, exosome, and bacterial mimetics, offer enhanced biocompatibility, immune evasion, and tumor targeting. The review examines their applications in therapy and diagnosis of colorectal, gastric, liver, esophageal, and pancreatic cancers.

## 1 INTRODUCTION

Cancer is a life‐threatening public problem accounting for millions of deaths worldwide.^1^ The conventional modalities available for cancer therapy include surgery, radiotherapy, and chemotherapy, with the last being the most common treatment option for eradicating cancer.^1^, ^2^, ^3^ Regrettably, chemotherapy remains unsatisfactory because of its serious undesirable side effects, which are due to the inability of chemotherapeutic drugs to selectively target tumor tissues, leading to off‐target toxicity in healthy tissues.^3^, ^4^ To address the reduced tumor‐targeting specificity of anticancer therapies and to mitigate off‐target toxicity, nanoparticles (NPs) have been introduced in the biomedical field, either as a carrier for therapeutic molecules and imaging agents to specific target sites, or as a pharmacologically active component due to their intrinsic physicochemical features.^5^

Recently, a pioneering biomimetic nanotechnology based on coating NPs with biological membranes has been applied in the field of cancer therapy, diagnosis, and theranostics.^6^, ^7^, ^8^ This approach was designed to produce cell‐mimicking nanosystems able to combine the physiochemical features and biopharmaceutical benefits of nanomaterials, with the intrinsic biofunctions of biomembranes, while overcoming the limitations of NPs.^1^, ^9^, ^10^ Despite the pronounced advantages in therapeutic efficacy and safety, NP‐based medicines are rapidly eliminated from systemic circulation and suffer from poor biocompatibility,^6^, ^10^, ^11^ which represent critical obstacles to their successful clinical implementation.^11^ So far, numerous membrane coatings have been studied in this nature‐inspired‐coating technology, including single or hybrid cell membranes,^12^, ^13^, ^14^ cell‐secreted extracellular vesicle membranes (e.g., exosomes),^7^, ^15^, ^16^ and bacterial membranes. By means of membrane coating strategies, NPs can be provided with improved biocompatibility, immune evasion, prolonged blood circulation, and cell‐targeting abilities, contributing to enhance the in vivo performance, therapeutic efficacy, alongside the pharmacokinetic and safety profiles of NPs.^1^, ^6^, ^9^, ^10^

Gastrointestinal cancer refers to a high‐fatal malignant condition affecting different organs located in the digestive system, including among others, the esophagus, stomach, liver, pancreas, gallbladder, colon, rectum, and anus.^17^, ^18^, ^19^ They rank as one of the most serious health problems worldwide on account of high incidence, late diagnosis, recurrence rate, and cancer‐related mortality.^20^, ^21^ The most common types of gastrointestinal cancers include pancreatic, liver, gastric, esophageal, and colorectal cancer.^22^ According to epidemiological data from the Global Cancer Observatory (2020), among the five most common types of gastrointestinal cancer, colorectal cancer is associated with the highest incidence (10.2%) and mortality (9.2%) rates, accounting for the highest number of cancer‐related deaths. It is followed by gastric cancer, liver cancer, esophageal cancer, and finally pancreatic cancer in both incidence and cancer‐related mortality.^20^ Overall, gastrointestinal cancers account for 26% of all cancers globally and represent nearly 35% of all cancer‐related mortality.^23^

Various biomembranes and NP types have been investigated for gastrointestinal cancer therapy. In the present review, the most recent and original research regarding the use of biomembrane‐coated NPs for clinical gastrointestinal cancer therapy is summarized, including colorectal cancer, gastric cancer, liver cancer, esophageal cancer, and pancreatic cancer. To this end, the readers will be first introduced to the contemporary therapeutic and diagnostic approaches for gastrointestinal cancers as well as their main limitations, followed by the preparation process and characterization of biomembrane‐surface engineered NPs. Finally, an overview of the studies employing biomembrane‐coated NPs for the treatment of gastrointestinal cancers and the future prospectives will be highlighted.

## 2 THE DIGESTIVE SYSTEM: ANATOMY, PHYSIOLOGY, AND FUNCTIONS

The digestive system is composed of several organs involved in the digestion, a complex process required to extract the nutrients essential for the body's vital functions from the ingested food.^24^ The digestive system is responsible for: (1) digestion of ingested food components; (2) absorption of nutrients; and (3) elimination of solid wastes.^17^ The main functions of the digestive system are to break down the ingested food into basic units, absorb the food‐derived nutrients, and discharge the remaining waste products.^17^, ^24^

The human digestive system is composed of the gastrointestinal tract (a.k.a., digestive tract) and the accessory organs.^24^ The gastrointestinal tract is a muscular tube of approximately 8–9 m in length all the way from the mouth to the anus, and it is typically divided into two parts: the upper and lower tracts.^17^, ^24^ The upper gastrointestinal tract includes the oral cavity, pharynx, esophagus, stomach, and duodenum (the first part of the small intestine).^17^, ^25^ The lower gastrointestinal tract includes the jejunum and ileum (the other two parts of the small intestine), and the large intestine (cecum, colon, rectum, and anus).^17^, ^25^ Each organ of the digestive tract performs specific activities in the digestive process and waste excretion. The accessory digestive organs include the teeth, tongue, salivary glands, liver, gallbladder, and pancreas, which play key roles in the digestive functions.^24^

Gastrointestinal cancers can be located in all organs belonging to the digestive system, ranging from the esophagus to the rectum. The accessory organs in the digestive system, including the liver, gallbladder, and pancreas, can also be affected.^17^

## 3 GASTROINTESTINAL CANCER MANAGEMENT STRATEGIES

### 3.1 Gastrointestinal cancer types: Biological and clinical features

Gastrointestinal cancers are among the most prevalent, life‐threatening, and potentially fatal malignancies.^18^, ^19^ They account for the highest incidence burden, cancer‐related mortality, and morbidity worldwide.^20^, ^22^, ^26^ In general, cancers affecting the digestive system have the worst prognosis and the lowest 5‐year survival rates.^17^

At present, numerous biological and clinical features of gastrointestinal malignancies are complicating early‐stage diagnosis and decreasing the therapeutic effectiveness.^27^, ^28^, ^29^ First, the absence of clinical biomarkers to detect the tumor‐initiating stages is a major contributing factor to the high mortality, metastatic rates, and tumor aggressiveness.^28^, ^29^ In addition, gastrointestinal cancers represent a highly heterogeneous group of malignancies. This heterogeneity can be either inter‐patient (different clinical manifestations and therapeutic efficacy among individual patients), or intra‐tumor (distinct subsets of cancer cells in the same tumor with varying therapeutic susceptibility).^27^, ^29^ The genomic heterogeneity, high tumor cell mutation rates, treatment resistance, and recurrence rates characteristics of gastrointestinal tumors are often caused by hypoxia, a common feature of solid tumors.^28^ Hypoxic conditions within the tumor microenvironment are closely related to tumor progression, metastasis, immunosuppression, and genetic/epigenetic mutations on tumor cells via reactive oxygen species (ROS)‐induced DNA damage.^28^ The existence of different molecular subtypes of each gastrointestinal cancer has a significant impact not only on cancer diagnosis, progression, and recurrence rates, but also on the effectiveness of treatments and prognosis.^27^, ^29^, ^30^ As individual patients respond differently to treatments, based on the tumor‐specific genetic profile, the success of various therapeutic approaches has been compromised.^27^, ^28^

### 3.2 Challenges in the diagnosis and treatment of gastrointestinal cancers

Gastrointestinal malignancies have unique properties that make them difficult to manage. These hurdles arise from their hard‐to‐reach locations, making the therapeutic regimes less specific and precise.^18^ This may compromise therapeutic efficacy and negatively influence the prognosis of patients with gastrointestinal cancers.

**FIGURE 1:**
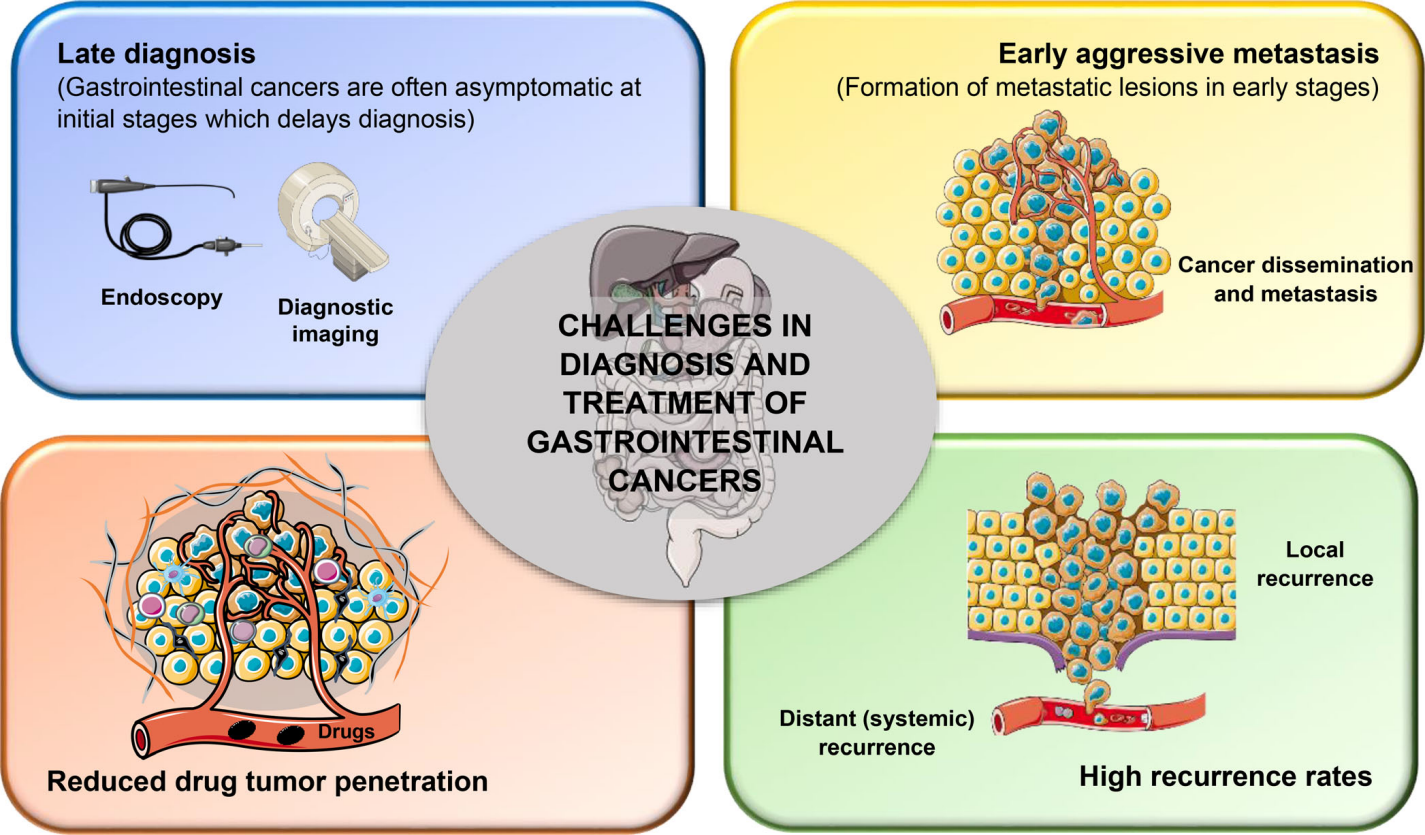
The main challenges in the diagnosis and treatment of gastrointestinal cancers. These include: (1) late diagnosis that often occurs only at advanced‐stage cancers (since gastrointestinal cancers are often asymptomatic at initial stages), (2) aggressive dissemination and metastasis in early‐stage cancers, (3) reduced drug penetration in tumors which compromises the therapeutic effectiveness of cancer therapy, and (4) high local and distant recurrence rates.

Contemporary approaches for managing gastrointestinal cancers continue to face several problematic and challenging issues.^18^ These include: (1) late diagnosis, since gastrointestinal cancer patients are often asymptomatic in early stages (do not show any relevant symptoms in initial stages), which may delay the clinical diagnosis that only occurs at advanced stages of the disease; (2) aggressive cancer metastasis in the early stages of the disease; and (3) reduced drug penetration into tumorous tissues due to presence of connective tissue (fibrotic tumor stroma) that constitutes an important barrier, protecting cancer cells from therapeutic molecules. Usually, the gastrointestinal tumor microenvironment is highly complex, containing a diversified array of immunosuppressive cells (e.g., M2 macrophages), extracellular matrix components (e.g., fibronectin), and a poorly penetrable tumor stroma that impedes accurate tumor‐targeted drug delivery.^31^ All of this contributes to reduce therapeutic effectiveness. Another issue of concern is the (4) high local recurrence and distant metastasis rates, even after surgical resection of solid tumors (Figure 1).^18^, ^20^, ^21^, ^32^ On account of these challenges, gastrointestinal cancers are recognized as one of the major leading causes of cancer‐related morbidity and mortality worldwide.^18^, ^20^

### 3.3 Conventional diagnostic imaging and treatment approaches

The early‐stage‐gastrointestinal cancers are often asymptomatic and can only be detected by endoscopy or biopsy.^17^ Most neoplastic diseases of the digestive system are already in advanced stages when diagnosed and respond poorly to conventional therapeutic regimes.^20^, ^21^ The 5‐year survival rate of patients with advanced‐stage‐gastrointestinal cancers is normally under 30%.^17^, ^22^ These overwhelming numbers highlight the urgent need for early diagnosis and innovative therapies that can eradicate cancer, suppress the dissemination of cancer cells and metastasis, as well as improve patient survival rate.

Cancer bioimaging is crucial for early and accurate cancer diagnosis, monitoring of tumor progression, detection of metastasis, and tumor staging.^33^, ^34^ Imaging of tumorous tissues prior to treatment can guarantee an effective and localized cancer therapy.^23^, ^33^, ^34^ For the diagnosis of tumors located in the digestive system, various imaging techniques have been proposed in clinical practice. Endoscopy (the most specific and sensitive), ultrasound imaging, magnetic resonance imaging (MRI), computed tomography (CT), positron emission computed tomography (PET), and positron emission tomography‐computed tomography (PET‐CT) are among those techniques.^17^, ^23^ Although some progress has been made recently in this field, these diagnostic approaches employ contrast agents with serious side effects, restricted tumor‐specific accumulation, and short half‐life.^23^

In the therapeutic setting, conventional approaches include surgery, radiotherapy, and chemotherapy.^20^, ^32^, ^35^ Depending on the location, size, metastatic stage, and cancer type, a multimodal approach combining different therapies may be required. Unfortunately, these therapies are largely unsatisfactory in terms of both therapeutic efficacy and safety goals.^32^

Surgery (surgical removal of the local solid tumors) is the most common therapeutic approach for the earliest stage of gastrointestinal cancers.^20^ It can be used with a curative purpose. Before or after the surgical procedure, it may be necessary to combine radiotherapy and/or chemotherapy to downsize the tumor, facilitate the elimination of non‐removed tumor cells that may otherwise induce tumor recurrence, and eradicate the cancer. However, the accurate identification of tumor margins and removal of malignant lesions without damaging adjacent healthy cells are challenging issues related to this treatment option.^35^, ^36^

Radiation therapy (a.k.a., radiotherapy) employs radiation to destroy cancer cells. Radiation can be used alone with a curative purpose, as neoadjuvant therapy before surgery to reduce tumor size, as adjuvant therapy after the surgical procedure to eradicate remaining cancer cells, or as palliate therapy with the single aim of enhancing quality of life and reduce pain‐related symptoms. Its main limitations are the possible damage to surrounding non‐diseased tissues, the emergence of radiotherapy‐resistant tumor cells, and poor tumor cell killing efficiency in poorly oxygenated tissues.^35^, ^36^

Chemotherapy consists of the systemic administration of chemotherapeutic drugs to destroy fast‐replicating tumor cells. It can also be used with the goal of a curative, adjuvant, neoadjuvant, or palliative therapy.^36^ The poor tumor‐targeting specificity, increasing resistance of tumor cells to chemotherapy, and severe adverse effects of chemotherapeutic drugs are the main chemotherapy‐related concerns.^36^ Chemotherapy is often associated with high risk of off‐target toxicity, due to the non‐specific distribution at non‐target cells.^18^, ^35^

Accordingly, there is an emerging need for improved diagnostic and therapeutic approaches that can profoundly improve both cancer diagnosis in the early stages of the disease and the therapeutic efficacy for a better prognosis of patients suffering with gastrointestinal cancers.^20^, ^21^, ^26^ To do so, much attention has been devoted to nanotechnology. The emergence and application of nanotechnological tools in medical settings not only offered a solution to some of the drawbacks mentioned above, but also enabled the development of multimodal approaches that combine different diagnostic techniques and therapies.

### 3.4 Nanotechnological approaches in gastrointestinal cancers: An overview

NP‐based formulations have achieved great potential in the field of cancer therapy and diagnosis. Nanosized materials can allow a more targeted and precise therapy by enabling a controlled and site‐specific release of therapeutic molecules and reducing their systemic toxicity.^32^ There are several advantages in using nanomedicines that are mainly attributed to their unique biopharmaceutical features. These include their (1) nanoscale size (ranging from 1 nm to 100 nm) and higher surface area beneficial for improved in vivo biointeractions; (2) ability to protect encapsulated drugs from biodegradation and premature leakage; (3) sustained and controlled release of loaded drugs, which reduces frequency of administration; (4) improved biodistribution at targeted tumor tissues by the enhanced permeability and retention (EPR) effect; (5) enhanced pharmacokinetic and safety profiles; (6) higher loading and encapsulation efficiency (enabling the co‐delivery of two or more compounds); and (7) greater flexibility to undergo surface modifications for active targeted drug delivery.^32^, ^35^, ^37^

So far, different nanotechnological approaches have been designed to improve the diagnosis and treatment of gastrointestinal cancers (Table 1).^17^ However, although promising, nanotechnology‐based drug delivery systems still encounter numerous challenges that may decrease their systemic circulation time and the delivery efficiency to the target site. These include: (1) their inability to cross biological barriers; (2) their low biocompatibility; and (3) their accelerated clearance by the mononuclear phagocyte system. Besides, the active cell‐targeting features of NPs often depend on the surface incorporation of targeting ligands (such as small peptides, proteins, or antibodies), capable of interacting with specific receptors highly expressed at target diseased cells. These bottom‐up approaches are laborious and complex.^38^ Taken together, these findings suggest the great need for new technological strategies to overcome these shortcomings of NPs.

**TABLE 1:** Overview of different nanotechnology‐based formulations for diagnosis and therapy of gastrointestinal cancers.

Gastrointestinal cancer	NP carrier	Encapsulated drug(s)	Main results	Refs.
Colorectal cancer	PLGA NP	Quercetin and caffeic‐acid phenethyl ester (CAPE)	Improved physicochemical properties (hydrophobicity) and release profile of both anticancer compounds Superior antitumor efficacy	39
	EGF‐functionalized PLGA NP	5‐Fluorouracil and perfluorocarbon	Selective uptake by cancer cells Efficient suppression of tumor growth in vivo	40
	PEGylated PLGA NP	5‐Fluorouracil and chrysin	Efficient co‐delivery of both compounds to HT29 colon cancer cells Synergistic anticancer effects for superior tumor eradication	41
	PHBV/PLGA NP	5‐Fluorouracil and oxaliplatin	Pronounced antitumor efficiency compared to free drugs Improved the cytotoxic effects of both chemotherapeutic drugs Activation of apoptotic pathways and tumor cell death Less chemotherapy‐related side effects	42
Gastric cancer	PEGylated manganese‐containing polydopamine NP	—	Superior tumor accumulation via the EPR effect Effective NIR‐induced PTT Synergistic antitumor effects of PTT and chemodynamic therapy Tumor imaging via MRI	43
	ZIF‐90 NP	Plumbagin (natural anticancer compound)	Increased circulation half‐time of plumbagin Superior accumulation of plumbagin at tumor sites Efficient suppression of gastric cancer growth via inhibition of the YAP1 signaling pathway	44
	Mesoporous silica NP	Resveratrol (a natural antitumor polyphenol)	Improved oral bioavailability and pharmacokinetic profile (rapid clearance) of resveratrol Enhanced accumulation of resveratrol at targeted tumorous tissues Superior antitumor efficacy	45
	PLGA NP functionalized with an antibody targeting sialyl‐Lewis A (sLeA)	5‐fluorouracil and paclitaxel	Active tumor‐targeting features conferred by the NP surface modification Superior uptake by sLeA‐expressing gastric cancer cells Successful delivery of both drugs to metastatic gastric cancer cells	46
	TiO_2_ NP	5‐fluorouracil	Increased ROS production and inhibition of autophagic flux Improved cytotoxic and apoptotic effects of 5‐fluorouracil	47
Liver cancer	Calcium phosphate NP modified A54 peptide (a liver cancer cell targeting peptide)	Gd‐DTPA (MRI contrast agent)	Higher tumor accumulation provided by the A54 tumor‐targeting peptide Superior MRI performance compared to free Gd‐DTPA Potential as an MRI contrast agent for early diagnosis of liver cancer	48
	Mesoporous silica NP	Indocyanine green (photothermal agent) and sorafenib	Effective theranostic nanoplatform for liver cancer diagnosis and therapy Effective NIR‐induced PTT Tumor‐targeted fluorescence imaging Reduced side effects of sorafenib	49
	PGMA polymeric NP functionalized with SP94 (a tumor‐targeting peptide)	Sorafenib	Intrinsic tumor‐targeting features via passive and active mechanisms Selective uptake by hepatocellular carcinoma cells via active targeting Notorious antitumor effects compared to conventional chemotherapy Reduced side effects of chemotherapy	50
Esophageal cancer	Carboxymethyl chitosan NP dual‐functionalized with histidine cholesteryl ester and EGFR antibodies	Doxorubicin and siRNAs targeting the *MVP* and *BCL2* genes	Efficient co‐delivery of doxorubicin and siRNAs to targeted tumor sites Silencing of multidrug resistance‐related genes (*MVP* and *BCL2*) Enhanced antitumor efficacy of chemotherapy in multidrug resistance esophageal cancer	51
	Copper‐cysteamine NP	Disulfiram	Tumor specific‐accumulation via the EPR effect Synergistic anticancer effects against esophageal cancer Increased ROS production Enhanced tumor cell apoptosis by blocking the NT‐κβ (p65) translocation to the nucleus	52
Pancreatic cancer	TAB004 antibody‐decorated mesoporous silica NP	Gemcitabine and cisplatin	Specific tumor accumulation provided by the TAB004 antibody Tumor targeted co‐delivery of chemotherapeutics Reduced off‐target toxicity of chemotherapy Enhanced antitumor outcomes	53
	Redox‐sensitive polymeric micelles functionalized with GE11 (a EGFR targeting peptide)	Gemcitabine and miR‐519c	Selective uptake by EGFR‐expressing pancreatic cancer cells via active targeting Reduced hypoxia‐induced gemcitabine resistance in miR‐519c under‐expressed pancreatic cancer Superior tumor eradication with chemotherapy, with minimal toxicity	54
	Mesoporous silica NP	Palbociclib (a CDK4/6 inhibitor) and hydroxychloroquine (an autophagy inhibitor)	Improved drug distribution at targeted pancreatic tumorous tissues Apoptotic tumor cell death induced by synchronous inhibition of CDK4/6 and autophagy pathways	55

Abbreviations: BCL2, B‐cell lymphoma‐2; EGF, epidermal growth factor; EGFR, epidermal growth factor receptor; EPR, enhanced permeability and retention; MRI, magnetic resonance imaging; MVP, major vault protein; NIR, near‐infrared; NP, nanoparticle; PEG, polyethylene glycol; PGMA, poly(glycidyl methacrylate); PHBV, poly(3‐hydroxybutyrate‐*co*‐3‐hydroxyvalerate); PLGA, poly(lactic‐*co*‐glycolic acid); PTT, photothermal therapy; ROS, reactive oxygen species; SiRNA, small interfering RNA; TiO_2_, titanium dioxide nanoparticle; ZIF‐90, zeolitic imidazolate framework‐90.

## 4 ENGINEERING OF BIOMEMBRANE‐COATED BIOMIMETIC NANOPARTICLES: PREPARATION AND CHARACTERIZATION

Cell membrane‐coated NPs are composed of an NP core surrounded by a layer of a natural cell membrane.^10^, ^56^ These biomimetic nanosystems have been widely studied in nanomedicine and have been recognized as advanced cell‐mimicking drug delivery nanosystems for several biomedical applications, on account of their enhanced bio‐interfacing features.^1^, ^9^ This novel approach harnesses the intrinsic features of biomembranes to confer specific cell‐like biofunctions to NPs, such as superior biocompatibility, immune evasion, prolonged systemic circulation capabilities, as well as specific tissue‐homing features.^7^ These can ultimately improve their pharmacokinetic and safety profiles.^9^, ^10^, ^11^

The concept of using naturally derived cell membranes to coat NPs first appeared in 2011, using red blood cells as the membrane suppliers to coat poly(lactic‐*co*‐glycolic acid) (PLGA) NPs.^11^ Since the first reported cell membrane‐coated NPs, this biomimetic strategy has been applied to other cell membrane sources, including platelets, white blood cells, stem cells, cancer cells, and others, in which each cell membrane type provides specific features related to their parent cells.^10^, ^56^, ^57^ Thus, according to the selected cell membrane source, cell membrane‐coated NPs are expected to possess specific cell biofunctions, which are directly related to the surface repertoire of donor cells. Multifunctional hybrid cell membranes that combine the biofunctionalities of distinct cells have also been studied (Table 2).^4^, ^5^, ^58^

**TABLE 2:** Common cell membranes for nanoparticle coatings and their key donor cell‐related features and limitations.

Source cell membrane	Specific donor cell‐related features	Pros	Cos	Refs.
Red blood cell	Long systemic circulation (≈120 days) Immune evasion	Easy manipulation Simple membrane extraction procedure Biocompatibility	Restricted tumor‐targeting	65, 66, 67, 68
Platelet	CTC‐targeting features Selective targeting to inflamed/injured vasculature	Refined tumor and inflammation targeting Biocompatibility	Reduced concentration in bloodstream	65, 68, 69, 70
White blood cell (granulocytes and agranulocytes)	Immune evasion Prolonged systemic circulation Intrinsic targeting features to inflammatory and tumor sites via chemotactic recruitment	Intrinsic immunomodulatory effects Biocompatibility	High heterogeneity Complexity of extraction Immunogenicity	12, 65, 66, 68, 71
Stem cell	Tumor‐targeting features Damaged tissue repair Immune evasion	Refined tumor and inflammation targeting Biocompatibility	Elevated costs	65, 66, 68, 72
Cancer cell	Homotypic tumor‐homing features Immune evasion Deep tumor penetration	Antitumor vaccination Biocompatibility Convenient in vitro manipulation	Carcinogenic risk	65, 66, 68, 73
Hybrid cell (combination of single‐cell membranes)	Integrates multiple biofunctions derived from each cell membrane type	Multifunctional and multi‐action for improved targeting and performance	High heterogeneity and low production	68, 74, 75

Abbreviation: CTC, circulating tumor cell.

Compared with uncoated NPs, cell membrane‐coated NPs were proved with better biomimicry and immune evasion. By using natural cell membranes, cell membrane‐coated NPs can “cloak” themselves, reducing recognition by the immune system and allowing for longer circulation times. This enables them to selectively accumulate in areas of the body where they can be most effective, such as tumor sites or areas of inflammation. Besides, the membranes used to coat these NPs often retain surface markers, such as proteins and antigens, which can improve their targeting ability. Besides, the coating of biomembranes provides NPs with an enhanced penetration capability, which can enhance their ability to penetrate biological barriers. On one hand, the biomembrane on the surface of NPs can enhance cellular uptake through mechanisms like receptor‐mediated endocytosis. The presence of membrane proteins on the NP surface can facilitate the internalization of NPs by target cells, improving drug delivery and therapeutic efficacy. However, due to the heterogeneity of cell membranes, the composition of these membranes can vary due to differences in the cells used for their production. This inconsistency can reduce the selectivity of the NPs, affecting their behavior in vivo.^59^

Although naturally derived cell membranes were the pioneers in this top‐down coating strategy, this concept has also been extended to other biomembrane sources, such as the membranes of cell‐secreted extracellular vesicles (exosomes), bacteria (including both Gram‐positive and Gram‐negative) as well as bacterial outer membrane vesicles (OMVs) (Table 3).

**TABLE 3:** Other biomembrane sources for nanoparticle coatings and their key features and limitations.

Source biomembrane	Specific features	Pros	Cons	Refs.
Cell‐secreted extracellular vesicles (exosomes)	Immune evasion Prolonged systemic circulation Tumor‐homing features (to homologous parent tumor cells) Deep tumor penetration	Properties inherited from parent cells (cells of origin) Ability to cross biological barriers (e.g., BBB) Nanoscale size Biocompatibility	Low production and isolation yields High heterogeneity Complex intra‐exosomal content	76
Bacteria and OMVs	Immunomodulation Immune response activation Antibacterial and photothermal activity	Easy manufacturing Large‐scale and inexpensive production	Presence of immunogenic agents (LPS and LTA) Toxic nature Unknown construction routes and cargo packaging mechanisms Separation and purification challenges for clinical applications	64

Abbreviations: BBB, Blood–brain barrier; LPS, lipopolysaccharide; LTA, lipoteichoic acid; OMV, outer membrane vesicle.

Exosome‐extracted membranes have attracted attention for coating biomimetic NPs due to the fact that their proteins which are still active following nanocarrier synthesis, are responsible for their immune evasion ability, direct tumor‐targeting, longer residence in bloodstream, plus determining their high level of cellular uptake.^60^, ^61^, ^62^

Bacterial membranes are other novel sources that have represented potential efficacy for camouflaging the NPs in various biomedical applications including cancer and bacterial infections treatment due to their innate large‐scale production ability in inexpensive culture conditions and immunomodulatory profiles.^63^ Additionally, OMVs, generally produced via bacteria's (Gram‐negative bacteria) outer membrane blebbing, have also shown to be promising cell sources for this purpose resulting from their biological functions including photothermal activity for bacterial infections therapy and immune response activation.^63^, ^64^

### 4.1 Preparation of cell membrane‐coated nanoparticles

**FIGURE 2:**
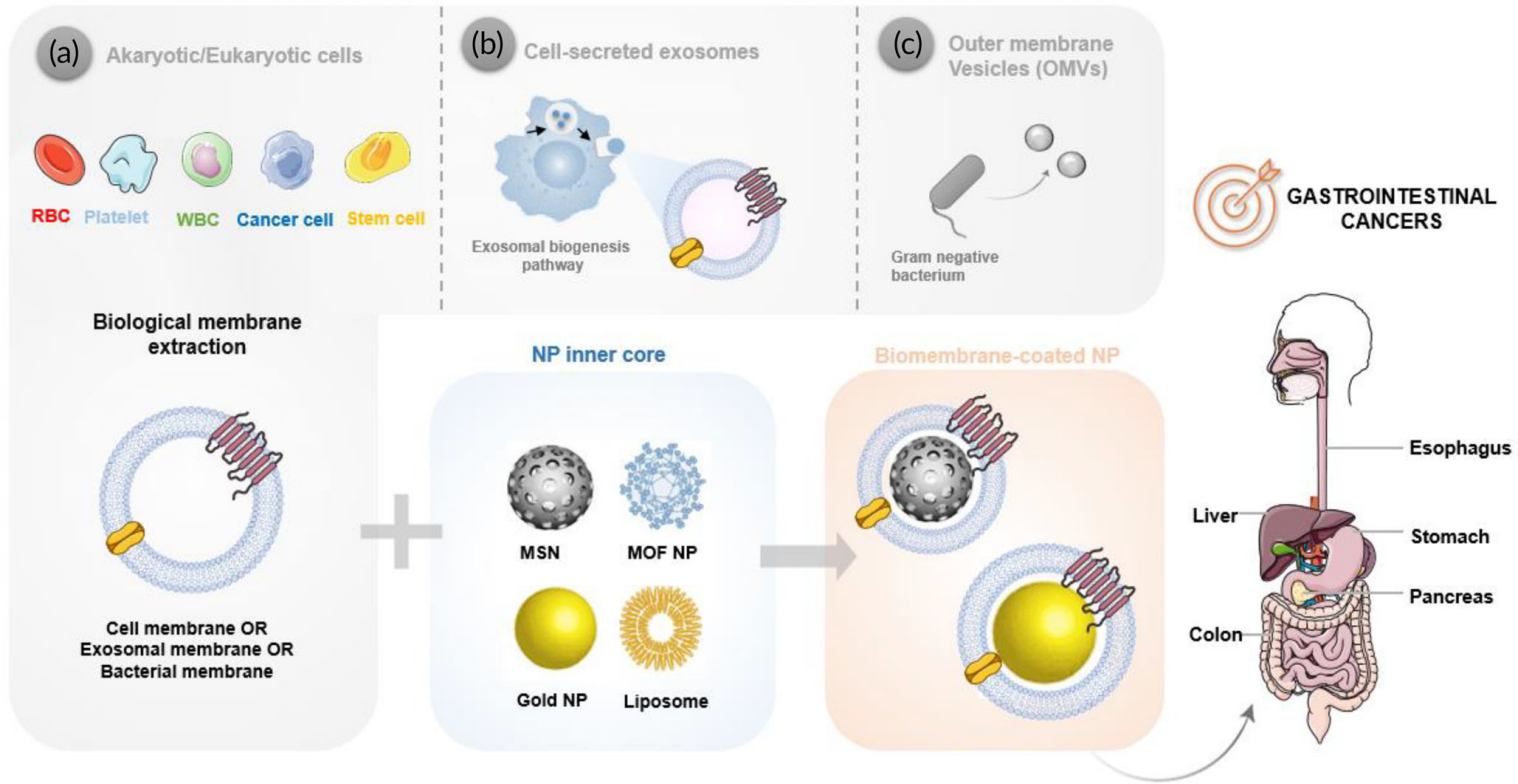
Schematic illustration of the preparation process of (a) cell membrane‐coated NPs, (b) exosomal membrane‐coated NPs, and (c) bacterial OMV‐coated NPs. The first step includes the extraction of biological membranes from the sources of interest. For cell membrane‐coated NPs, the preparation of the cell membrane‐derived nanovesicles by extrusion or sonication is required. After the selection and preparation of the NP inner core (e.g., MSNs, MOF NPs, gold NPs, or liposomes), biomembranes can be coated onto the NP core by various methods, such as co‐extrusion or sonication. Then, the resulting biomembrane‐coated nanosystems can be used for several biomedical applications, namely for the treatment of various gastrointestinal cancers, including colorectal cancer, gastric cancer, esophageal cancer, liver cancer, and pancreatic cancer. MOF, metal–organic framework; MSN, mesoporous silica nanoparticle; NP, nanoparticle; OMV, outer membrane vesicle; RBC, red blood cell; WBC, white blood cell.

Cell membrane‐coated NPs are synthesized by a nature‐inspired top‐down technology, which generally comprises three steps: (1) cell membrane extraction and preparation of cell membrane‐derived nanovesicles; (2) preparation of the NP inner core; and (3) fusion of the NP core with the cell membrane‐derived nanovesicles through coating techniques. Figure 2a shows the three‐step preparation of cell membrane‐surface engineered NPs.

#### 4.1.1 Cell membrane extraction and preparation of cell membrane nanovesicles

The protocols for cell membrane extraction are determined by the cell type of interest, however, the most common approaches include a combination of cell disruption methods, which are designed to disrupt the membrane structure and induce cell lysis.^77^ Generally, the process of cell membrane extraction involves hypotonic lysis, followed by homogenization, centrifugation, and membrane purification, to remove intracellular components while leaving surface membrane proteins intact.^38^ Hence, the protocols for cell membrane extraction should be as gentle as possible to faithfully preserve the surface markers and the biological features of natural cell membranes.^77^ Subsequently, the emptied cells can be extruded through porous membranes or sonicated to prepare the cell membrane nanovesicles.^4^, ^5^

#### 4.1.2 Nanoparticle inner core preparation

Over the years, the use of these biomimetic nanosystems has been expanded to different types of cell membrane‐derived membranes and several types of NPs. These include both organic NP cores (e.g., PLGA and poly(ε‐caprolactone) [PCL]), and inorganic NP cores (e.g., metal–organic framework [MOF], mesoporous silica NPs [MSNs], upconversion NPs, gold NPs, iron‐oxide NPs [IONPs], and gold‐iron oxide NPs).^4^, ^73^ These nanomaterials can be used not only as effective drug delivery systems to carry therapeutic and imaging agents to target sites, but can also be employed to accomplish specific biological functions owing to their intrinsic physicochemical features.^5^ For instance, upconversion NPs have been employed for imaging applications, due to their intrinsic ability to convert near‐infrared (NIR) light into visible light. Gold NPs have been exploited for photothermal therapy (PTT), owing to their ability to convert the NIR light into cytotoxic heat, and IONPs have been investigated for both imaging applications and PTT, since the iron oxide can act as a contrast agent for MRI, while the gold NP can act as a photothermal agent for PTT.^5^, ^78^ Thus, according to the intended application, a proper selection of the NP core must be made.^5^

#### 4.1.3 Coating the nanoparticle core with the cell membrane‐derived nanovesicles

The process of camouflaging NP cores with the cell membrane nanovesicles can be achieved by different coating methods, such as (1) physical extrusion (or co‐extrusion), (2) sonication, (3) electroporation, or (4) microfluidic electroporation.^57^, ^79^, ^80^ A comparative analysis of the different coating methods is presented in Table 4. In physical extrusion (co‐extrusion), NP cores and cell membrane nanovesicles are combined and repeatedly co‐extruded through porous membranes using extruders, while sonication employs ultrasonic disruptive energy forces, leading to the spontaneous formation of a core‐shell nanostructure, with the advantage of losing less material compared to physical extrusion.^58^, ^78^, ^79^, ^80^ On the other hand, electroporation uses electrical pulses to open transient pores in the plasma membranes through which NPs can enter.^78^, ^80^ More recently, a microfluidic electroporation‐based technique was developed for membrane coating of NP cores.^4^, ^58^, ^80^, ^81^ Although alternative methods for NP coating have been proposed over time, physical extrusion and sonication remain the most frequently used techniques for membrane coating onto NPs.^79^

**TABLE 4:** Comparative analysis of the different coating methods for assembling cell membrane‐coated NPs.

Coating approach	Description of the approach	Superiority	Deficiency	Refs.
Physical extrusion (or co‐extrusion)	Extracted membranes and NPs are co‐extruded through porous membranes (extruders) The mechanical force induced by extrusion causes the disruption of the membrane structure, allowing its reassembly around the NP core	Simple and practical operational steps Effective production One of the most commonly used coating techniques	Possible disruption of the membrane integrity Time‐consuming and labor‐intensive ↓ scalability (difficult large‐scale production) ↑ materials loss (due to the material deposition onto extruder devices)	68
Sonication (ultrasound)	Extracted membranes and NPs are mixed and sonicated. The mixture is exposed to ultrasound waves at a determined frequency (ranging from 20 to 50 kHz) for a determined time The ultrasonic energy induces the spontaneous assembly of the membrane around the NP core	↓ materials loss (compared to physical extrusion) ↑ scalability (more efficient for large‐scale production) One of the most commonly used coating techniques	Possible disruption of the membrane integrity Time‐consuming and labor‐intensive Possible affecting the size and stability of NPs Non‐uniform membrane coating	68
Electroporation	Extracted membranes and NPs are mixed and exposed to electrical pulses (external electric fields) for a certain period of time The electrical pulses increase the permeability of the membranes, opening transient micropores through which NPs can diffuse	↓ time‐consuming and labor‐intensive (compared to co‐extrusion and sonication) ↓ possibility of affecting the properties of the NP cores	↑ complexity (complex operational steps)	68
Microfluidic electroporation	Combines electroporation with the use of microfluidic devices Extracted membranes and NPs are mixed in a microfluidic device By passing through the electroporation zone of the microfluidic device, transient pores on the membrane structure are formed (by exposure to electrical pulses), allowing NPs entry	↓ time‐consuming and labor‐intensive (compared to co‐extrusion and sonication) ↓ possibility of destroying NPs (obtain uniform sizes) Continuous and one‐step production ↑ reproducibility ↑ scalability (more efficient for large‐scale production)	↑ costs Complex procedure	68

Abbreviations: NP, nanoparticle; ↑, enhancement; ↓, reduction.

### 4.2 Preparation of other biomembrane‐coated nanoparticles

Similar to cell membrane‐mimicking NPs, the fabrication of exosomal membrane‐coated NPs also requires (1) exosome isolation and membrane extraction; (2) preparation of the NP inner core; and (3) fusion of the NP core with the exosomal membrane^15^, ^16^ (Figure 2b).

Firstly, cell‐secreted exosomes must be isolated by ultracentrifugation from the cell supernatant, followed by hypotonic treatment to remove the intra‐exosomal content and extract the emptied exosomal membranes. The next step involves the selection and preparation of the NP inner core, which according to the intended application can have either organic or inorganic natures. Finally, the extracted exosomal membrane must be camouflaged onto NP cores to yield exosomal membrane‐coated NPs. So far, different strategies have been reported including physical extrusion through porous membranes, sonication, direct incubation of NPs with living cells, direct incubation of NPs with isolated cell‐secreted exosomes, electroporation, as well as microfluidic sonication‐based techniques.^15^, ^16^

Additionally, the preparation of bacterial membrane‐ and bacterial OMV‐coated NPs has also been investigated (Figure 2c). At first, low‐speed centrifugation for separating the bacteria from the culture medium, followed by filtration and crude bacterial membrane vesicles concentration is applied. The final step of separation consists of ultracentrifugation at decreased temperatures which could be followed by multiple filtration and centrifugation steps to separate the bacterial membrane vesicles. For the purification, sucrose density gradients centrifugation and size exclusion chromatography techniques are used. Following preparation of the desired NP inner core, versatile techniques including electroporation, sonication, co‐extrusion, simple incubation, incubation with parent bacteria, and parent bacteria transformation are applied in order to NP coating and surface modification.^63^

In the context of OMVs‐camouflaged NPs fabrication, there is still no single standardized strategy but conventionally, ultracentrifugation and precipitation are applied to extract OMVs from Gram‐negative bacteria. Herein, centrifugation removes the bacteria cells and contaminants and the collected supernatant will be pelleted by ultracentrifugation. In order to separate the OMVs through the precipitation method, using saturated salt in the supernatant comprised solution, leads to protein solution's stability disruption, protein aggregation, and OMVs precipitation. Similar to other membranes, sonication and co‐extrusion are the techniques by which different NPs are coated by OMVs, after NPs' inner core preparation.^64^

### 4.3 Characterization of biomembrane‐coated nanoparticles

The characterization of the final nanosystem, in terms of its physicochemical and biological properties, is a critical step to confirm that the NP inner core has been successfully coated with the biomembrane and ensure the correct formation of the core‐shell nanostructure.^10^, ^73^, ^77^

#### 4.3.1 Physicochemical characterization

Typically, the characterization of the final nanosystem includes a complete analysis of its physicochemical properties, including the surface morphology (observed by microscopic analysis techniques), the hydrodynamic diameter, and the zeta potential. It is proved that the positively charged NPs are capable of disturbing the cell membrane arrangement, leading to the formation of particle aggregates, whereas a successful cell membrane coating would contribute to increased biocompatibility in negatively charged NPs resulting in an ideal “right‐side‐out” orientation. Collectively, these analyses provide useful and valuable insights over the effectiveness of the membrane coating.^59^, ^68^

For surface morphology analysis and visualization of the core‐shell NPs, techniques such as transmission electron microscopy (TEM) or scanning electron microscopy (SEM) are frequently used. Another imaging technique commonly employed for this purpose is confocal laser scanning microscopy (CLSM).^59^, ^68^ Additionally, particle size and zeta potential measurements are also required to ensure successful membrane coating. Particle size is evaluated by dynamic light scattering (DLS) or NP tracking analysis (NTA), and zeta potential (surface charge) by electrophoretic light scattering (ELS). It is worth mentioning that the membrane coating is generally thin, and so, a slightly increase in particle size and thickness of ~10–20 nm is expected when comparing the pre‐ and post‐membrane coating (Table 5).^59^

**TABLE 5:** Common parameters and respective techniques used to evaluate the physiochemical and biological properties of biomembrane‐coated nanoparticles (NPs).

Type of characterization	Parameter	Technique(s)	Description of the technique(s)	Expected result(s)	Refs.
Physicochemical characterization	Surface morphology (microscopic techniques)	TEM (2D) SEM (3D) CLSM	TEM: In the TEM images, the NP core is evident observed as a dense region, and the membrane shell is the thin layer surrounding it. For better resolution and visualization, negative stains (e.g., uranyl acetate) are used CLSM: The NP core and the membrane are labeled with different fluorescent dyes (in the pre‐coating phase), to analyze the extent and integrity of the membrane coating	TEM: Observation of a core‐shell nanostructure with an increase in thickness ranging from ~10 nm to 20 nm (regarding the shell coating) CLSM: Observation of a uniform and successful membrane coating surrounding the NP core	10, 59, 77, 82, 83, 84
	Hydrodynamic diameter	DLS NTA	DLS: The hydrodynamic diameter and PDI are measured based on the Brownian motion in liquid medium. The speed of particles is directly related to their size (smaller particles move quicker) NTA: Another technique used to measure the particle size based on the light scattering ability and Brownian motion in liquid medium	Increase of ~10 nm to 20 nm in particle size compared to non‐coated NPs (due to the membrane coating)	10, 59, 77
	Zeta potential	ELS	ELS: The zeta potential (surface charge) is measured based on the electrophoretic mobility of particles in suspension	Negative zeta potential measurement with a similar charge to that of the extracted membrane	59, 85
Biological characterization (membrane protein study)	Surface protein profile	SDS‐PAGE Western blotting	SDS‐PAGE: Comparison of protein profiles in source cell membranes, extracted membranes, and coated NPs. Briefly, a polyacrylamide gel matrix, a detergent (e.g. SDS) and a reducing agent are used to denature the proteins and impart a negative charge to them. The protein mixture is then applied to the gel, and an electric field allows the proteins to migrate through the gel according to their weight. Smaller proteins migrate more quickly, allowing size‐based migration and separation. This is followed by Coomassie blue staining Western blotting: Verify protein markers post‐membrane coating. The separated proteins are transferred to specific membranes (e.g., nitrocellulose), and analyzed using highly specific antibodies	Confirm the preservation of the specific membrane protein markers and protein profiles once the membrane nanovesicle is coated onto the NP core (to ensure the biofunctionality post‐membrane coating)	10, 59, 77, 83, 84

Abbreviations: CLSM, confocal laser scanning microscopy; DLS, dynamic light scattering; ELS, electrophoretic light scattering; NP, nanoparticle; NTA, nanoparticle tracking analysis; PDI, polydispersity index; SDS‐PAGE, sodium dodecyl sulfate‐polyacrylamide gel electrophoresis; SEM, scanning electron microscopy; TEM, transmission electron microscopy.

#### 4.3.2 Biological characterization

Besides, a thorough analysis of the biological properties of the final nanosystem (after the coating process) is also required to investigate the surface protein composition and protein profile of the coated NPs, which should be similar to that of the extracted biomembrane, to guarantee the bioactivity and functionality of the coated NPs post‐membrane coating.^10^, ^73^

For analyzing the protein surface markers and the protein profile of the coated NPs, techniques such as SDS‐PAGE (sodium dodecyl sulfate‐polyacrylamide gel electrophoresis) followed by staining, and Western Blotting, are usually employed (Table 5).^59^

## 5 POTENTIAL STRATEGIES FOR FINE‐TUNING THE PROPERTIES OF BIOMIMETIC NANOPARTICLES: INNOVATIVE APPROACHES

As the research in the nanoworld continues, so does the search for innovative approaches with the potential to enhance the multifunctionality of the biomimetic NPs. The goal of these strategies is to “fine‐tune” the properties of these advanced nanosystems, by enhancing both their active tumor‐targeting and therapeutic efficacy capabilities, beyond those naturally expected from the biomembrane coating and therapeutic cargo loading into NPs.^59^, ^68^

### 5.1 Biomembrane functionalization approaches: Enhancing the tumor‐targeting capabilities of biomembrane‐coated nanoparticles

Biomembrane functionalization techniques represent fundamental surface engineering approaches that modify the surface repertoire of biomembranes through the incorporation of functional moieties (e.g., peptides, aptamers, and antibodies). These modifications are essential for improving targeting to tumors and ensuring the bioactivity of the biomimetic NPs.^59^, ^68^

The operation of biomembrane surface functionalization is usually conducted after biomembrane extraction and subsequent membrane coating onto NPs (post‐membrane coating), so that the functional ligands are inserted on the outer surface of the biomembranes. Nevertheless, this purification process can be laborious, and there is the possibility of affecting the physiochemical features of the surface‐engineered biomembrane.^68^ An alternative strategy consists in conducting this process before the biomembrane extraction and subsequent membrane coating onto NPs (pre‐membrane coating), by functionalizing directly the surface membrane of living cells. This strategy is more convenient in terms of purification, but there is the possibility of affecting the final orientation of the surface‐engineered biomembrane.^68^

In Table 6 we summarize and provide a comparative overview of the different surface engineering approaches commonly employed for biomembrane surface‐functionalization with functional ligands, including (A) lipid insertion; (B) chemical conjugation; (C) genetic modification; (D) metabolic engineering; and (E) non‐covalent adsorption.^59^, ^68^

**TABLE 6:** Comparative overview of the methods employed for biomembrane‐surface functionalization.

Method	Mechanistic principle	Schematic depicting	Advantages	Limitations	Refs.
Lipid insertion (A)	Firstly, the functional ligands are chemically linked to a lipid molecule (lipid anchor). Then, the lipid‐ligand conjugates are attached to the membrane surface via lipid insertion (Lipid‐Lipid interactions) Can be performed directly on living cells and on membrane vesicles For example, DSPE‐PEG‐ligand, DOPE‐PEG‐ligand…	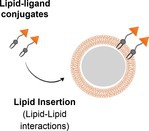	Non‐invasive and non‐disruptive Easy insertion Preserves the membrane surface functionality and protein integrity ↓ preparation complexity	Not suitable for large ligands and transmembrane protein receptors More challenging to perform in larger molecules (e.g., antibodies)	59, 68
Chemical conjugation (B)	Functional ligands are anchored onto the membrane surface by means of strong and covalent interactions (chemical reactions) For example, thiol‐maleimide coupling, EDC/NHS coupling, azide‐alkyne cycloaddition, and amidation chemistry	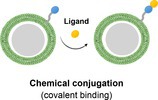	↑ production yields Quick and easy to perform strategy ↑ compatibility with biomolecules	Use of chemical reagents ↑ potential to damage the membrane functionality and protein integrity	59, 68
Genetic modification (C)	Relies on the genetic manipulation of the source living cells Inserting functional ligands onto the membrane surface by the plasmids‐mediated gene transfection and expression	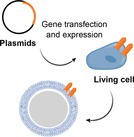	Precise and non‐invasive strategy ↓ damage to the membrane integrity Potential for large‐scale production ↓ costs	↑ complexity and challenging operational steps Not suitable for small ligands	59, 68
Metabolic engineering (D)	Harnessing the natural biosynthetic pathways of living cells to incorporate functional ligands onto the membrane surface Firstly, the metabolic substrates are covalently attached to functional ligands, and then the conjugates are introduced in the cellular pathways For example, sialic acid, GaINAc salvage, fucose salvage, and CDP‐choline metabolic pathways	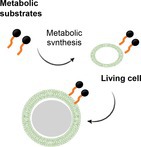	Non‐invasive strategy Takes advantage of the endogenous and naturally occurring cellular processes	Difficult to control	59, 68
Non‐covalent adsorption (E)	Functional ligands are anchored onto the membrane surface by weak and non‐covalent interactions For example, Hydrogen bonding, electrostatic interactions, van der Waals forces, and hydrophobic interactions	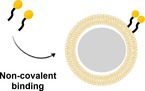	Non‐invasive strategy Preserves the membrane surface functionality and protein integrity	↓ long‐term stability ↓ incorporation efficiency of the functional moieties onto the membrane	59, 68

Abbreviations: ↑, enhancement; ↓, reduction.

### 5.2 Combinatorial therapies: Improving the therapeutic efficacy of biomembrane‐coated nanoparticles

Exploiting biomembrane‐coated nanosystems for cancer monotherapies, for instance the single‐use of chemotherapy, immunotherapy, or, for example, phototherapy, has been producing insufficient results in eliminating gastrointestinal cancers. Due to the biological and immunological complexity of the gastrointestinal tumor microenvironment, a synergistic and holistic approach with a multi‐targeted action is required to face all the challenges encountered in this complex environment of drug‐resistant tumor cells, highly activated immunosuppressive cells, and a fibrotic stroma blocking the access of the therapy to tumor cells.^18^, ^59^

In view of this background, synergistic therapies combining multiple anti‐cancer approaches have also been investigated with the aim of addressing these challenges and significantly increasing anti‐tumor efficacy compared to the respective monotherapies.^35^ The trend is to combine synergistic therapies in a single nanosystem, either by (1) combining different compounds in the same nanosystem (to provide different anti‐cancer effects), or (2) by capitalizing on the intrinsic properties of NPs. In the latter we can emphasize, for example, the intrinsic anti‐tumor properties of the TPGS micellar system,^86^ and the phototherapeutic potential of bismuth NPs by absorbing NIR light radiation.

## 6 CELL‐MIMICKING NANOTECHNOLOGICAL APPROACHES FOR GASTROINTESTINAL CANCERS

Presently, cell membrane‐coated NPs have attracted considerable attention as promising cell‐mimicking nanoplatforms for cancer therapy and diagnosis, and this review highlights their biomedical applicability for the treatment of gastrointestinal cancers. These advanced drug delivery systems have been exploited for the treatment of several gastrointestinal cancers, namely colorectal, gastric, liver, esophageal, and pancreatic cancer (Table 7).

**TABLE 7:** Summary of the studies employing cell membrane‐coated biomimetic nanocarriers for the treatment of gastrointestinal cancers.

Cancer type	Therapeutic strategy	Cell membrane source	Inner core	Drug(s)	Coating method	Size/zeta potential	In vivo tumor Model	Outcomes	Refs.
Colorectal cancer	Chemotherapy	Murine colon carcinoma HT‐29 cell membrane	Gelatin nanogel	Paclitaxel	Co‐extrusion through 200 nm porous membranes	103.6 nm −33.4 mV	HT‐29 tumor‐bearing mouse model	Immune escape Homologous tumor‐targeting ↑ tumor accumulation ↑ antitumor efficacy ↓ systemic side effects	87
	Chemotherapy	Mesenchymal stem cell membrane	Superparamagnetic iron oxide NP	Doxorubicin	Co‐extrusion through 200 nm porous membranes (10 times)	<170.0 nm	MC38 tumor‐bearing C57BL/6 mouse model	Tumor targeting features ↑ uptake by MC38 colon cancer cells ↑ antitumor effects compared to non‐coated counterparts ↓ doxorubicin‐related systemic side effects	88
	Chemotherapy	Red blood cell membrane	Hollow mesoporous organosilica NP	Doxorubicin	Selective etching technique	295 ± 1.3 nm −17 mV	C26 tumor‐bearing BALB/c mouse model	Significant tumor growth blockade ↑ tumor‐targeting ability ↑ In vivo model survival ↓ Drug‐induced toxicity	89
	Chemotherapy	Platelet membrane	Gelatin nanogel	Irinotecan	Co‐extrusion through a 200 nm porous membranes	102.2 nm −29.6 mV	HT‐29 tumor‐bearing BALB/c mouse model	Tumor targeting features ↑ tumor accumulation ↑ antitumor efficacy ↓ tumor growth ↓ irinotecan‐related systemic side effects	90
	Chemotherapy	iRGD‐modified red blood cell membrane	NP	Oxaliplatin and juglone	Sonication (10 min)	—	HCT‐116 tumor‐xenografted nude mouse model	↑ tumor‐targeting ability provided by the iRGD tumor targeting peptide ↑ tumor penetration Synergistic antitumor effects of both compounds	91
	Chemotherapy	Red blood cell membrane	HPA‐conjugated PLGA NP	Chloroquine and artesunate	Co‐extrusion and Sonication	160.07 ± 4.75 nm −6.90 ± 2.73 Mv	CT‐26 tumor‐bearing orthotopic mouse model	↑ Tumor site accumulation Tumor growth blockade M2‐like TAMs repolarization inhibition	92
	Colon cancer‐targeted anticancer drug delivery	Red blood cell membrane	PLGA NP	Gambogic acid	Co‐extrusion through 200 nm porous membranes (10 times)	~163.0 nm	Subcutaneous SW480‐tumor‐bearing nude mouse model	↑ biocompatibility Overcoming the poor water solubility of gambogic acid ↑ antitumor efficacy Inhibition of SW480 cells growth	93
	Colon cancer‐targeted anticancer drug delivery	Red blood cell membrane dual functionalized with anti‐EGFR‐iRGD	PLGA NP	Gambogic acid	Co‐extrusion through 200 nm porous membranes (10 times)	~153.0 nm	Caco‐2 tumor‐bearing nude mouse model	↑ tumor‐targeting ability provided by the protein anti‐EGFR‐iRGD ↑ tumor accumulation in high EGFR‐expressing colon cancer cells ↑ antitumor efficacy	94
	Starvation‐activated colon cancer therapy	Red blood cell membrane	ZIF‐8 NP	GOx and cytotoxic prodrug tirapazamine	Sonication (5 min) and co‐extrusion through 400 and 200 nm porous membranes	243.0 nm −29.6 mV	CT26 tumor‐bearing mouse model	↑ blood circulation time than non‐coated NPs Immune evasion ↑ tumor targeting ↑ antitumor efficacy against colon cancer	95
	Ferroptosis	Red blood cell membrane	PCL‐PEG NP	Resveratrol	Sonication (20–30 min)	160.91 ± 0.63 nm −31/64 ± 0.91 Mv	HT29 xenografted model	Immune evasion Excellent cancer‐fighting property ↑ NP half‐life ↑ Tumor targeting ↑ Targeted bioaccumulation	96
	PTT	CT26 colon cancer cell membrane	Metal bismuth NP	—	Co‐extrusion	≈50.0 nm −22.6 mV	CT26 tumor‐bearing mouse model	↑ uptake by homotypic CT26 cancer cells ↑ NIR conversion efficiency ↑ photothermal ablation of cancer cells	97
	Bimodal NIR fluorescence and MRI‐guided PTT	Red blood cell membrane	Superparamagnetic nanoclusters	Cypate (photothermal agent)	Sonication	—	HCT‐116 tumor‐bearing mouse model	↑ tumor‐homing features ↑ NIR conversion efficiency Photothermal ablation of cancer cells NIR fluorescence and MRI bimodal imaging	98
	Synergistic PTT‐immunotherapy	Macrophage cell membrane functionalized with an anti‐PDL1 antibody	Hollow gold nanocage nanocomposite	Galunisertib	Co‐extrusion	57.0 nm −18.0 mV	CT26 tumor‐bearing mouse model	↑ tumor accumulation Efficient NIR‐induced photothermal ablation of cancer cells Immunogenic death of colon cancer cells ↑ antitumor efficacy	99
	Chemotherapy and imaging‐guided PTT	CT26 colon cancer‐macrophage hybrid cell membrane	ZGGO@SiO_2_ NP	IR825 (photothermal agent) and irinotecan	Co‐extrusion through 200 nm porous membranes (5 cycles)	135.3 nm −32.6 mV	CT26 tumor‐bearing mouse model	Immune evasion ↑ tumor‐homing features NIR‐induced thermal cancer cell death Imaging‐guided cancer ablation via PTT and chemotherapy	100
	PDT	Platelet membrane	Hollow MnO_2_ NP	DCPy (photosensitizer)	Co‐extrusion (11 times through 200 nm porous membranes)	~120 nm ~−20 mV	Orthotopic CT26 tumor‐bearing mouse model	Excellent stability ↑ Tumor‐specific targeting PDT‐mediated anticancer effects ↑ Tumor growth inhibition	101
	Synergistic PTT‐SDT	CT26 cancer cell membrane	Cu_9_ S_8_ nanospheres	Hemoporfin (sonosensitizer)	Sonication	~215 nm −20.6 mV	CT26 tumor‐bearing BALB/c mouse model	Strong targeting ability ↑ Biocompatibility ↑ Tumor site accumulation ↑ Blood circulation half‐life ↑ Tumor growth inhibition	102
	SDT	HCT‐116 cancer cell membrane	Platinum nanozymes pre‐doped hollow poly‐dopamine NP	Chloroquine and Ce6 (sonosensitizer)	Co‐extrusion Sonication	—	HCT‐116 tumor‐bearing mouse models	↑ ROS generation ↑ Tumor site accumulation ↓ Hypoxic condition Apoptosis and ferroptosis induction Autophagy inhibition	103
	CDT‐SDT combinatorial therapy	CT26 cancer cell membrane	BSA‐modified Fe_3_O_4_ NP	Ce6 (sonosensitizer)	Sonication	—	CT26 tumor‐bearing mouse model	↑ ROS generation Apoptosis induction Tumor growth blockade	104
	SDT and reprogramming immunosuppressive tumor microenvironment	Neutrophil‐Red blood cell hybrid membrane	ZIF‐8 NP	Ce6 (Sonosensitizer)	Sonication	189.23 nm −40.11 mV	Orthotopic colorectal cancer mouse model	↑ ROS generation Neutrophil chemotaxis inhibition M1‐to‐M2 macrophage polarization suppression ↓ Tumoral hypoxia ↑ In vivo survival rate	105
	Gene therapy	HCT‐116 cancer cell membrane	PLGA‐b‐PEG DC‐cho NP	Doxorubicin and miR‐190‐Cy7	Co‐extrusion	~100 nm	HCT‐116 xenograft tumor‐bearing nude mouse model	Tumor growth and angiogenesis inhibition TGF‐β signaling pathway suppression ↑ Tumor targeting ↓ VEGF expression	106
	Gene therapy	MC38 colon cancer cell membrane	Poly (citrate‐peptide) NP	miR365 mimic	—	~295.0 nm	MC38 tumor‐bearing mouse model	↓ macrophage uptake Homologous tumor‐targeting ability Efficient delivery of miR365 to targeted tumor sites ↓ Ki67/Bcl2 expression ↓ tumor growth	107
	Radiotherapy and targeted PDT	HCT‐116 cancer cell membrane	Platinum‐integrated hollow polydopamine NP	Apoptin (a radiosensitizer) and verteporfin (a photosensitizer)	Mixing and stirring in ice bath	−24.8 mV	HCT‐116 tumor‐bearing mouse model	↑ Tumor‐specific targeting Synergistic antitumor effects via radiotherapy and PDT ↓ tumor growth	108
	Radiotherapy and immunotherapy	Neutrophil membrane	PLGA NP	R837 (Imiquimod, a TLR7 agonist)	Co‐extrusion	250.2 ± 54.7 nm −18.43 ± 1.9 mV	CT26 tumor‐bearing mouse model	Specific tumor targeting Antitumor immunity induction Low cytotoxicity ↓ Tumor size and volume	109
Gastric cancer	Chemotherapy	Cytotoxic CD8^+^ T‐lymphocyte membrane	PLGA NP	Paclitaxel	Co‐extrusion through 200 nm porous membranes	165.9 nm	MKN‐45 tumor‐bearing mouse model	↑ blood circulation time Tumor‐targeting ability ↑ tumor accumulation triggered by application of low‐dose irradiation Tumor growth inhibition rate of 88.5% (when applied local low‐dose irradiation)	110
	Chemotherapy	Red blood cell membrane	PLGA NP	Paclitaxel and triptolide	Sonication (5 min)	154.0 nm ≈−30.0 mV	—	↑ blood circulation time Immune escape ↑ in vitro antitumor efficacy against gastric cancer than free drugs	111
	Intraperitoneal chemotherapy	Red blood cell membrane	PEG‐modified bovine serum albumin NP	Paclitaxel	Sonication	133.1 nm −10.0 mV	Subcutaneous MKN45 tumor‐bearing mouse model with peritoneal dissemination	Local intraperitoneal chemotherapy ↑ retention at peritoneal area Sustained drug release ↓ tumor growth	112
	Synergistic chemotherapy and PTT	Red blood cell‐gastric cancer cell hybrid membrane	GOQD	ICG (a photosensitizer) and gamabufotalin (an anticancer drug)	—	—	Gastric cancer bearing mouse model	↑ Tumor‐specific targeting Synergistic antitumor effects via chemotherapy and PTT ↓ tumor growth	113
	Suppression of the VEGFR2/STAT3 pathway	HGC‐27 gastric cancer cell‐red blood cell hybrid membrane	pH‐responsive liposome (Liposome‐PEO, LP)	Apatinib and cinobufagin	Co‐extrusion	108.0 nm −7.5 mV	HGC‐27 tumor‐bearing nude BALB/c mouse model	↑ blood circulation time ↑ tumor‐homing features ↓ tumor growth ↓ tumor metastasis by suppression of the VEGFR2/STAT3 pathway ↑ antitumor efficacy	114
	Ferroptosis‐mediated ICD, chemotherapy, CDT	Gastric cancer cell membrane	Mn^2+^ − doped mesoporous silica NP	Cisplatin	—	125.90 ± 2.52 nm (in FBS) 128.03 ± 1.63 nm (in DMEM) −39.83 ± 1.46 mV	Gastric cancer bearing mouse model	Intracellular GSH depletion Nuclear DNA damage Highly reactive hydroxyl radical generation Tumor growth inhibition Tumor relapse suppression Ferroptosis induction ↑ LPO levels	115
	PDT	SGC7901 gastric cancer cell membrane	Silica NP	Ce6 (photosensitizer)	Sonication (100 W, 5 min)	115.6 nm −30.4 mV	SGC7901 tumor‐bearing mouse model	↑ tumor‐targeting features to homotypic SGC7901 cells Efficient ROS‐induced ablation of cancer cells upon NIR irradiation ↑ antitumor efficacy	116
	US‐triggered SDT and chemotherapy	AGS cancer cell membrane	ZIF‐8 NP	Tirapazamine and Ce6 (photosensitizer)	Sonication	253 nm −16.4 mV	AGS tumor‐bearing nude mouse model	Pyroptosis induction Cancerous cells' growth suppression ↑ Tumor‐killing properties ↑ Hypoxia in TME ↑ ROS generation	117
Liver cancer	Chemotherapy	HepG2 hepatocellular carcinoma cell membrane	PLGA NP	Doxorubicin	Sonication (15 min)	5.0 mV	HepG2 tumor‐bearing nude mouse model	↓ Tumor volume ↑ Targeting ability High drug loading capacity ↑ NP's stability ↑ NP's immunocompatibility	118
	Chemotherapy	HepG2 hepatocellular carcinoma cell membrane	PLGA NP	Doxorubicin	Co‐extrusion through 100 nm porous membranes (10 times)	≈100.0 nm −29.5 mV	HepG2 tumor‐bearing xenograft mouse model	↑ Antitumor effects ↑ Doxorubicin cellular uptake ↓ Systemic toxicity Prolonged blood circulation ↑ Drug accumulation at the tumor site	119
	Chemotherapy	Red blood cell membrane	Pectin‐doxorubicin conjugates (self‐assembled into NPs)	—	Ultrasonication	151.4 nm	BEL‐7402 tumor‐bearing BALB/c nude mouse model	Tumor‐targeted accumulation ↑ Antitumor effects via doxorubicin‐mediated chemotherapy Tumor cells' growth inhibition	120
	Chemotherapy	Folate‐modified SMMC‐7721 liver cancer cell membrane	Paclitaxel nanocrystals	Paclitaxel	Sonication and incubation (2 h at 37°C)		SMMC‐7721 tumor‐bearing BALB/c mouse model	↑ Targeting ability ↑ Accumulation in tumor tissues Tumor cells' growth inhibition	121
	Chemotherapy	SMMC‐7721 liver cancer cell membrane	pH‐sensitive polymeric bionic NP	Lenvatinib	Sonication	134.0 nm ~−42.0 mV	SMMC‐7721 tumor‐bearing mouse model	↑ Biocompatibility ↑ Drug loading capacity ↑ Tumor site accumulation Long term stability Long retention time Precise tumor targeting	122
	Liver cancer‐targeted anticancer drug delivery	Huh‐7 liver cancer cell‐platelet hybrid membrane	Lyotropic liquid crystalline lipid NP	Sorafenib and triptolide	Sonication (100 W, 2 min)	192.9 nm −20.1 mV	Huh‐7 tumor‐bearing BALB/c mouse model	Long circulation function Cancerous cells' growth blockade ↑ Tumor targeting ↑ Cancer cells' apoptosis	123
	Liver cancer‐targeted anticancer drug delivery	Erythrocyte membrane	mPEG‐PLA NP	Gambogic acid	Co‐extrusion	102.3 ± 3.1 nm	HepG2 tumor‐bearing mouse model	Longer retention time ↑ Biosafety ↑ Stability ↑ Cancerous cells' inhibition Improved drug water solubility	124
	Liver cancer‐targeted anticancer drug delivery	Platelet membrane	Chitosan oligosaccharide‐PLGA NP	Bufalin	Nanoprecipitation	~192.0 nm ~−28.0 mV	H22 tumor‐bearing mouse model	Tumor cell growth blockade ↑ Tumor accumulation	125
	Liver cancer‐targeted anticancer drug delivery	HepG2 cancer cell membrane	Ferrous ion doped ZIF‐8 NP	Dihydroartemisinin	Co‐extrusion	90–100 nm −22.3 mV	HepG2 tumor‐bearing BALB/c mouse model	Strong tumor specificity Improved immune evasion ↑ ROS generation ↑ Anti‐cancer effects ↑ Biocompatibility ↓ Side effects	126
	Gene therapy‐NIR irradiation	Red blood cell membrane	Hyaluronic acid surface‐functionalized AuNP	miR‐181b	Sonication Extrusion	176.67 ± 4.2 nm −15.17 ± 2.5 mV	SMMC‐7721 tumor‐bearing xenograft nude mouse model	Enzymatic degradation protection Improved cellular uptake Prolonged circulation time Tumor growth blockade Sustained miRNA release Improved in vivo biodistribution	127
	PTT	CAR‐T cell membrane targeting glypican‐3 (GPC3^+^)	Mesoporous silica NP	IR780 dye	Co‐extrusion through 200 nm porous membranes (11 times)	≈110.0 nm −6.7 mV	Huh‐7 tumor‐bearing BALB/c mouse model	↑ Tumor targeting ability ↓ Systemic toxicity ↑ Photothermal responses	128
	PTT	SMMC‐7721 liver cancer cell membrane	iRGD peptide surface functionalized drug delivery system	Sepantronium bromide and graphene quantum dots (PTT agents)	Co‐extrusion	—	SMMC‐7721 tumor‐bearing mouse model	Effective tumor targeting ↑ Biocompatibility	129
	Synergistic chemo‐PTT	RAW 264.7 macrophage‐H22 hepatic cancer cell hybrid membrane	Hollow cooper sulfide NP	Sorafenib	Sonication	≈210.0 nm	Hepatocellular carcinoma‐bearing mouse model	Selective tumor accumulation Immune escape Improved drug loading capacity ↓ Tumor proliferation ↓ Tumor angiogenesis	130
	Synergistic chemo‐PTT	Platelet membrane	Polypyrrole NP	Doxorubicin	Co‐extrusion through 200 nm porous membranes (11 times)	≈110.0 nm ≈−11.0 mV	Huh 7 tumor‐bearing BALB/c mouse model	Cancer cells' growth and metastasis blockade Improved local chemo‐PTT effectiveness	131
	PDT and NIR fluorescence imaging	Naïve neutrophil membrane	PLGA NP	Photosensitizer hypocrellin B	Co‐extrusion	140.0 nm −24.8 mV	KM mouse model	Inhibition of JUNB expression ↑ ROS production ↑ Anti‐tumor effects ↓ Immune elimination	132
	PDT	Transferrin‐functionalized HepG2 cancer cell membrane	PEG‐PLGA NP	Photosensitizer hypocrellin B	Co‐extrusion	**—**	HepG2 tumor‐bearing BALB/c mouse model	Long‐term stability Improved biocompatibility ↑ Drug loading capacity ↑ Tumor targeting ↓ Toxicity	133
	Synergistic cancer starvation therapy and chemotherapy	HepG2 cancer cell membrane	GOx surface‐modified hollow mesoporous organosilica NP	Doxorubicin and O_2_‐loaded perfluorocarbon	Sonication	152.0 nm −16.1 mV	Subcutaneous HepG2 tumor mouse model	↑ Tumor‐specific targeting ↑ Doxorubicin‐mediated chemotherapy efficacy Improved local chemo‐cancer starvation therapy efficacy Cancer cells' growth blockade	134
	Suppression of postoperative HCC relapse	Anti‐PDL1 antibody‐modified platelet membrane	MSN	Sorafenib	Sonication	99.8 nm −18.4 mV	Orthotopic HCC mouse model	Strong anti‐angiogenic effects Cancer‐fighting properties ↓ Post‐surgical HCC relapse ↑ Tumor/blood site accumulation	135
	Radiotherapy and imaging	M1 macrophage membrane	Nanodot	BiOI (Radiosensitizer and contrast agent)	Co‐extrusion	—	Hepa1‐6 xenograft mouse model	Low systemic toxicity Rapid renal clearance Prolonged circulation time Tumor proliferation blockade Excellent CT imaging ability ↑ Tumor targeting ↑ Radiosensitivity ↓ NPs' toxicity	136
	Ferroptosis	HCC cell membrane	Fe_3_O_4_ magnetic NP	Arsenic trioxide (ferroptosis inducer)	Co‐extrusion through 200 nm polycarbonate membranes	93.0 nm −34.0 mV	Hep3B tumor‐bearing mouse model	Tumor targeting features ↑ Tumor accumulation GPX4 inhibition, and ↑ intracellular lipid peroxide ↑ HCC tumor suppression and cell death via ferroptosis	137
Esophageal cancer	Chemotherapy	Esophageal cancer TE10 cell membrane	PLGA NP	Doxorubicin and curcumin	Co‐extrusion	177.0 nm ≈−26.0 mV	TE10/DOX tumor‐bearing BALB/c mouse model	↑ Tumor cells inhibition ↑ Tumor targeting ↑ Biosafety	138
	Synergistic chemotherapy and gene therapy	Proinflammatory leukocyte membrane	Lipid nanovector	Doxorubicin and siLPCAT1	Sonication	136.0 nm −21.2 mV	KYSE‐150 tumor‐bearing BALB/c mouse model	Blockade of tumor cells' growth, migration, and metastasis Improved blood circulation time ↑ Tumor targeting No side effects No toxicity	139
	ESCC‐targeted PDT	Thylakoid‐EC109 esophageal cancer cell hybrid membrane	PLGA NP	Gambogic acid	Sonication (5 min, 4°C) and co‐extrusion	205.0 nm ∼−31.8 mV	EC109 tumor‐bearing mouse model	ESCC tumor‐targeting ability ↑ tumor accumulation ↓ Tumoral hypoxia ↓ GSH intratumoral levels PDT‐mediated tumor ablation ↑ ESCC tumor growth suppression and killing	140
Pancreatic cancer	Chemotherapy	BxPC‐3 pancreatic cancer cell membrane	PLGA NP	FOLFIRINOX (5‐Fluorouracil, irinotecan and oxaliplatin)	Repeated co‐extrusion	149.5 nm −2.73 mV	BxPC‐3 tumor‐bearing BALB/c nude mouse model	Prolonged blood circulation ↑ Immune escape ↑ Tumor site penetration and accumulation ↓ Chemo‐induced side effects	141
	Chemotherapy	RAW 264.7 macrophage cell membrane	PLGA NP	Gemcitabine	Co‐extrusion (20 times)	≈192.0 nm −16.8 mV	PANC‐1 tumor‐bearing mouse model	Cancerous cells' growth and angiogenesis blockade Phagocytosis escape Passive targeting	142
	Chemotherapy	Naïve neutrophil membrane	PEG‐PLGA NP	Celastrol	Co‐extrusion	167.4 nm	GFP‐Panc02 tumor‐bearing C57BL/6 mouse model	Overcoming blood‐pancreas barrier Selective drug accumulation ↑ survival rate Minimized liver metastasis	143
	NIR‐triggered PTT and fluorescence imaging	Erythrocyte membrane	ZIF‐8 (MOF) NP	ICG (photosensitiser)	Electrostatic adsorption	122 ± 1.3 nm −4.14 mV	Panc02 tumor‐bearing C57 mouse model	↑ Tumor targeting ↑ Tumor site accumulation ↑ Cancerous cells' growth	144
	NIR‐II fluorescence imaging, chemotherapy, PTT and PDT	SW1990 pancreatic cancer cell membrane	Liposomes	Doxorubicin and ICG (photosensitizer and photothermal agent)	Co‐extrusion	106.3 ± 2 nm −18.2 ± 0.4 mV	SW1990 tumor‐bearing BALB/c mouse model	Light‐controlled drug release Improved PTT and PDT ↓ Tumor growth	145
	Tumor microenvironment modulation plus PTT	Red blood cell membrane	Gold nanorod	Cyclopamine	Sonication (100 W, 2 min)	−32.2 mV	Capan‐2 tumor‐bearing xenograft model	Strong tumor microenvironment modulation ↑ colloidal stability Long circulation time ↑ PTT efficacy	146
	Tumor microenvironment modulation for improved chemotherapy	Red blood cell membrane	PLGA NP	Cyclopamine or paclitaxel (separately)	Sonication (100 W, 2 min)	80.4 nm −29.5 mV	Capan‐2 tumor‐bearing mouse model	↑ Biocompatibility Long circulation time Strong tumor microenvironment modulation Improved tumor perfusion Tumor growth blockade	147
	Chemotherapy, molecular‐targeted therapy and anti‐fibrotic gene therapy (stroma modulation)	SW1990 pancreatic cancer cell‐RAW264.7 macrophage hybrid membrane	GEM‐SS‐PC polymer prodrug	Erlotinib and siIRAK4	—	200 nm −16.4 mV	Orthotopic SW1990 tumor‐bearing BALB/c mouse model	Fibrotic pancreatic stroma modulation ↓ Cancerous cells' growth and liver metastasis blockade ↑ Survival rate in vivo	148
	Chemotherapy	BxPC‐3 cancer cell membrane	Nanorods	Doxorubicin	Ultrasonic incubation	130 nm ~−20 mV	BxPC‐3/Pancreatic stellate cell hybrid tumor‐bearing nude mouse models	Excellent immune escape Apoptosis induction Rapid ECM penetration ↑ Tumor eradication ↑ Tumor accumulation at extracellular site	149
	Delivery of PROTAC‐induced PDEẟ degrader	PATU‐8988 pancreatic cancer cell membrane	Bionic nanosized drug delivery system	PIPD	Sonication	124.8 nm −7.59 mV	DMEM (50% FBS) mimicking the in vivo status	Controlled drug release Apoptosis induction Tumor growth suppression via RAS signaling pathway blockade ↑ Biocompatibility and immunocompatibility ↑ Serum stability	150
	Synergistic nsPEF therapy and chemotherapy	Neutrophil membrane	Liposomes	Gemcitabine	Sonication (2 min)	~170 nm ~−19 mV	Panc02 tumor‐bearing C57BL/6 mouse model	Tumor growth blockade ↑ TNF‐α release ↑ Tumor targeting	151

Abbreviations: BiOI, bismuth oxyiodide; BSA, bovin serum albumin; CAR, chimeric antigen receptor; CDT, chemo‐dynamic therapy; Ce6, chlorin e6; CT, computed tomography; Cy7, cyanine 7; β‐CD: β‐cyclodextrin; DCPy, 2,6‐dichloropyridine; DMEM, dulbecco's modified eagle medium; ECM, extracellular matrix; ESCC, esophageal squamous cell carcinoma; GOQD, graphene oxide quantum dot; GOx, glucose oxidase; GPX4, glutathione peroxidase‐4; GSH, glutathione; HCC, hepatocellular carcinoma; HPA, hydroxymethyl phenylboronic acid; ICG, indocyanine green; siIRAK4, siRNA targeting the *IRAK4* gene; iRGD, RGD internalizing peptide (a tumor‐penetrating peptide); JUNB, junB Proto‐oncogene; LPO, lipid peroxides; miRNA, microRNA; MRI, magnetic resonance imaging; MSN, mesoporous silica nanoparticle; NIR, near‐infrared; NP, nanoparticle; PCL, poly(ɛ‐caprolactone); PDT, photodynamic therapy; PEG, polyethylene glycol; PEI, polyethylenimine; PIPD, PROTAC‐induced PDEẟ degrader; PLGA, poly (lactic‐*co*‐glycolic acid); PROTAC, proteolysis‐targeting chimeric; PTT, photothermal therapy; ROS, reactive oxygen species; SDT, sono‐dynamic therapy; siLPCAT1, siRNA targeting the *LPCAT1* gene; siRNA, small interfering RNA; TAMs, tumor‐associated macrophages; TGF‐β, transforming growth factor‐β; TLR7, toll‐like receptor 7; TME: tumor microenvironment; TNF‐α, tumor necrosis factor‐α; ZGGO@SiO_2_, persistent luminescence mesoporous silica NPs; ZIF‐8, zeolitic imidazolate framework‐8; ↑, enhancement; ↓, reduction.

In this field, these biomimetic NPs coated by either cell membranes, exosome membranes, bacterial membranes, or bacterial OMV membranes assume an important role in ensuring the effective and tumor‐targeted delivery of several compounds for improved gastrointestinal cancers‐localized theranostics. Some examples include, among others, imaging agents, phytocompounds (e.g., gambogic acid, resveratrol), chemotherapeutics (e.g., oxiplatin, paclitaxel, doxorubicin), immunomodulators (e.g., imiquimod), genetic material (e.g., miR‐190‐Cy7, miR365 mimic), photosensitizers (e.g., DCPy, hypocrellin B), and photothermal agents (e.g., cypate, IR825) for cancer phototherapy, radiosensitizers (e.g., apoptin, BiOI), sonosensitizers (e.g., hemoporfin), ferroptosis inducer agents (e.g., arsenic trioxide), and tumor microenvironment modulators (e.g., cyclopamine) for enhanced antitumor efficacy.

### 6.1 Colorectal cancer

Colorectal cancer has a significant impact worldwide. It is the third most common type of cancer and ranks as the second deadliest cancer globally, being therefore, associated with a high incidence and cancer‐related mortality.^152^ The first‐line treatment for colorectal cancer in the early stages of the disease is surgical resection, while at advanced stages, including metastatic or inoperable cases, chemotherapy is the standard therapeutic approach.^87^, ^99^ Nevertheless, this strategy is limited, mostly due to the low tumor specificity of chemotherapeutic drugs, which results in severe side effects and off‐target toxicity in healthy cells. To overcome these limitations and reduce chemotherapy side effects, cloaking cell membranes onto NPs has been reported, with the primary goal of increasing therapeutic efficacy and safety of traditional chemotherapy.^87^

In a study aimed at developing a biomimetic core‐shell nanostructure for targeted chemotherapy against colorectal carcinoma, paclitaxel‐loaded gelatin nanogels were coated with a tumor cell membrane derived from homotypic HT‐29 tumor cells.^87^ In the in vitro studies, the resulting biomimetic nanosystem was shown to efficiently avoid macrophage‐mediated immune clearance, while in vivo studies showed that the nanosystem could efficiently target tumor cells, due to the homotypic tumor‐targeting features of the membrane coating. This could improve the accumulation of anticancer drug paclitaxel at homotypic tumor tissues and reduce its systemic side effects. Furthermore, after intravenous injection into a mouse model, the biomimetic nanosystem exhibited the most remarkable antitumor efficacy, inducing the greatest suppression of tumor growth in vivo, thus proving to be a promising nanosystem for targeted chemotherapy of colorectal carcinoma.^87^

In another different study, doxorubicin‐loaded superparamagnetic iron oxide NPs were cloaked with a mesenchymal stem cell membrane for targeted chemotherapy of colorectal carcinoma.^88^ Due to the mesenchymal stem cell membrane coating, the nanoassembly was endowed with enhanced tumor‐targeting and immune evasion features, which resulted in greater accumulation of doxorubicin at target tumor tissues and superior antitumor efficacy, as demonstrated by the greater inhibition of tumor growth in vivo. In summary, the biomimetic nanosystem showed improved cellular uptake efficiency, thus enhancing the antitumor efficacy and reducing the doxorubicin‐related systemic side effects.^88^

**FIGURE 3:**
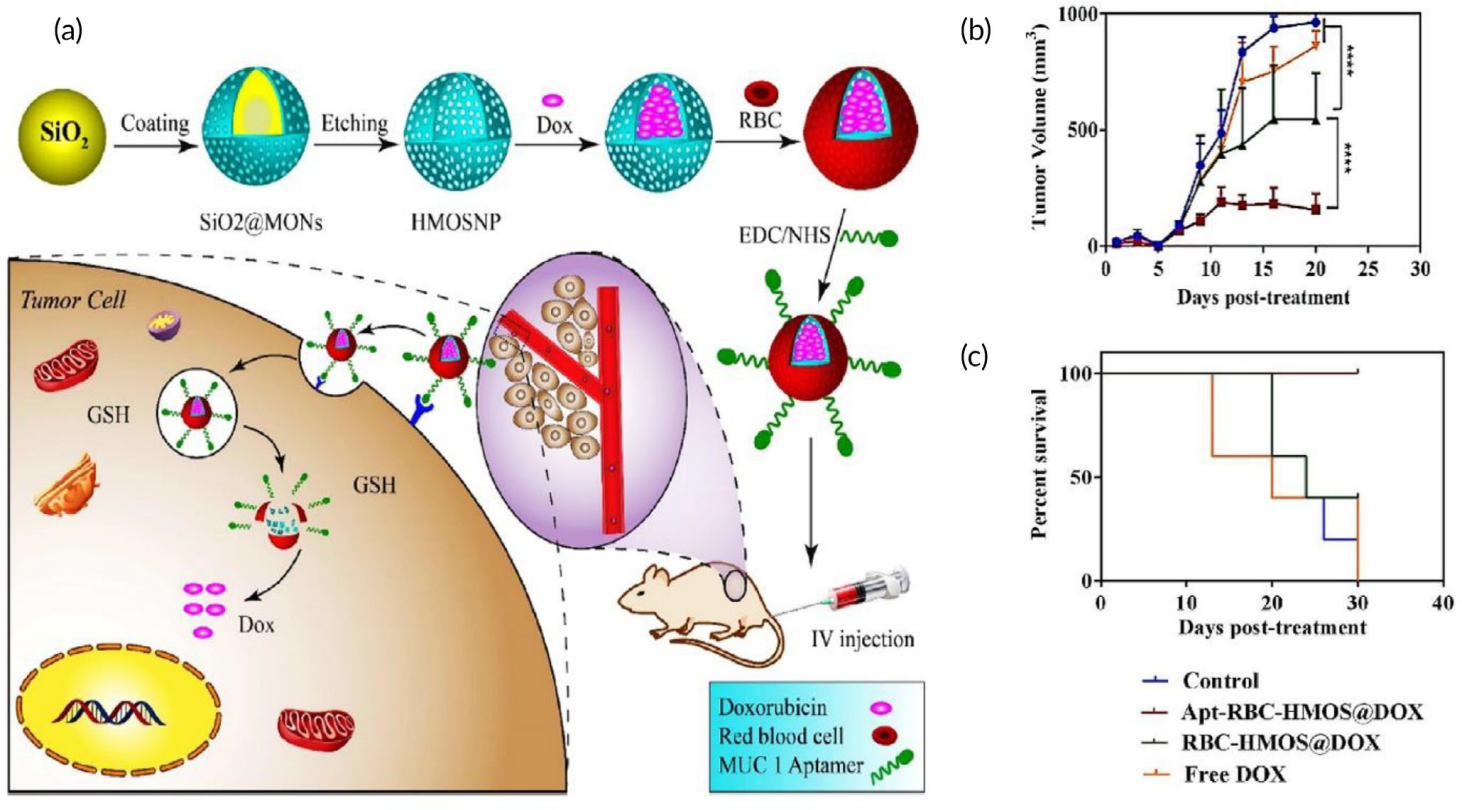
Red blood cell membrane‐coated biomimetic nanosystems for colorectal cancer‐targeted chemotherapy. (a) Preparation of Apt‐RBC‐HMOS@DOX for targeted chemotherapy of colorectal carcinoma by coating HMOS@DOX with red blood cell membranes, and subsequent functionalization with MUC‐1 aptamer. (b) Growth curves of tumor volume (mm^3^) after different treatments. (c) Overall survival rate of tumor‐bearing mice receiving different treatments (control, Apt‐RBC‐HMOS@DOX, RBC‐HMOS@DOX, and free doxorubicin). Reproduced with permission.^89^ Copyright 2022, Elsevier. Apt‐RBC‐HMOS@DOX, MUC‐1 aptamer‐surface functionalized RBC‐HMOS@DOX; DOX, doxorubicin; HMOS, hollow mesoporous organosilica nanoparticle; HMOS@DOX, DOX‐loaded HMOS; IV, intravenous; RBC‐HMOS@DOX; red blood cell membrane‐coated HMOS@DOX.

Biomimetic red blood cell membrane‐coated, doxorubicin‐loaded hollow mesoporous organosilica NPs (RBC‐HMOS@DOX) were recently developed for colorectal cancer‐targeted chemotherapy.^89^ To increase tumor accumulation and selective drug uptake by targeted colorectal cancer cells, the nanoassembly was functionalized with a DNA aptamer targeting the MUC‐1 receptor, which is overexpressed by tumor tissues (Apt‐RBC‐HMOS@DOX) (Figure 3a). In vivo studies revealed that, compared to free doxorubicin and RBC‐HMOS@DOX, the Apt‐RBC‐HMOS@DOX had enhanced tumor‐targeting ability, leading to a more significant suppression of tumor growth, with tumor sizes reduced to 20% of those in the control group. Additionally, the treatment resulted in increased survival rates in the treated mice (Figure 3b,c).^89^

**FIGURE 4:**
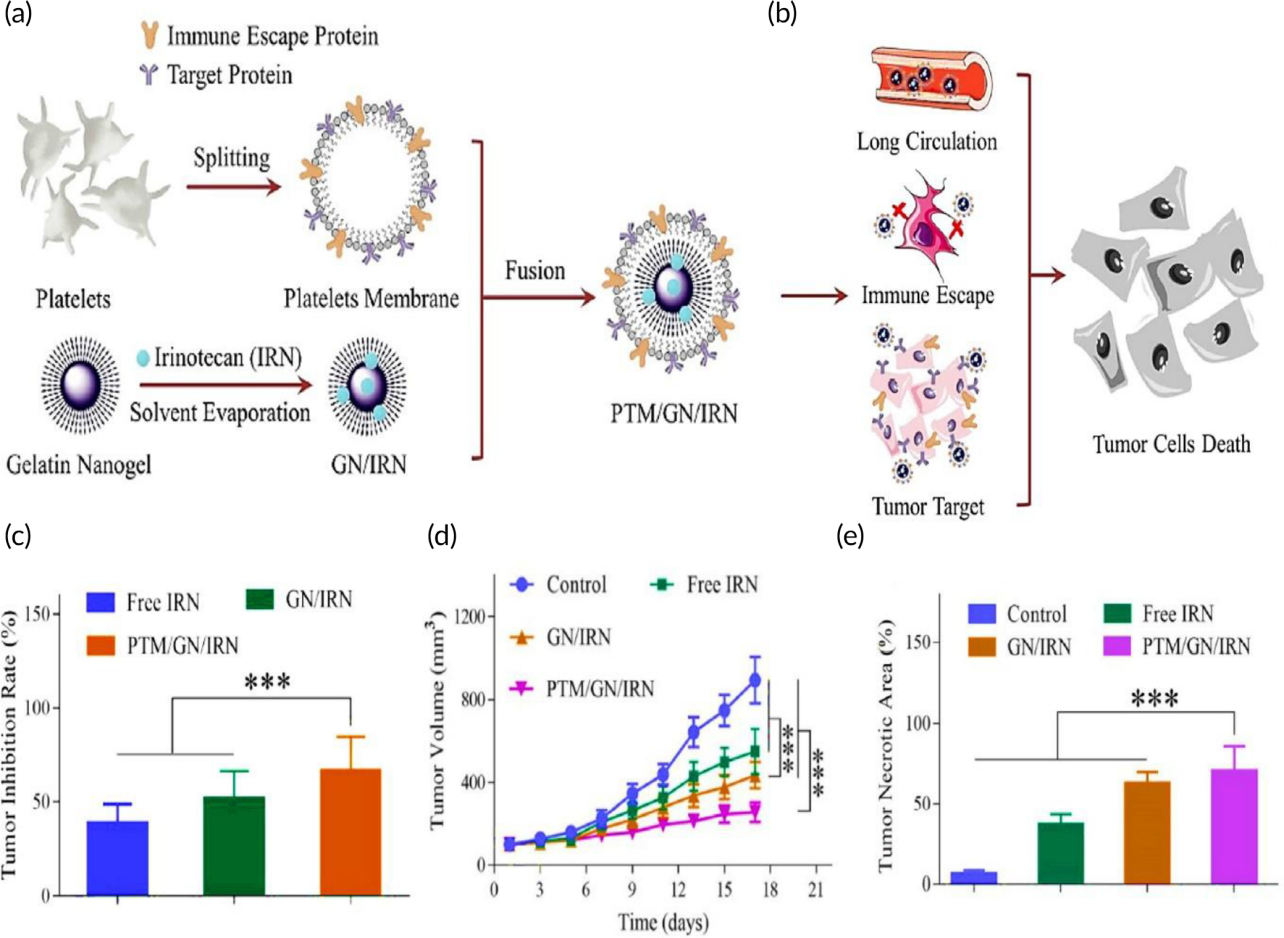
Biomimetic platelet membrane‐coated nanosystems for targeted chemotherapy of colorectal cancer. (a) Preparation of PTM/GN/IRN for targeted chemotherapy of colorectal carcinoma by coating GN/IRN with platelets membranes. (b) Illustration of the in vivo effects of the biomimetic nanosystem (superior blood circulation time, immune evasion, and tumor‐targeting ability) that culminates in tumor cell death. (c) Tumor inhibition rate (%) in tumor‐bearing mice receiving different treatments (free irinotecan, GN/IRN, and PTM/GN/IRN). (d) Growth curves of tumor volume after different treatments. € Evaluation of tumor necrotic area (%) in tumor‐bearing mice after different treatments. Reproduced with permission.^90^ Copyright 2019, Elsevier. GN, gelatin nanogel; GN/IRN, irinotecan‐loaded gelatin nanogel; IRN, irinotecan; PTM/GN/IRN, irinotecan‐loaded gelatin nanogel coated with platelets membranes.

Beyond those above‐mentioned, other chemotherapeutic drugs have been encapsulated in biomimetic nanosystems for colorectal cancer‐targeted chemotherapy. One example is irinotecan, a highly effective topoisomerase I inhibitor capable of impairing DNA transcription and replication. In this study, irinotecan was loaded in gelatin nanogels, and then the nanoassemblies were coated with platelets membranes (PTM/GN/IRN) (Figure 4a).^90^ Owing to the unique features of the platelet membrane coating (e.g., reduced uptake by macrophages, prolonged blood circulation, and intrinsic targeting ability to tumor sites and damaged vasculature), the designed PTM/GN/IRN exhibited reduced immune clearance, prolonged blood circulation, and superior tumor accumulation after intravenous injection into HT‐29 tumor models (Figure 4b). In vivo studies revealed that, compared to uncoated gelatin nanogels and free irinotecan, PTM/GN/IRN was more effective in suppressing tumor growth and inducing tumor cell apoptosis, displaying greater antitumor efficacy, with minimal systemic side effects (Figure 4c–e).^90^

**FIGURE 5:**
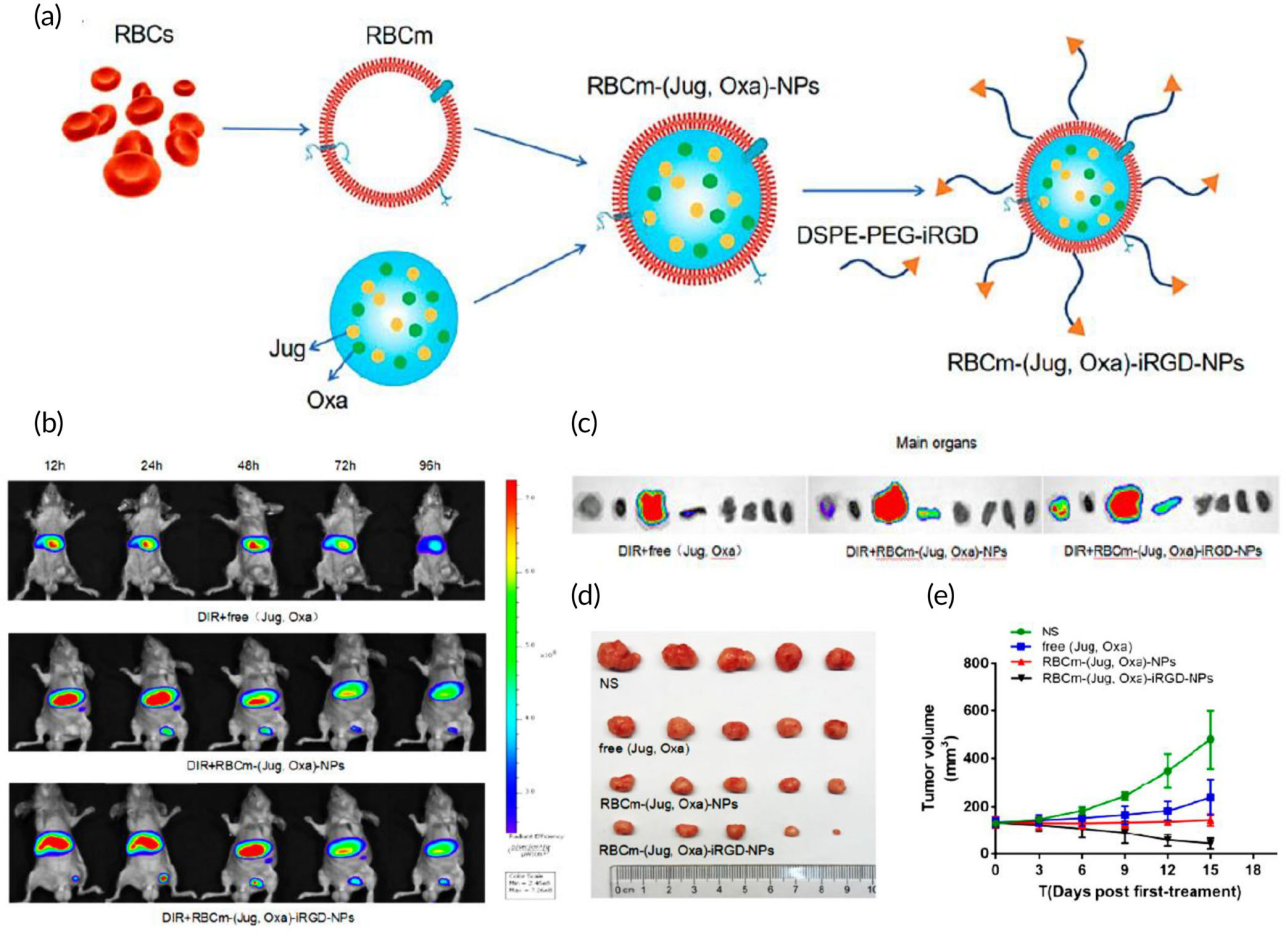
Red blood cell membrane‐coated nanosystems for improved chemotherapy of colorectal cancer. (a) Preparation of RBCm‐ (Jug, Oxa)‐iRGD‐NPs by coating Oxa and Jug co‐loaded nanostructures with iRGD‐modified red blood cell membranes. (b) In vivo distribution of free drugs and different treatments (iRGD‐modified and unmodified membrane‐coated NPs) 12, 24, 48, 72, and 96 h after intravenous injection into tumor‐bearing mice. (c) Fluorescence intensity of tumor and major organs (heart, liver, spleen, lungs, and kidney) 96 h after intravenous injection. (d) Photographs of tumor tissues after different treatments. (e) Growth curves of tumor volume in tumor‐bearing mice receiving different treatments. Reproduced with permission.^91^ Copyright 2022, SAGE Publications Ltd. Jug, juglone; NP, nanoparticle; Oxa, oxaliplatin; RBC, red blood cell; RBCm, red blood cell membrane; RBCm‐ (Jug, Oxa)‐iRGD‐NPs, iRGD‐modified red blood cell membrane‐coated nanoparticles co‐loaded with juglone and oxaliplatin; RBCm‐ (Jug, Oxa)‐NPs, red blood cell membrane‐coated nanoparticles co‐loaded with juglone and oxaliplatin.

Oxaliplatin, an effective compound that blocks DNA synthesis, is another chemotherapeutic drug with great therapeutical potential for colorectal cancer. However, the clinical application of oxaliplatin is still very challenging due to its short half‐life and severe side effects on normal organs (e.g., neurotoxicity).^91^ In a recent study, the antitumor effects of oxaliplatinwere studied in combination with juglone (a naturally occurring compound with well‐known antitumor effects). For this purpose, oxaliplatin and juglone were co‐loaded in a red blood cell membrane‐coated nanostructure modified with RGD internalizing peptide (iRGD, a tumor‐penetrating peptide), and its antitumor efficacy was demonstrated in vitro and in vivo after intravenous injection in colorectal cancer‐bearing mouse models (Figure 5a).^91^ The biomimetic nanosystem showed enhanced tumor‐targeting features, which augmented the accumulation of oxaliplatin and juglone at tumor tissues (Figure 5b,c). This improved antitumor efficiency and tumor eradication due to the synergistic effects of both compounds (Figure 5d,e). Furthermore, no serious organ damage and side effects were detected, highlighting the biosafety and biocompatibility of the nanosystem.^91^

Recently a novel formulation has been fabricated to investigate the combinational chemotherapy against colorectal cancer.^92^ Herein, hydroxymethyl phenylboronic acid (HPA)‐conjugated PLGA NPs, cloaked with mannose‐functionalized red blood cell membranes have been employed to co‐deliver chloroquine and artesunate (HPA/AS/CQ@Man‐EM).^92^ The results revealed significant potential of these nanocarriers for tumor proliferation suppression and impeding the tumor‐associated macrophages (TAMs) repolarization through targeting M2‐like TAMs and cancerous cells in both in vitro and in CT‐26 tumor‐bearing orthotopic mouse models.^92^

In another effort to develop biomimetic core‐shell nanomedicines for colorectal cancer‐targeted therapy, gambogic acid (a natural compound present in gamboge with remarkable antitumor effects on several types of cancer, namely in colorectal cancer), was loaded into PLGA NPs, and then coated with membranes derived from red blood cells.^93^, ^153^ In the in vitro studies, the resulting biomimetic nanosystem showed to be efficiently uptaken by SW480 colon cancer cells, inducing notorious antitumor effects and cell apoptosis. The antitumor efficacy of the nanoassembly was confirmed in vivo after intravenous injection into SW480 tumor‐bearing mouse models, as demonstrated by the greater inhibition of tumor growth and extended survival of mice. In summary, the designed nanostructure showed enhanced biocompatibility, stability, and anti‐opsonisation effects (owing to the red blood cell membrane cloaking), and demonstrated to improve the antitumor efficiency of gambogic acid.^93^ In a subsequent study aimed at increasing the tumor‐targeting ability for epidermal growth factor receptor (EGFR)‐expressing colorectal cancer, a recombinant protein (anti‐EGFR‐iRGD), harboring the EGFR antibody and the iRGD peptide, was functionalized on the surface of gambogic acid‐loaded PLGA NPs coated with red blood cell membranes.^94^ In vitro and in vivo studies showed enhanced tumor‐targeting features and superior uptake by cancer cells, inducing the greater suppression of tumor growth. Enhanced biocompatibility, stability, and safety were also achieved using this biomimetic nanostructure.^94^

**FIGURE 6:**
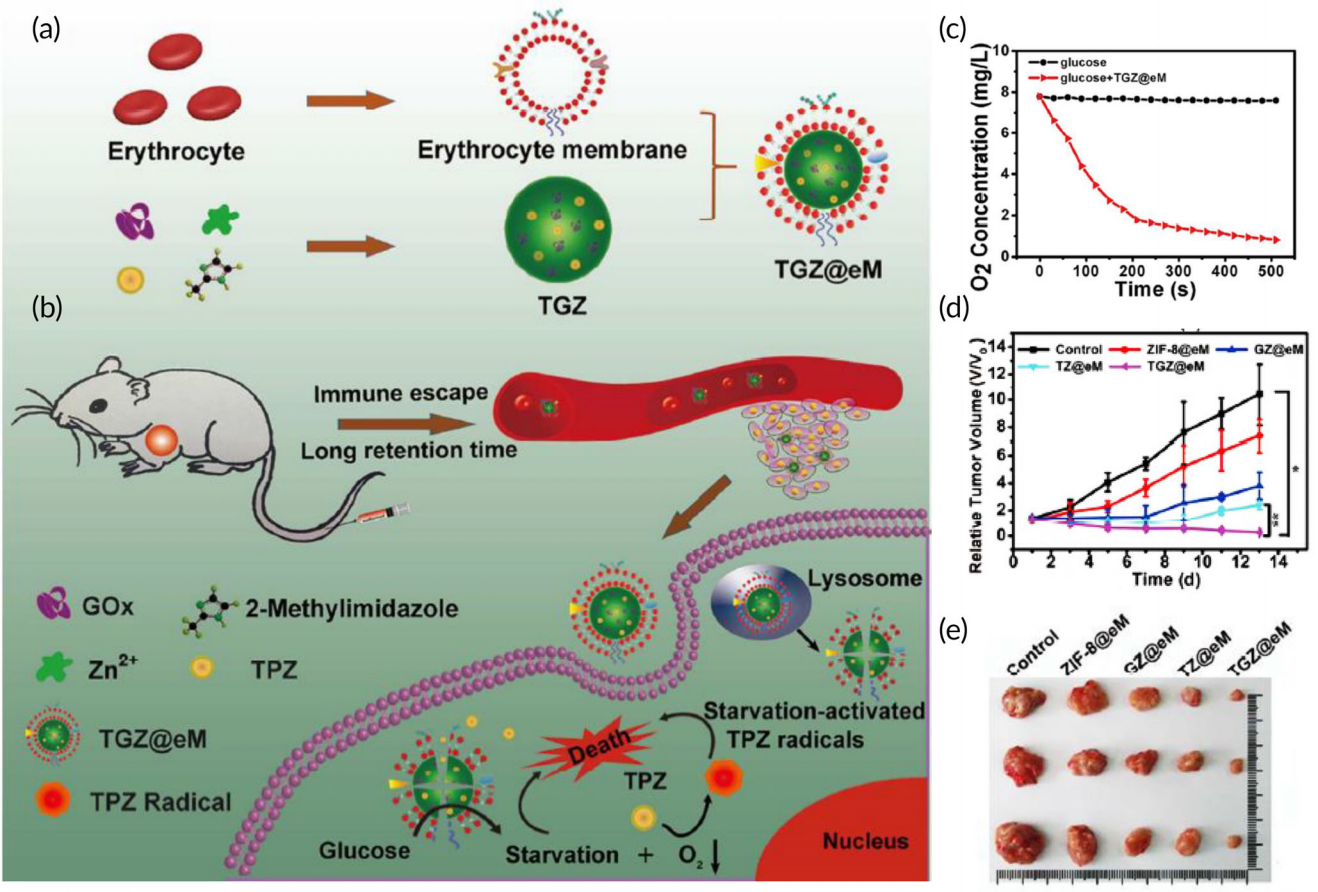
Red blood cell‐mimicking nanosystems for starvation‐activated therapy of colorectal cancer. (a) Illustration of the preparation of TGZ@eM for starvation‐activated colon cancer therapy by coating TGZs with red blood cell membranes. (b) Schematic illustration of the in vivo effects of TGZ@eM in inducing tumor cell death by starving tumor cells of glucose and converting the prodrug tirapazamine into cytotoxic radicals. (c) Assessment of the decrease in O_2_ concentration (mg/L) after addition of glucose to TGZ@eM solution. (d) Growth curves of tumor volume in tumor‐bearing mice receiving different treatments. (e) Photographs of tumor tissues after different treatments. Reproduced with permission.^95^ Copyright 2018, American Chemical Society. GOx, glucose oxidase; TGZ, zeolitic imidazolate framework‐8 NP co‐loaded with GOx and prodrug tirapazamine; TGZ@eM, red blood cell membrane‐coated TGZ; TPZ, tirapazamine.

Cancer starvation therapy has been recognized as an effective approach for cancer eradication. This strategy utilizes glucose oxidase (GOx) to consume O_2_ and glucose, which is a key nutrient for tumor growth, and convert it into gluconic acid and hydrogen peroxide (H_2_O_2_), thus starving tumor cells of glucose.^95^ Having this in mind, zeolitic imidazolate framework‐8 (ZIF‐8) NPs containing both GOx and prodrug tirapazamine were coated with a red blood cell membrane (TGZ@eM) for colon cancer starvation therapy (Figure 6a).^95^ TGZ@eM exhibited enhanced immune evasion and prolonged systemic circulation features, both attributed to the red blood cell membrane coating, which in turn contributed to increase the accumulation at tumor sites (Figure 6b). In summary, the resulting biomimetic nanosystem proved to be a promising strategy to enhance the efficacy of starvation‐activated colon cancer therapy in vivo due to its dual antitumor effects. TGZ@eM showed not only to retain the catalytic activity of GOx to starve tumor cells of glucose, but also to produce a hypoxic tumor microenvironment (due to the O_2_ consumption) required to convert the prodrug tirapazamine into cytotoxic radicals for enhanced tumor ablation (Figure 6c–e). The use of TGZ@eM demonstrated an improved tumor growth inhibition (TGI) rate of 97.6% in vivo compared to the untreated group. The tumor size in the TGZ@eM‐treated group was significantly smaller than that of the other groups.^95^

Ferroptosis, a cell death type featured by iron‐dependent lipid peroxidation and accumulation of ROS, has been exploited for colorectal cancer‐targeted therapy.^96^ Resveratrol has proven to be an effective ferroptosis‐inducer by increasing ROS intratumoral accumulation and inducing lipid peroxidation of colorectal cancer cells. This ferroptosis‐inducing anticancer compound can downregulate the expression of the channel protein solute carrier family 7 member 11 (SLC7A11) and glutathione peroxidase 4 (GPX4). Have this in mind, recently, resveratrol‐loaded poly(ε‐caprolactone)‐poly(ethylene glycol) (PCL‐PEG) NPs were coated by red blood cell membranes.^96^ Coated NPs showed extended half‐life, immune evasion, and superior tumor‐targeting features (that increased when coupled to the tumor‐penetrating peptide iRGD). Hence, the nanosystem showed to increase targeted bioaccumulation at targeted colorectal tumors in vivo for effective ferroptosis‐targeted therapy.^96^

Photothermal cancer therapy, a type of cancer phototherapy, is a strategy that utilizes photothermal agents capable of absorbing NIR light and converts it into cytotoxic heat for thermal ablation of tumor cells.^154^ Recent efforts have investigated the potential of biomimetic nanosystems to improve the efficacy of PTT, with one study taking advantage of the NIR‐absorption capacity of metal bismuth NPs to develop a cancer cell‐mimicking nanosystem for targeted PTT of colorectal cancer.^97^ The biomimetic nanostructure (prepared by coating the metal bismuth NP inner core with a CT26 colon cancer cell membrane) showed superior biocompatibility, prolonged blood circulation, and homotypic tumor‐homing features in vivo, resulting in superior accumulation at homotypic CT26 colon cancer cells when compared to non‐coated counterparts. Furthermore, upon NIR irradiation, the nanosystem showed pronounced photothermal and antitumor effects, as demonstrated by the efficient hyperthermia‐induced ablation of CT26 colon cancer cells 12 days after treatment.^97^

In another different study, superparamagnetic nanoclusters were loaded with cypate (a photothermal agent with NIR‐absorption capability), and then coated with a red blood cell membrane.^98^ The NIR fluorescence ability of cypate and the MRI ability of superparamagnetic nanoclusters enabled the in vivo tracking of the biomimetic nanosystem, which showed remarkable tumor‐targeting features and tumor‐specific accumulation compared to non‐coated counterparts. After 808 nm laser irradiation, the biomimetic nanosystem could induce efficient hyperthermia‐induced ablation of HCT‐116 cells due to the NIR‐absorption ability and the photothermal effects of cypate. In summary, this study provided a biomimetic nanosystem for bimodal NIR fluorescence and MRI‐guided PTT of colorectal cancer.^98^

In addition, the combination of PTT with other anticancer approaches, such as cancer immunotherapy and chemotherapy has also been investigated to achieve better antitumor efficacy compared to respective monotherapies.^99^, ^100^ In an effort to develop a combinational PTT‐immunotherapy approach against primary and metastatic colorectal cancer, hollow gold nanocage nanocomposites loaded with galunisertib (a transforming growth factor‐β [TGF‐β] inhibitor) were coated with a macrophage membrane functionalized with the monoclonal antibody against programmed cell death ligand 1 (anti‐PDL1 antibody, an immune checkpoint inhibitor).^99^ The nanoassembly showed enhanced tumor‐homing features and superior accumulation at targeted colon cancer cells, displaying remarkable photothermal effects under NIR irradiation with consequent release of tumor‐specific antigens. This could trigger potent antitumor immune responses and induce notorious antitumor effects in combination with anti‐PDL1 antibody and galunisertib. Hence, this study provides a synergistic approach for effective eradication of colon cancer through the double combination of photothermal cancer therapy and immunotherapy.^99^

In a different study aimed at combining cancer chemotherapy with imaging‐guided PTT, Zn_1.25_ Ga_1.5_Ge_0.25_ O_4_:Cr^3+^, Yb^3+^, Er^3+^ (ZGGO) persistent luminescence NPs were coated with mesoporous silica, and co‐loaded with IR825 (a fluorescent photothermal dye) and irinotecan.^100^ The nanosystem was then cloaked with a CT26 colon cancer cell‐macrophage hybrid membrane to produce a core‐shell nanostructure (IR825/Ir ZGGO@SiO_2_@CMM). The nanosystem could preserve the homologous tumor‐targeting ability of tumor cells and the immune escape ability of macrophages. This resulted in prolonged half‐life and superior tumor accumulation in vivo, as demonstrated by the superior luminescence intensity in tumor tissues. In summary, IR825/Ir ZGGO@SiO_2_@CMM showed to combine cancer chemotherapy with imaging‐guided PTT to produce remarkable antitumor effects in vivo.^100^

**FIGURE 7:**
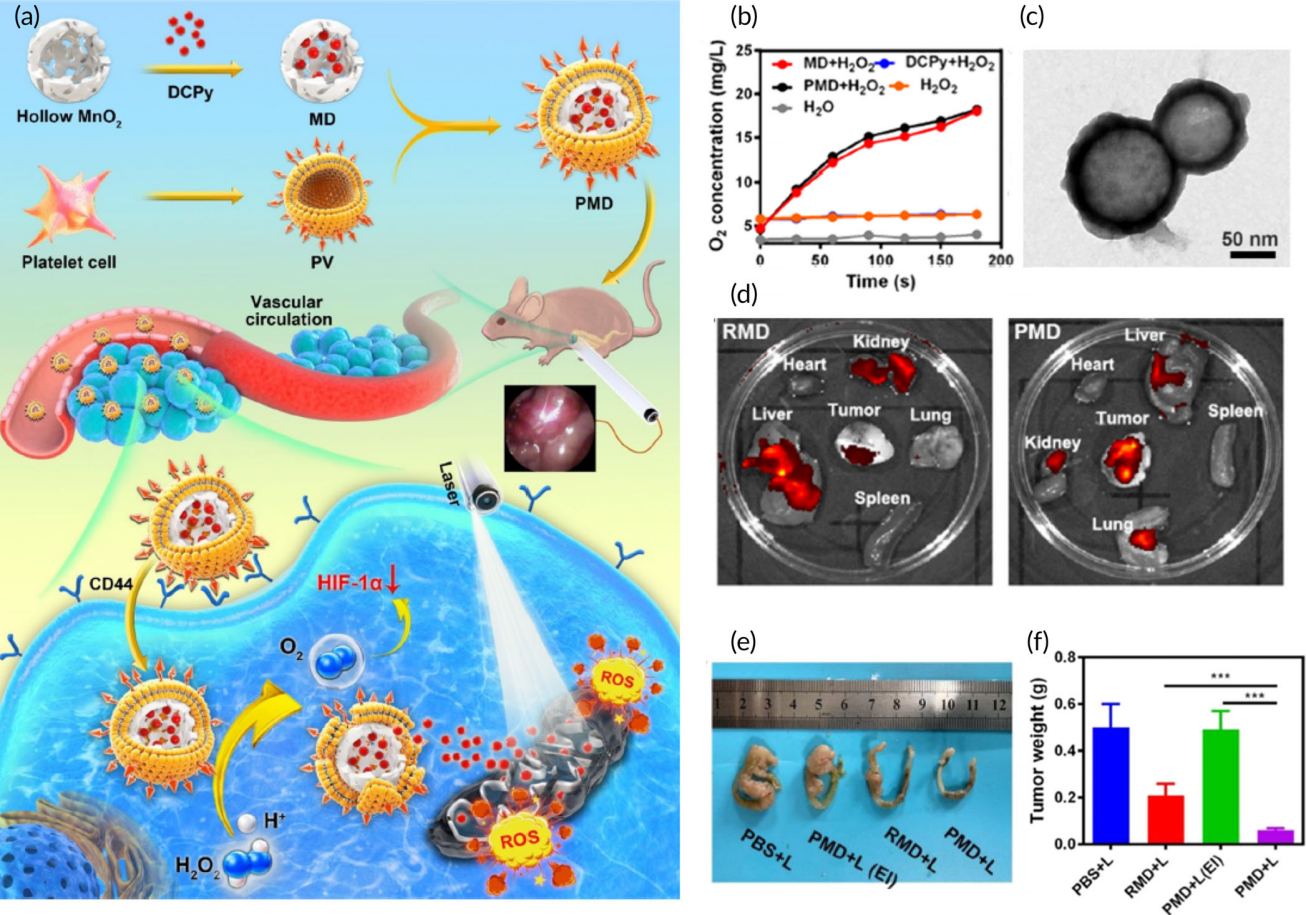
Platelet membrane‐mimicking nanosystems for colorectal cancer‐targeted PDT. (a) Depicting of PMD preparation by coating MD cores with platelet membranes, and illustration of their in vivo molecular effects. (b) The O_2_ generating capacity of different treatments (DCPy, MD, and PMD) in combination with H_2_O_2_. (c) TEM images of the core‐shell nanostructure of PMD. (d) Comparison of tumor accumulation between MD coated with red blood cells (left) and platelet membranes (right). (e) Photographs of tumor tissues after different treatments. (f) Tumor weight changes after different treatments. Reproduced with permission.^101^ Copyright 2022, American Chemical Society. DCPy, 6‐dichloropyridine; MD, DCPy‐loaded MnO_2_ NP; PDT, photodynamic therapy; PMD, platelet membrane‐coated MD; PMD + L (EI), PMD plus external laser irradiation; PMD + L, PMD plus abdominal laser irradiation; PV, platelet membrane‐derived nanovesicle; RMD, red blood cell membrane‐coated MD; TEM, transmission electron microscopy.

Photodynamic therapy (PDT) is another type of cancer phototherapy. This light‐activated strategy employs photosensitizers with light‐absorbing features capable of converting surrounding O_2_ into cytotoxic ROS (^1^O_2_) upon NIR light irradiation.^85^ PDT effectiveness is severely hampered by the hypoxic tumor microenvironment of solid tumors (due to the lack of oxygen) and by the reduced penetration of laser irradiation into deep tumorous tissues. Photosensitizers with aggregation‐induced emission (AIE) properties have been studied for colorectal cancer‐targeted PDT. In a recent study, 2,6‐dichloropyridine (DCPy), an AIE photosensitizer with NIR‐absorption and ROS‐generation abilities, was loaded in hollow MnO_2_ NPs (named MD). These NPs were able to decompose hydrogen peroxide (H_2_O_2_) into H_2_O and O_2_ within the acidic tumor microenvironment, synergistically surpassing intra‐tumoral hypoxia and increasing PDT efficacy (Figure 7a,b).^101^ To increase tumor‐targeted accumulation, MD was further coated with platelet membranes (Figure 7c). The coated NPs (PMD) showed efficient tumor‐targeting and accumulation, due to the interaction of P‐selectin overexpressed on platelets to the cancer cell surface‐expressed CD44 (Figure 7d). Due to the inability of laser irradiation to penetrate deep in the abdominal cavity‐located colorectal cancers, an optical fiber was placed into the mice abdominal cavity to augment PDT efficacy via laser irradiation. The combination of PMD and abdominal laser irradiation was more effective in inducing tumor cytotoxicity compared to PMD plus external laser irradiation (Figure 7e,f). Hence, this strategy could overcome concerns related to the low penetration of laser irradiation into abdominally located cancers, increasing PDT efficiency against deep colorectal cancers.^101^

Sonodynamic therapy (SDT), a non‐invasive strategy for cancer therapy, shares the same anticancer mechanisms as PDT, while overcoming its main limitation: the limited penetration of laser irradiation into deep tumorous tissues that reduces PDT efficiency.^155^, ^156^ SDT uses low‐intensity ultrasound (US) that activate sonosensitizers to convert surrounding O_2_ into cytotoxic ROS (^1^O_2_) to destroy cancer cells (similar to photosensitizes).^157^ In opposite to NIR‐triggered PDT, the strong deep penetration of US makes SDT a more promising therapeutic strategy for deep‐located cancers.^155^, ^156^ In a recent effort to develop a combinatorial PTT‐SDT approach against colorectal cancer, hollow Cu_9_S_8_ nanospheres—obtained by sulfurization of cuprous oxide (Cu_2_O) NPs—were loaded with hemoporfin, and then coated by CT26 colon cancer cell membranes, to simultaneously achieve the goals of enhanced biocompatibility, systemic circulation half‐life and homotypic tumor‐targeting.^102^ Compared to non‐coated NPs, an increase of 6.47‐fold and 1.94‐fold (in blood circulation time and tumor accumulation, respectively) was reported into biomembrane‐surface engineered NPs‐treated mice models. While hollow Cu_9_S_8_ nanospheres acted as photothermal agents for hyperthermia‐induced tumor ablation via PTT upon NIR light irradiation, loaded hemoporfin acted as a sonosensitizer generating cytotoxic ^1^O_2_ for SDT‐mediated tumor cell death under US irradiation. In summary, this study provides a NIR/US‐responsive colon cancer cell membrane‐coated, sonosensitizer‐loaded photothermal nanosystem for synergistic colon cancer‐targeted PTT‐SDT.^102^ Similarly, a cascade nanoreactor comprised of HCT‐116 cancer cell membrane‐camouflaged platinum nanozymes decorated hollow polydopamine NPs, co‐encapsulating chloroquine and chlorin e6 (Ce6, a sonosensitizer), named CCP@HP@M, has been employed to investigate their colon cancer‐fighting efficacy via SDT strategy.^103^ The in vitro and in vivo SDT therapy demonstrated increased tumor site accumulation of CCP@HP@M leading to hypoxia attenuation, enhanced ROS generation, apoptosis and ferroptosis induction, and autophagy blockade. Together, these results introduce a novel nanoreactor to be employed for increased cancer‐eradicating properties of SDT.^103^

**FIGURE 8:**
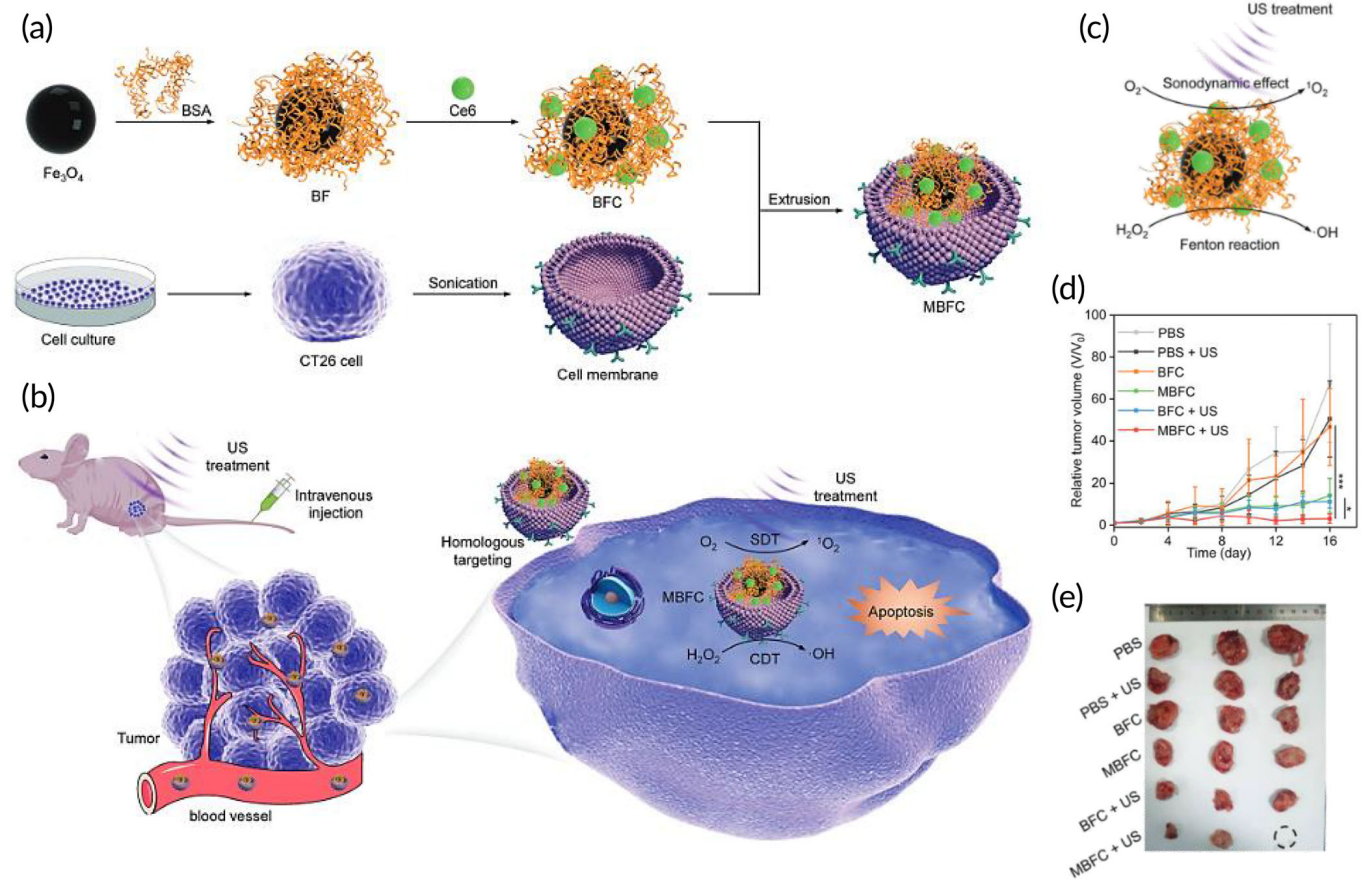
Colon cancer cell membrane‐coated, sonosensitizer‐loaded chemodynamic agent nanosystems for synergistic colon cancer‐targeted CDT‐SDT. (a) Depicting of MBFC preparation by coating BFC cores with CT26 colon cancer cell membranes via co‐extrusion. (b) In vivo homologous‐tumor targeting ability of MBFC after intravenous injection, and its chemodynamic and sonodynamic effects upon US irradiation. (c) Illustration of Ce6‐mediated SDT upon US irradiation and Fe_3_O_4_ NP‐mediated CDT based on Fenton reaction. (d) Tumor volume curves after receiving different treatments. (e) Photographs of tumor tissues after different treatments. Reproduced with permission.^104^ Copyright 2022, Royal Society of Chemistry. BF, BSA‐modified Fe_3_O_4_ NP; BFC, Ce6‐loaded BF; BSA, bovine serum albumin; CDT, chemodynamic therapy; Ce6, chlorin e6; Fe_3_O_4_ NP, iron oxide nanoparticle; MBFC, CT26 colon cancer cell membrane‐coated BFC; SDT, sonodynamic therapy; US, ultrasound.

Chemodynamic therapy (CDT) is an anticancer approach that relies on the ability of chemodynamic agents (e.g., iron‐based materials) to convert the surrounding hydrogen peroxide (H_2_O_2_) molecules into cytotoxic hydroxyl radicals (•OH) based on Fenton‐like reactions.^104^, ^158^ Thus, the combination of CDT and SDT may result in synergistic effects by enhancing ROS intratumoral production. To yield a CDT‐SDT combinatorial therapy against colorectal cancer, a biomembrane‐coated nanosystem was produced.^104^ The biomimetic NPs were composed of bovine serum albumin (BSA)‐modified iron oxide (Fe_3_O_4_) NPs (as carriers and chemodynamic agents), which were loaded with Ce6 (a sonosensitizer), to yield BFC NPs, and then surface‐coated by CT26 colon cancer cell membranes (MBFC), to achieve enhanced systemic circulation time and homotypic tumor‐targeting (Figure 8a).^104^ Under US irradiation, Ce6 could produce a large amount of cytotoxic ^1^O_2_ for SDT‐mediated tumor cell death, which was amplified by •OH production from Fe_3_O_4_ NP‐mediated CDT (Figure 8b,c). A tumor suppression efficacy of 83.3% was reported in the MBFC plus US irradiation treatment group, which was superior to those treated with both non‐coated NPs (BFC) plus US irradiation (65.1%) and MBFC only (57.7%). Hence, due to its chemodynamic and sonodynamic combinatorial effects, the biomimetic nanosystem showed efficient tumor‐killing activities in vitro, and could efficiently suppress colorectal tumors growth in vivo (Figure 8d,e).^104^

Apart from single cell membranes, hybrid cell membranes have also been studied as coating materials. Distinct cell membrane types can be mixed and hybridized (by techniques such as extrusion and sonication) to yield multifunctional and multifaceted hybrid cell membranes that incorporate the biofunctionalities of each cell membrane type.^74^ In one study, hybrid cell membranes (obtained by fusing the membranes of neutrophils and red blood cells) were developed and after surface modification with palmitoyl ascorbate, they were used to coat Ce6‐loaded ZIF‐8 NPs.^105^ After reaching the tumor site, sonosensitizer Ce6 could produce cytotoxic ROS under US for SDT‐mediated tumor cell death. While neutrophil membranes could inhibit neutrophils chemotaxis and intratumoral infiltration, and suppress M1‐to‐M2 macrophage polarization to reprogram the immunosuppressive tumor microenvironment, enzymes on the red blood cell membrane (e.g., catalase) could decompose intratumoral H_2_O_2_ (produced by palmitoyl ascorbate) to generate O_2_, thereby reducing tumoral hypoxia and enhancing Ce6‐mediated SDT efficacy. This way, in this study a hybrid cell membrane‐coated nanosystem with O_2_ self‐generation properties was designed for synergistic reprogramming of the immunosuppressive tumor microenvironment and colorectal cancer‐targeted SDT.^105^

Gene therapy has also been applied for colon cancer therapy. MicroRNAs (miRNAs) have an important role in tumor tissues.^107^ They can act as post‐transcriptional silencers, by inhibiting the translation of target messenger RNAs (mRNAs). In vivo intracellular delivery of miRNA faces some hindering challenges, including rapid degradation by RNase, inefficient and non‐targeted delivery, which compromises biological activity, and unwanted off‐target effects. To address these shortcomings, biomembrane‐surface engineered NPs have been investigated as nanocarrier delivery systems for in vivo miRNA delivery.^106^, ^107^

miR190 is a promising anticancer approach against colorectal cancer. miR190 has been shown to downregulate the vascular endothelial growth factor (VEGF) signaling pathway, which is involved in angiogenesis (endothelial cell proliferation and blood vessel formation) and tumor progression. In addition, miR190 has been to suppress Smad2/4, that are transcription factors in the TGF‐β signaling pathway. TGF‐β signaling pathway is involved in tumor resistance to chemotherapeutic drugs and therapeutic failure.^106^ Thus, by inhibiting both the VEGF and TGF‐β/Smad signaling pathways, miR‐190 has the potential to reduce VEGF expression and angiogenesis, increase tumor sensitivity to anticancer drugs, and reduce chemoresistance, respectively.^106^ Recently, PLGA‐b‐PEG DC‐cho NPs co‐loading doxorubicin and miR‐190‐Cy7, were coated by HCT‐116 colon cancer cell membranes for simultaneous colorectal cancer diagnostic imaging, chemotherapy, and gene therapy (theranostics).^106^ In vivo fluorescence imaging of the miR‐190‐conjugated cyanine 7 (Cy7, a NIR dye) was used for in vivo tracking of the nanosystem, which showed remarkable tumor‐targeting features. miR‐190 efficiently inhibited tumor angiogenesis and growth, and suppressed TGF‐β signaling in colorectal cancer cell lines and mouse models, enhancing tumor sensitivity to doxorubicin. Twenty‐two days after in vivo administration, biomembrane‐coated, co‐loaded NPs decreased tumor volume by 75% and VEGF expression by 80%.^106^

**FIGURE 9:**
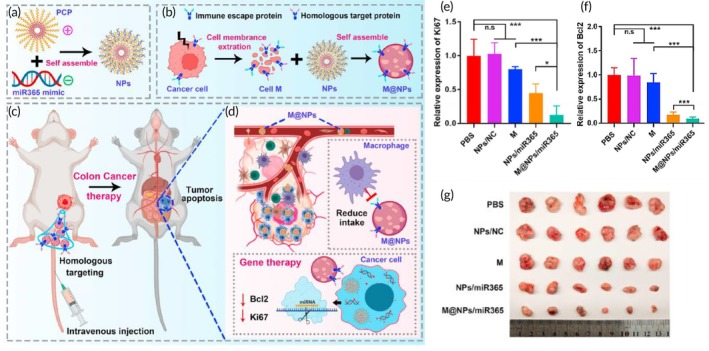
Colon cancer cell membrane‐mimicking nanosystems as gene delivery systems for colorectal cancer‐targeted therapy. (a) Depicting of the preparation of miR365‐loaded poly (citrate‐peptide) nanoparticles. (b) Coating the loaded nanoparticles with MC38 colon cancer cell membranes for immune escape and homologous tumor‐targeting. (c) Intravenous injection into MC38 tumor‐bearing mice model and specific tumor accumulation. (d) M@NPs/miR365 molecular mechanisms for targeted gene therapy. (e) Relative expression of Ki67 fluorescence. (f) Relative expression of Bcl2 fluorescence. (g) Photographs of tumor tissues after different treatments. Reproduced with permission.^107^ Copyright 2022, Elsevier. M@NPs/miR365 (also named M@NPs), NPs/miR365 coated with MC38 colon cancer cell membranes; NPs/miR365 (also named NPs), PCPs loaded with miR365; PCPs, ε‐poly‐L‐lysine polypeptide‐functionalized poly (citrate‐peptide) nanoparticles.

miR365 has been recognized as a prominent anticancer target, as it can act as tumor suppression gene by downregulating the expression of Bcl2 and Ki67. This can induce tumor cell apoptosis and inhibit cell proliferation. Recently, poly(citrate‐peptide) NPs were used as carriers to deliver miR365 to target MC38 colon cancer cells for efficient cancer therapy.^107^ To reduce macrophage immune clearance and increase its homologous tumor‐targeting ability, the nanosystem was decorated with MC38 colon cancer cell membranes (M@NPs/miR365) (Figure 9a–c). In the in vivo studies, M@NPs/miR365 showed to efficiently inhibit the Ki67/Bcl2 expression for superior apoptosis of MC38 cancer cells (Figure 9d–f). Overall, this gene delivery nanosystem showed great promise to suppress colon cancer progression and proliferation via regulation of gene expression (Figure 9g).^107^

Radiotherapy uses x‐ray irradiation to destroy cancer cells. This is a widely employed anticancer approach for treating nearly 70% of cancer patients. Nevertheless, some factors hinder its clinical success, such as (1) high tumor resistance to radiation; (2) severe side effects including tissue fibrosis and induction of later‐onset cancers; and (3) reliance on the tumor oxygen levels, and thus tumor hypoxia (a hallmark of solid tumors) can compromise the efficacy of radiotherapy.^108^ With this in mind, improving tumor radiosensitivity is critical to increase radiotherapy anticancer efficacy. Recently, platinum (Pt)‐integrated hollow polydopamine NPs co‐loading apoptin (a radiosensitizer) and verteporfin (a photosensitizer), were camouflaged by HCT‐116 colon cancer cell membrane to obtain a biomimetic nanosystem for colon cancer‐targeted radiotherapy.^108^ The membrane coating endowed the nanosystem with homotypic‐tumor targeting and tumor‐specific accumulation features, and its pH‐responsive properties enabled drug release in the poor acidic conditions of the tumor microenvironment. Under x‐ray irradiation, tumor radiosensitivity was greatly improved by three strategies: (1) tumor hypoxia relief via the catalase‐like activity of Pt NPs, which can convert endogenous H_2_O_2_ into O_2_; (2) direct tumor apoptosis induction via radiosensitizer apoptin; and (3) x‐ray‐induced PDT (X‐PDT) via photosensitizer verteporfin, which can absorb x‐ray irradiation and generate cytotoxic ^1^O_2_ for improved PDT efficacy and radiosensitivity. Superior antitumor effects and tumor cell apoptosis in vivo were achieved.^108^

**FIGURE 10:**
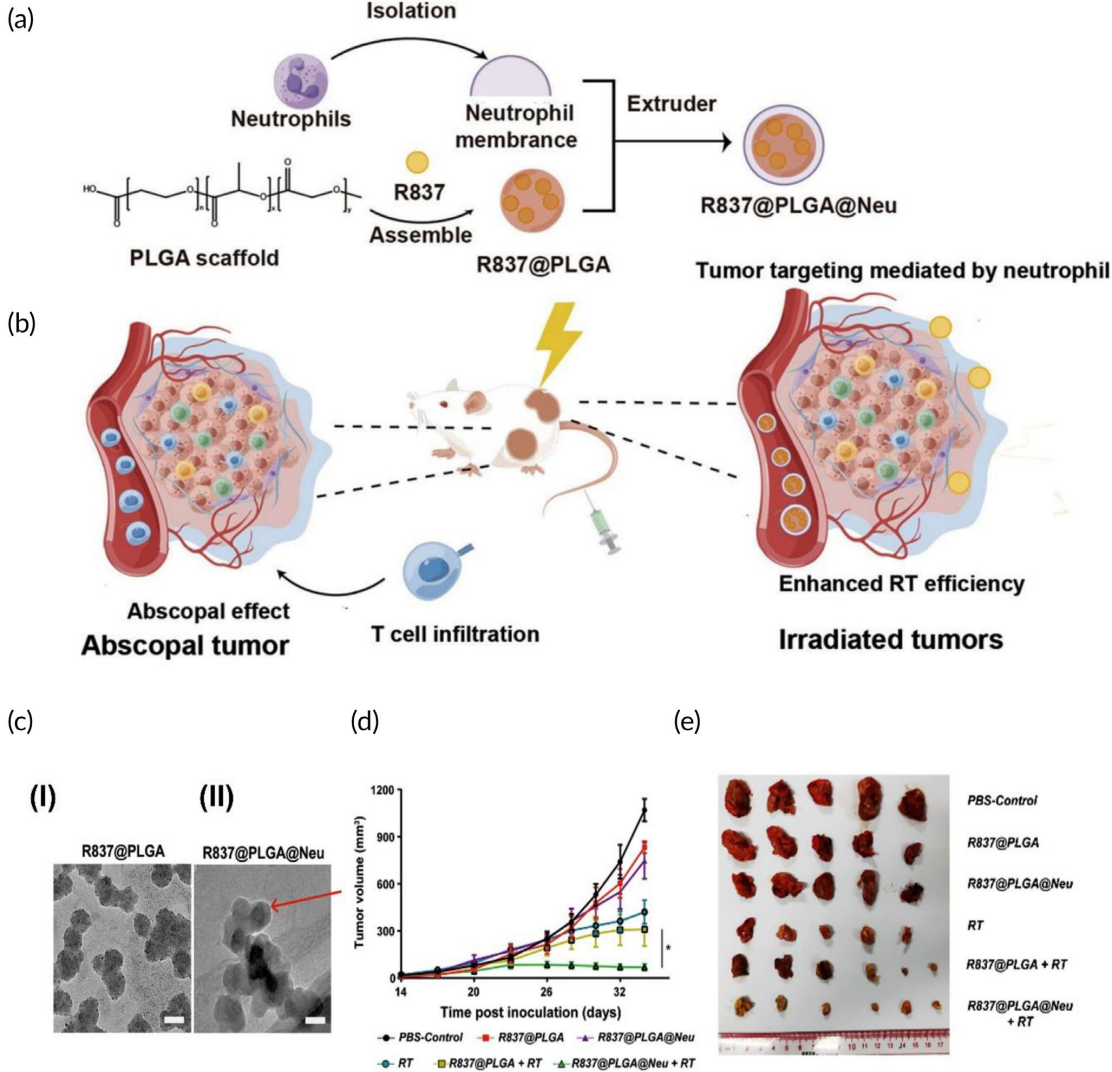
Neutrophil membrane‐coated nanosystems for colorectal cancer therapy via combinatorial radiotherapy and immunotherapy. (a) Schematic illustration of R837@PLGA@Neu NPs preparation by co‐extruding R837@PLGA NPs and extracted neutrophil membranes through porous membranes. (b) The tumor‐targeting ability of R837@PLGA@Neu NPs after in vivo administration (mediated by neutrophil membranes), and their antitumor effects not only at radiation‐treated tumors, but also at distant non‐irradiated tumors through T cell activation (abscopal effect). (c) TEM images of (I) R837@PLGA NPs, and (II) R837@PLGA@Neu NPs (red arrow indicates membrane coating). (d) Tumor volume (mm^3^) curves after different treatments. (e) Photographs of tumor tissues after different treatments. Reproduced with permission.^109^ Copyright 2023, Springer Science + Business Media. PLGA, poly (lactic‐*co*‐glycolic acid) nanoparticle; R837, imiquimod, a toll‐like receptor 7 (TLR 7) agonist; R837@PLGA, R837‐loaded PLGA; R837@PLGA@Neu, neutrophil membrane‐coated R837@PLGA; RT, radiation therapy (or radiotherapy).

Radiotherapy, by its own, can induce immune responses that stimulate neutrophils accumulation at the irradiated tumor tissues via release of proinflammatory cytokines. When combined with immunotherapy strategies, such as R837 (imiquimod, a toll‐like receptor 7 [TLR 7] agonist), the antitumor potential can be greatly enhanced and extended to other non‐irradiated tumor areas (called abscopal effect).^109^ R837 can significantly impact the tumor microenvironment immunity by inducing the maturation of dendritic cells and infiltration of CD8^+^ T cells at tumors, thus stimulating local and systemic antitumor immune responses. To enhance the radiation therapy efficacy against colorectal cancer and potentiate the abscopal effect, PLGA NPs loading R837 (R837@PLGA NPs) were coated with neutrophil membranes to produce biomimetic core‐shell NPs (R837@PLGA@Neu NPs) (Figure 10a,c).^109^ Following in vivo administration and after radiation therapy, R837@PLGA@Neu NPs showed specific‐tumor targeting and accurate drug delivery, as neutrophils are assumed to migrate to tumors prior to site‐specific irradiation. R837 could induce efficient antitumor immunity at locally treated tumors, and the subsequent T cell infiltration and activation produced systemic immune responses in distant tumors (Figure 10b). The experimental studies showed that the combination of R837@PLGA@Neu NPs with radiation therapy produced significant antitumor efficacy at both locally irradiated tumors and distant non‐irradiated tumors through the abscopal effect, as demonstrated by the TGI of 97.61% at the local tumors and 40.29% at the abscopal tumors, along with a notable decrease in tumor volume and size (Figure 10d,e).^109^

### 6.2 Gastric cancer

Gastric (or stomach) cancer is a prevalent subtype of gastrointestinal cancer and is the fourth major cause of cancer‐related deaths worldwide.^132^, ^159^, ^160^, ^161^ The most preferred therapeutic approaches for gastric cancer are surgical resection of the tumor, and chemotherapy. However, in most cases, surgery cannot be employed, since most gastric cancer patients are usually asymptomatic at early stages.^162^ In such cases, the palliative benefits of chemotherapy have been demonstrated, with the primary goal of improving quality of life and survival.^110^, ^132^ The development of chemoresistance and serious adverse effects of the commonly used chemotherapeutic drugs suggest the need of effective therapies with minimal toxicity.

Having this in mind, a biomimetic human cytotoxic CD8^+^ T lymphocyte (hCTL) membrane‐based nanosystem was developed for gastric cancer treatment. In this study, paclitaxel (a common chemotherapeutic drug used in gastric cancer treatment) was loaded into PLGA NPs, and then coated with membranes derived from hCTLs.^110^ The nanoassemblies (TPNPs) showed reduced immune uptake by macrophages, prolonged blood circulation, and superior tumor‐specific accumulation. This was attributed to the presence of adhesion molecules on hCTL membranes that could interact with specific tumor‐expressing ligands. In order to increase tumor‐targeting, TPNPs were combined with local low‐dose irradiation, which was shown to augment the expression of adhesion molecules (e.g., ICAM‐1) on tumor vasculature. In vivo studies revealed an increased accumulation of TTNPs at tumor sites in mice co‐treated with TTNPs and local low‐dose irradiation, when compared to non‐irradiated mice. This resulted in superior antitumor effects and greater suppression of tumor growth.^110^

Although considered the first‐line chemotherapeutic approach for gastric cancer, paclitaxel‐based monotherapies still lack clinical efficacy.^111^ To overcome the issues related to paclitaxel‐associated cancer cell resistance, a combinatorial strategy combining paclitaxel with triptolide (a naturally occurring active compound with proven antitumor effects, capable of enhancing the antitumor efficacy of paclitaxel) was investigated.^111^ In this study, red blood cell membrane‐coated PLGA NPs were selected as nanocarriers to co‐deliver both drugs to targeted tumor sites, simultaneously achieving the goals of immune evasion and prolonged blood circulation. The double combination of paclitaxel and triptolide produced remarkable cytotoxic effects on gastric cancer cells in vitro, due to the synergistic effects of both drugs.^111^

**FIGURE 11:**
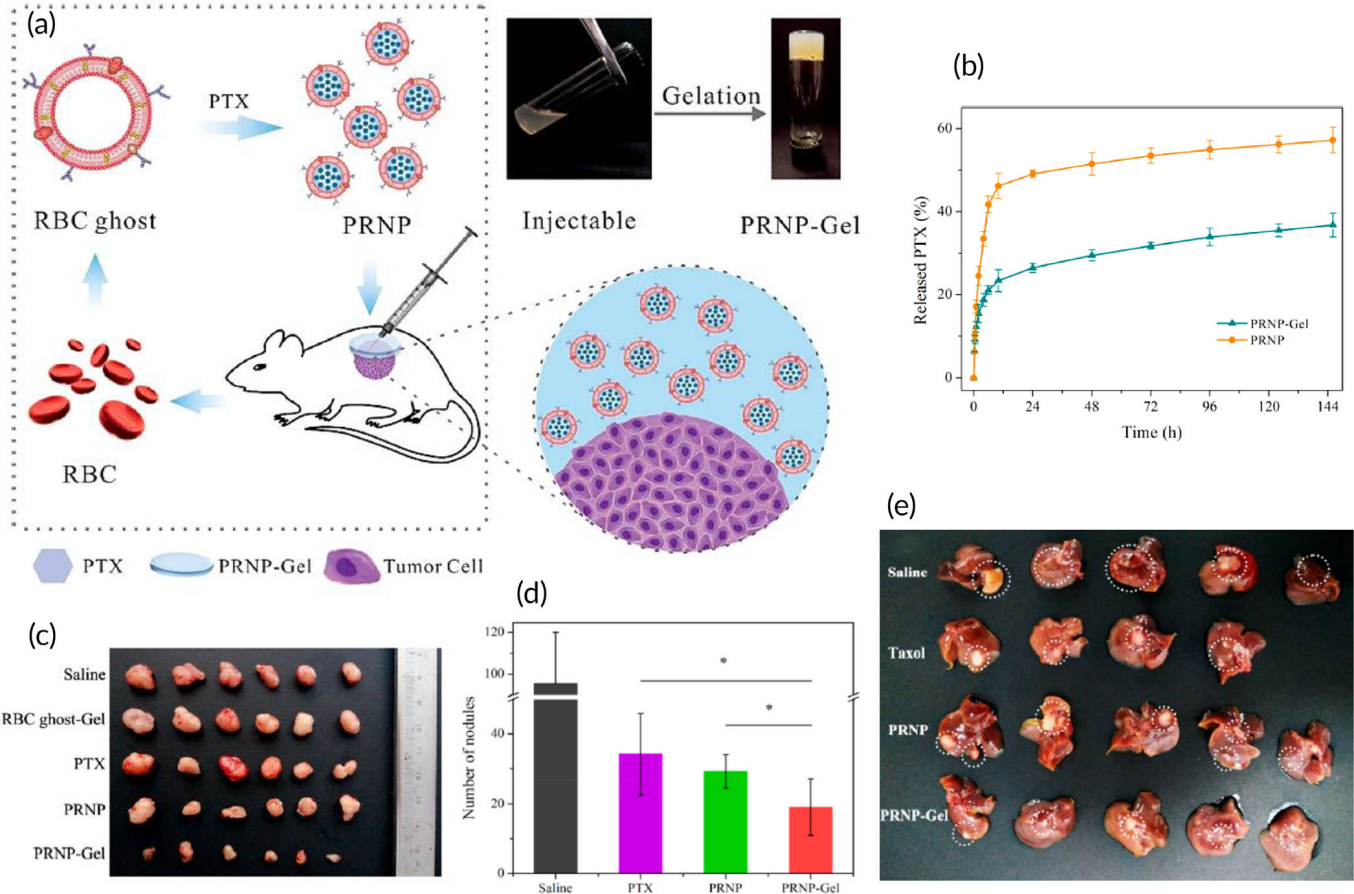
Red blood cell‐mimicking nanosystems for treatment of gastric cancer with peritoneal dissemination and metastasis. (a) Schematic illustration of the fabrication of PRNP‐Gel by coating paclitaxel‐loaded bovine serum albumin NPs with red blood cell membranes. (b) In vitro comparison of paclitaxel release from PRNP‐Gel (blue) and PRNP (orange). (c) Photographs of tumor tissues after treatment with different agents. (d) Number of peritoneal nodules in tumor‐bearing mice models of gastric cancer with peritoneal dissemination. (e) Photographs of metastatic tumor tissues after different treatments (white circles are meant to highlight liver metastases). Reproduced with permission.^112^ Copyright 2019, American Chemical Society. PRNP, paclitaxel‐loaded polyethylene glycol (PEG)‐modified bovine serum albumin nanoparticle coated with a red blood cell membrane; PRNP‐Gel, injectable hydrogel; PTX, paclitaxel; RBC, red blood cell.

Peritoneal dissemination and metastasis are common complications in advanced stages of gastric cancer. In such cases, growing evidence has shown the superior therapeutic efficacy of local intraperitoneal chemotherapy when compared to traditional systemic chemotherapy, by enabling a prolonged and superior drug accumulation at the peritoneal area.^112^ In order to increase the retention of paclitaxel at the peritoneal cavity and the antitumor efficacy of intraperitoneal chemotherapy, injectable hydrogels (PRNP‐Gel) composed of paclitaxel‐loaded polyethylene glycol (PEG)‐modified BSA NPs coated with a red blood cell membrane, designated PRNP, were developed (Figure 11a).^112^ The injectable and degradable PRNP‐Gel formed at 37°C provided a sustained and controlled long‐term release of paclitaxel in vitro when compared to PRNP (Figure 11b). This could improve drug accumulation at targeted tumor sites for enhanced antitumor efficacy. In summary, local delivery of paclitaxel using the PRNP‐Gel showed to effectively inhibit tumor growth (Figure 11c,d) and the peritoneal dissemination and metastasis in mouse models of gastric cancer, as demonstrated by the remarkable reduction of the number of liver metastatic lesions in vivo (Figure 11e).^112^

Regarding gastric cancer therapy, biomimetic hybrid cell membrane‐coated NPs have also been investigated for co‐delivery of chemotherapeutic drugs and photothermal agents to yield a synergistic chemo‐PTT antitumor approach.^113^ To do this, hybrid cell membranes composed of red blood cell membranes and gastric cancer cell membranes were used to coat graphene oxide quantum dots (GOQDs) co‐loading indocyanine green (ICG, a photosensitizer) and gamabufotalin (CS‐6, a chemotherapeutic drug).^113^ The dual membrane‐coated biomimetic nanosystem showed to preserve the biofunctions of both cell membranes: the enhanced biocompatibility and prolonged systemic circulation of red blood cell membranes, and the homotypic tumor‐targeting properties of the gastric cancer cell membranes, for improved tumor‐targeting and tumor‐specific accumulation. The enhanced biocompatibility, biosafety and the chemo‐PTT combinational anti‐gastric effects of the biomimetic nanosystem (under NIR light irradiation) were observed both in vitro and in vivo.^113^

The VEGF expression level has been correlated with gastric cancer progression and metastasis.^114^ Increasing evidence demonstrated that the activation of the VEGFR2/STAT3 pathway can greatly accelerate tumor cell proliferation, angiogenesis, and metastasis. Hence, the inhibition of vascular endothelial growth factor receptor‐2 (VEGFR2) has been recognized as a key anticancer approach. Previous studies have shown that apatinib, a highly selective VEGFR2 inhibitor, can exert notorious antiangiogenic and antitumor effects by suppressing the VEGFR2/STAT3 pathway.^114^

Having this in mind, recently, a hybrid membrane obtained by fusing the membranes of HGC‐27 cells (a human gastric cancer cell line) with red blood cells was reported, which was successfully coated onto pH‐responsive liposomes co‐loaded with apatinib and cinobufagin, a compound with notorious antitumor activity by blocking several signaling pathways.^114^ The biomimetic nanostructure (LP‐R/C@AC) showed high biocompatibility, extended half‐life, immune evasion, and homotypic tumor‐targeting features, thus preserving the features of both cell membrane types. This resulted in enhanced drug accumulation at homologous HGC‐27 cells and metastatic tissues in vivo for efficient suppression of tumor growth, dissemination, and metastasis. The in vitro studies showed that LP‐R/C@AC could induce efficient antitumor effects by inhibiting the VEGFR2/STAT3 pathway. In summary, LP‐R/C@AC showed great promise as a combinatorial therapy against gastric cancer in both in vitro and in vivo.^114^

**FIGURE 12:**
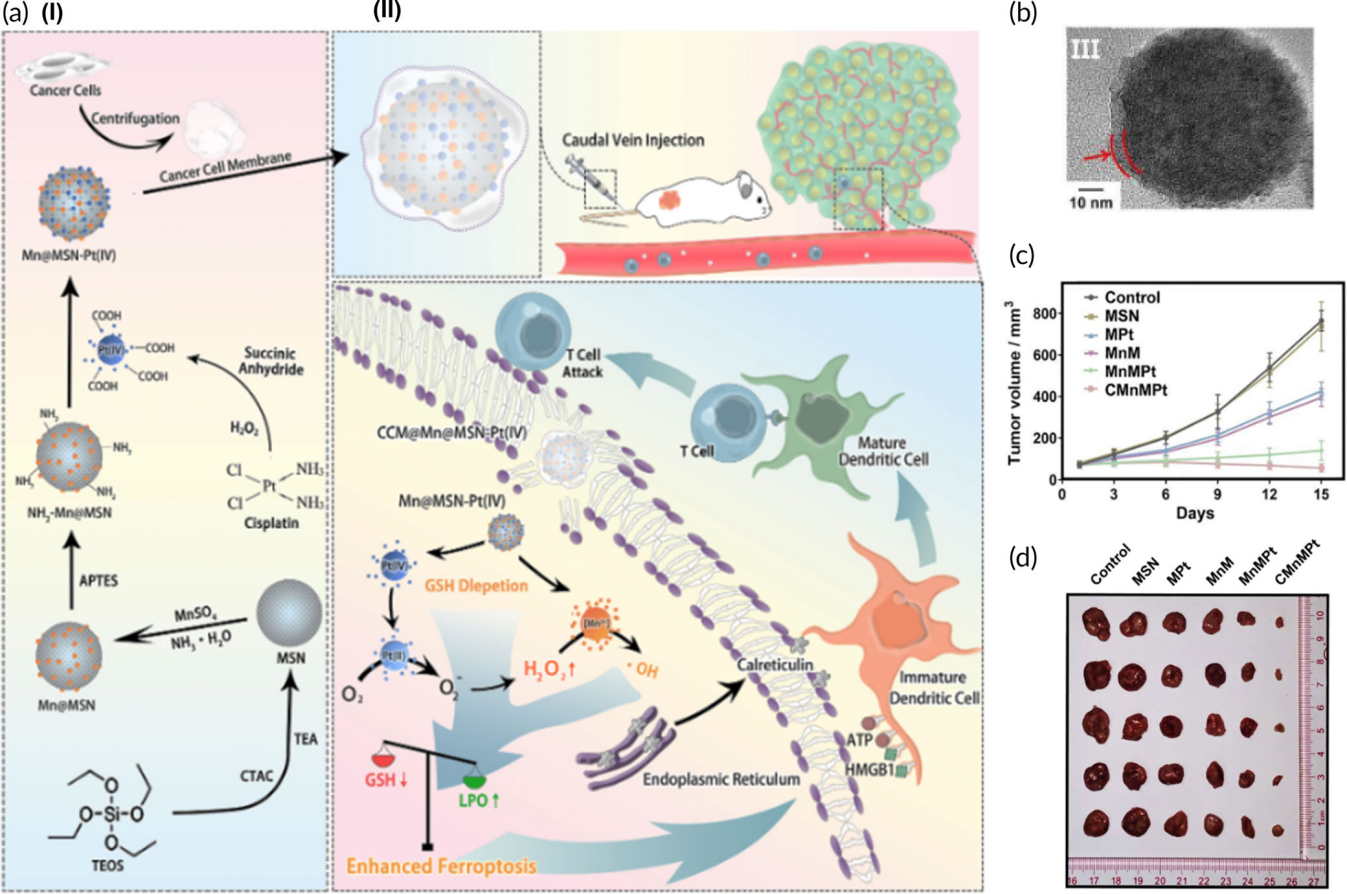
Gastric cancer cell membrane‐coated nanosystems for gastric cancer synergistic therapy by combining chemotherapy, CDT, and ferroptosis‐mediated ICD. (a) Schematic illustration of the (I) preparation of CCM@Mn@MSN‐Pt(IV) by coating Mn@MSN/Pt(IV) with gastric cancer cell membranes, and (II) in vivo homologous‐tumor targeting ability of CCM@Mn@MSN‐Pt(IV) after intravenous injection, and its Mn^2+^‐mediated CDT and ferroptosis‐mediated ICD combinatorial effects by self‐generating H_2_0_2_ and consuming intratumoral GSH. (b) TEM images of CCM@Mn@MSN‐Pt(IV), in which a core‐shell structure can be observed (red lines are meant to highlight membrane shell coating). (c) Tumor volume (mm^3^) curves after different treatments. (d) Photographs of tumor tissues after different treatments. Reproduced with permission.^115^ Copyright 2023, Elsevier. CDT, chemodynamic therapy; CMnMPt (also named CCM@Mn@MSN‐Pt(IV)), gastric cancer cell membrane‐coated MnMPt; GSH, glutathione; ICD, immunogenic cell death; MnM (also named Mn@MSN), Mn^2+^‐doped MSN; MnMPt (also named Mn@MSN/Pt(IV)), Pt(IV)‐loaded MnM; MPt, Pt(IV)‐loaded MSN; MSN, mesoporous silica nanoparticle; TEM, transmission electron microscopy.

Ferroptosis has been shown to improve immunogenicity of tumors, by inducing immunogenic cell death (ICD), a type of cell death that triggers potent antitumor responses by stimulating the maturation of dendritic cells and tumoral infiltration of cytotoxic CD8^+^ T‐cells. In a study aimed at developing a biomimetic nanosystem for gastric cancer therapy by combining chemotherapy, CDT, and ferroptosis‐mediated ICD, Mn2^+^‐doped MSNs (named Mn@MSN or MnM) loading cisplatin prodrugs (Pt(IV)) (termed as Mn@MSN/Pt(IV) or MnMPt), were coated by gastric cancer cell membranes, to achieve the goals of immune escaping and homotypic tumor‐targeting (Figure 12a‐I,b).^115^ Mn^2+^ have been applied as chemodynamic and ferroptosis‐inducing agents for CDT‐mediated ferroptosis. Mn^2+^ can decompose H_2_O_2_ into cytotoxic ROS (•OH) by the Fenton reaction, and the released ROS can oxidize the poly unsaturated fatty acid on cell membranes to produce lipid peroxidation—whose translation is inhibited by glutathione (GSH)—resulting in ferroptosis tumor cell death and cytotoxic T‐cells recruitment for ICD of tumors. This way, the efficacy of CDT‐mediated ferroptosis is directly related to the H_2_O_2_ and GSH intratumoral levels. Combination of Mn^2+^‐based NPs with Pt(IV) prodrugs can substantially improve the efficacy of CDT‐mediated ferroptosis by consuming intratumoral GSH and self‐supplying H_2_O_2_.^115^ Following uptake by homologous gastric cancer cells in vivo (Figure 12a‐II), the biomimetic nanosystem (named CCM@Mn@MSN‐Pt(IV) or CMnMPt) was degraded by consuming intratumoral GSH releasing Mn^2+^ and Pt(IV) prodrugs, which were converted to Pt(II)—the active compound capable of damaging nuclear DNA, and self‐generating H_2_O_2_ by triggering the activation of nicotinamide adenine dinucleotide phosphate oxidases (NOXs)—by again consuming GSH. This way, Pt(II) served as a self‐supplying H_2_O_2_ agent to augment •OH intratumoral levels via Mn^2+^‐mediated CDT, and the Pt(IV)‐to‐Pt(II) transformation could consume intratumoral GSH resulting in ferroptosis‐mediated ICD. Ultimately, this combinatorial biomimetic approach produced effective anti‐gastric cancer effects in vivo (Figure 12c,d).^115^

PDT has recently been combined with cell‐mimicking nanosystems to improve gastric cancer phototherapy. In this study, silica NPs were loaded with Ce6 (a photosensitizer), and then coated with SGC7901 gastric cancer cell membranes.^116^ The incorporation of the gastric cancer cell membrane endowed the final nanosystem with homotypic SGC7901 tumor‐targeting features both in vitro and in vivo, which resulted in increased intracellular uptake and tumor accumulation. The Ce6 exhibited efficient ROS‐induced cytotoxicity to SGC7901 gastric cancer cells upon NIR light irradiation. Hence, the biomimetic nanosystem showed notorious PDT effects and enhanced antitumor efficacy.^116^

**FIGURE 13:**
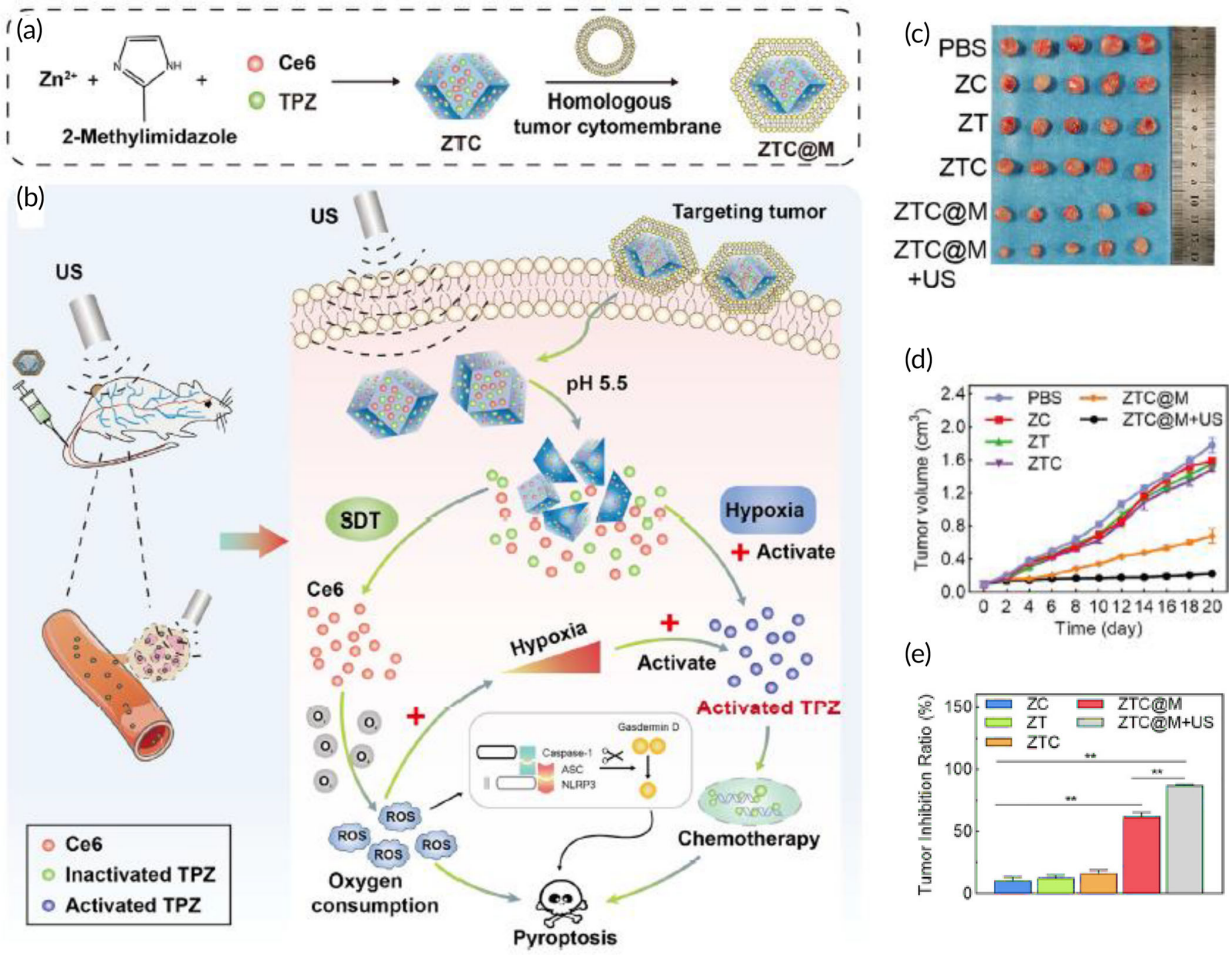
Gastric cancer cell membrane‐coated nanosystems for combined US‐triggered SDT and chemotherapy against gastric cancer. (a) Depicting of ZTC@M preparation by coating ZTC cores with AGS cell membranes (a human gastric adenocarcinoma cell line). (b) Illustration of the combinatorial effects of SDT and chemotherapy upon US irradiation for effective gastric cancer cell ablation. (c) Photographs of tumor tissues of AGS tumor‐bearing mice after different treatments. (d) Tumor volume (cm^3^) of AGS tumor‐bearing mice after different treatments. (e) Tumor inhibition rate (%) in AGS tumor‐bearing mice after different treatments. Reproduced with permission.^117^ Copyright 2022, Frontiers Media S.A. Ce6, chlorin e6; SDT, sonodynamic therapy; TPZ, tirapazamine; US, low‐intensity ultrasound; ZIF‐8, zeolitic imidazole framework‐8; ZTC, Ce6 and TPZ co‐loaded ZIF‐8 nanoparticle; ZTC@M, gastric cancer cell membrane‐coated ZTC.

Another study exploited the Ce6‐mediated sonocytotoxicity to enhance gastric cancer therapy by combining US‐triggered SDT and chemotherapy.^117^ This way, ZIF‐8 NPs were co‐loaded with Ce6 and prodrug tirapazamine (ZTC), and subsequently coated by gastric cancer cell membranes (ZTC@M) for gastric cancer‐targeted therapy by capitalizing on the homotypic tumor‐targeting features of the membrane cloaking (Figure 13a).^117^ The synergistic antitumor effects of ZTC@M were related to the ability of Ce6 to consume tumoral O_2_ and convert it into cytotoxic ROS upon US irradiation. Besides, the Ce6‐mediated SDT could further aggravate hypoxic tumor microenvironment (due to the O_2_ consumption), required for activating the hypoxia‐activating tirapazamine to kill tumor cells (Figure 13b). The ZTC@M showed superior homing features to homotypic AGS gastric cancer cells and enhanced antitumor effects in vivo, showing to be more effective than non‐coated counterparts in suppressing tumor growth and inducing tumor cell apoptosis (Figure 13c–e). This study paved the way for the design of biomimetic NPs for improved gastric cancer therapy by combining US‐activated, Ce6‐mediated SDT and tirapazamine‐induced chemotherapy.^117^

### 6.3 Liver cancer

Liver cancer is a life‐threatening subtype of gastrointestinal cancer and ranks as the third deadliest cancer worldwide.^163^ The most prevalent type of liver cancer is hepatocellular carcinoma (HCC), a significant global cause of cancer‐related deaths.^164^ HCC is associated with a high morbidity and low survival rates.^165^, ^166^ The standard treatments for HCC are surgical resection of the tumor and liver transplantation (considered to be the only potential curative solutions). However, unfortunately, most patients are already in advanced stages at the time of diagnosis, making surgical resection and liver transplantation unavailable options. Systemic chemotherapy, radiotherapy, and transarterial chemoembolization (TACE) are another available approaches.^164^, ^165^ As the conventional therapies have not demonstrated efficient advantages in HCC treatment, considerable attention has been paid to various nanotechnology‐based delivery systems (including biomimetic NPs) for tumor‐targeted delivery of anticancer drugs. Several in vivo and in vitro evidences have demonstrated considerable tumor‐targeting efficacy, higher rate of drug cellular uptake, system toxicity attenuation, and immunocompatibility of anti‐HCC drugs. For instance, following the application of doxorubicin‐loaded PLGA NPs with a HepG2 cell membrane coating, it was proved that the tumor volume attenuation and anticancer properties were mainly attributed to improved cancer cells' wall targeting and direct delivery of the NPs to the site of interest via the biomembrane‐coated nanoformulation.^118^, ^119^

Similarly, in another study, pectin‐doxorubicin conjugates were self‐assembled into NPs and camouflaged with an erythrocyte membrane stealth coating through hypotonic dialysis and mechanical co‐extrusion methods.^120^ These biomimetic NPs were introduced as promising formulations for in vivo HCC therapy, due to their prolonged blood circulation and potency to remarkably attenuate the tumor volume 16 days after treatment.^120^

**FIGURE 14:**
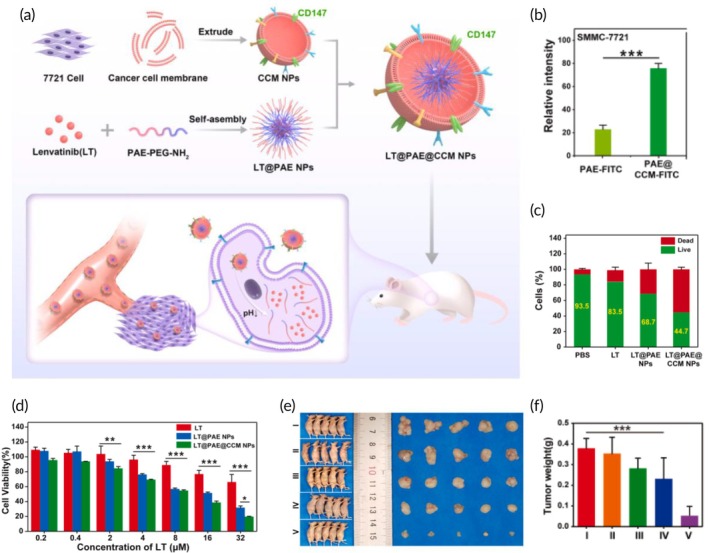
Liver cancer cell membrane‐coated pH‐sensitive nanosystems for liver cancer‐targeted chemotherapy. (a) Illustration of LT@PAE@CCM preparation by coating LT@PAE NP cores with SMMC‐7721 liver cancer cell membranes. (b) Tumor targeting ability after incubation with SMMC‐7721 cells. (c) Live and dead cells quantification (%) after treatment of SMMC‐7721 cells with PBS, LT, LT@PAE NPs, and LT@PAE@CCM NPs (green and red bars represent live cells and dead cells, respectively). (d) Cell viability of SMMC‐7721 cells after treatment with LT, LT@PAE NPs, and LT@PAE@CCM NPs (with LT increasing concentrations). (e) Photographs of tumor tissues of SMMC‐7721 tumor‐bearing mice after different treatments. (f) Tumor weight curves of SMMC‐7721 tumor‐bearing mice after different treatments. (I: PBS, II: PAE NPs, III: LT, IV: LT@PAE NPs, and V: LT@PAE@CCM NPs). Reproduced with permission.^122^ Copyright 2023, Elsevier. CCM, SMMC‐7721 liver cancer cell membrane; LT, Lenvatinib; LT@PAE NP, LT‐loaded PAE‐PEG‐NH_2_ nanoparticle; LT@PAE@CCM NP, CCM‐coated LT@PAE nanoparticle; PAE, poly(β‐amino ester); PAE‐PEG‐NH_2_, poly(β‐amino ester)‐polyethylene glycol‐amine.

Other chemotherapeutics have also been formulated in biomembrane‐coated top down nanosystems for HCC‐targeted therapy, such as paclitaxel.^121^ In one study, folate‐surface functionalized SMMC‐7721 liver cancer cell membranes were used to coat paclitaxel‐loaded nanocrystalline particles.^121^ The biomembrane‐coated nanoassemblies showed strong liver cancer‐targeting and accumulation features, which were attributed not only to the homotypic tumor‐targeting features of the membrane cloaking, but also to the affinity of the surface functionalized‐folic acid targeting ligand to specific receptors highly expressed on liver cancer cells. Superior immune escape, prolonged systemic circulation, and reduced tumor cell's growth in vivo were observed.^121^ Regarding biomimetic NPs' application for HCC‐targeted chemotherapy, encapsulating lenvatinib (a FDA‐approved tyrosine kinase inhibitor for liver cancer therapy) into SMMC‐7721 cancer cell membrane‐coated pH‐sensitive polymeric poly(β‐amino ester)‐polyethylene glycol‐amine (PAE‐PEG‐NH_2_) NPs, to originate biomimetic LT@PAE@CCM NPs (Figure 14a), revealed high levels of stability, biocompatibility and immune evasion, plus precise tumor targeting toward homologous SMMC‐7721 liver cancer cells (Figure 14b). This resulted in superior anticancer effects, including 81% cell death in the in vitro model (Figure 14c,d), and an 8‐fold reduction in tumor weight compared to the control group in the in vivo model (Figure 14e,f), demonstrating significant tumor eradication.^122^

In another study, a hybrid membrane system was designed by fusing the platelet membranes and the Huh‐7 liver cancer cell membranes, which were used to cloak liquid crystalline lipid NPs co‐loaded with sorafenib (a multi‐kinase inhibitor indicated for advanced‐stage HCC) and triptolide at the molecular ratio of nearly 10:1.^123^ Combination of both drugs produced synergistic effects for effective HCC therapy. The hybrid cell membrane cloaking conferred immune evasion, long blood circulation and homologous tumor‐targeting features, endowing the final nanosystem with superior liver cancer‐targeting. Significant hindrance in cancer cells' growth and tumor cell apoptosis induction was achieved.^123^

In an effort to develop biomimetic nanomedicines for HCC‐targeted drug delivery, an erythrocyte membrane‐cloaked gambogic acid‐loaded mPEG‐PLA nanosystem was developed. Enhanced stability, biosafety, longer retention time and enhanced cancerous cells' blockade with superior antitumor efficacy were observed both in vitro and in vivo following systemic injection in HepG2 tumor‐bearing mouse models, compared to non‐coated NPs.^124^

Bufalin, another plant‐derived anticancer compound with strong anti‐HCC potential, has been investigated in biomimetic NPs for in vivo HCC therapy.^125^ In a research, a platelet membrane‐coated nanoformulation was fabricated via conjugating PLGA NPs to chitosan oligosaccharide and subsequent self‐assembling with vitamin E polyethylene glycol succinate (TPGS) and bufalin through nanoprecipitation method. These positive surface charges porous bufalin‐loaded NPs were then functionalized by negatively charged platelet membranes, and their application contributed to considerable TGI both in vitro and in vivo by enhancing drug accumulation at targeted HCC tumor tissues.^125^

Dihydroartemisinin, a well‐studied anti‐malarial drug, has also been recently investigated for HCC therapy.^126^ Its anticancer potential and tumor cell apoptosis induction properties are produced via suppression of the nuclear factor kappa‐light‐chain‐enhancer of activated B cells (NF‐ĸB) activity and activation of the mitochondria‐dependent apoptosis pathway. Previous studies have documented that Fe^2+^ can augment its anticancer toxicity, since the reaction between Fe^2+^ and dihydroartemisinin generates high amounts of ROS, that induce mitochondrial and DNA damage, and tumor apoptosis. Having this in mind, HepG2 liver cancer cell membranes were used to cloak dihydroartemisinin‐encapsulated ferrous ion doped zeolitic imidazolate framework (ZIF‐8) NPs aiming the targeted delivery of dihydroartemisinin and Fe^2+^ to HCC cells.^126^ The biomembrane nanosystem showed improved immune evasion and homotypic‐tumor targeting features, resulting in remarkable anticancer effects in vitro and in vivo (about 90.8% suppression in cancerous cells' proliferation).^126^

Another application of biomimetic NPs in liver cancer treatment is utilizing them for targeted delivery of miRNAs. In a recent investigation, AuNPs surface‐functionalized by hyaluronic acid and camouflaged with red blood cell membranes were employed for carrying miR‐181b (RHAuNCs‐miRNA) to in vitro and in vivo HCC models.^127^ The results showed that RHAuNCs‐miRNA possess a good stability and could protect the embedded miRNA from enzymatic degradation. Moreover, following mild NIR laser irradiation, cellular uptake of the biomimetic NPs was improved and acceptable in vivo biodistribution and tumor proliferation blockade were achieved.^127^

Similar to other gastrointestinal cancers, biomimetic NPs have also been used to improve the efficacy of liver cancer‐targeted phototherapy.^128^ In this context, CAR‐T cells targeting glypican‐3 (GPC3^+^, a heparin sulfate proteoglycan, expressed in 75% of HCC samples) were first produced and following extraction of their membranes, were used to coat mesoporous silica NPs loaded with IR780 (a near infrared dye/photothermal agent).^128^ The biomimetic NPs showed considerable HCC specific‐targeting and PTT‐mediated anticancer effects both in vitro and in vivo due to its ability to generate heat under NIR laser irradiation.^128^

Another HCC‐targeted PTT strategy based on cell membrane‐coated NPs was developed. For this purpose, a SMMC‐7721 liver cancer cell membrane‐coated nanosystem co‐encapsulating sepantronium bromide (YM155, a small inhibitor of survivin) and graphene quantum dots (which act as photothermal agents for PTT) were developed.^129^ The liver cancer cell membrane coating endowed the nanosystem with improved tumor‐targeting properties to homotypic cancer cells, which were further augmented by surface functionalization with iRGD peptide (a tumor‐penetrating peptide). Following NIR light irradiation, the biomimetic NPs showed pronounced hyperthermia‐induced tumor destruction via graphene quantum dots photothermal activity, simultaneously releasing anticancer compound YM155 for efficient ablation of HCC cells.^129^

The double combination of chemotherapy and PTT has also been studied in HCC settings to exploit the synergistic effects of both anticancer modalities.^130^ In a study, a hybrid cell membrane‐cloaked nanosystem was designed for chemo‐PTT of liver cancer. To do this, the membranes of RAW 264.7 macrophages and H22 hepatic cancer cells were fused and used to camouflage the sorafenib‐encapsulated NIR‐responsive hollow copper sulfide NPs, which were surface modified with anti‐VEGFR antibodies.^130^ Following NIR light irradiation, significant chemo‐PTT effects were observed in cancer cells, leading to 94.3% hindrance in tumor cells proliferation. This inhibition in cancerous cells' growth and angiogenesis was drived by downregulation of phosphatidylinositol 3‐kinase/protein kinase B (PI3K/Akt) and Rat sarcoma virus/Raf/mitogen‐activated protein kinase/extracellular signal‐regulated kinase (Ras/Raf/MEK/ERK) signaling pathways.^130^

Likewise, blood cell‐derived membranes, including platelet membranes, have also exhibited promising characteristics for HCC‐targeted chemo‐PTT synergy.^131^ In a study, doxorubicin‐embedded polypyrrole NPs (which act as photothermal agents for PTT) were coated with platelet membranes. By combining the chemotherapeutical potential of doxorubicin and heat‐generating properties of polypyrrole NPs, considerable inhibitory effects on cancer cells' growth and metastasis were achieved under 808 nm laser irradiation.^131^

Tumor diagnostic imaging is crucial to develop accurate and precise anticancer therapies, and ensure the therapeutic efficacy of established therapy. In a study aimed at developing a biomimetic nanotherapy for simultaneous NIR fluorescence diagnostic imaging and HCC‐targeted PDT, PLGA NPs were loaded with hypocrellin B (a plant‐derived compound which act as a photosensitizer for PDT), and then cloaked by neutrophil membranes.^132^ The neutrophil‐mimicking nanosystem showed immune evasion and long retention time in vitro and in vivo, and due to the neutrophils ability to be recruited to inflammatory/tumors tissues, the coated nanoassemblies showed superior HCC‐targeting features and enhanced uptake by HepG2 liver cancer cells. In vivo studies revealed the strong anti‐HCC effects of the nanosystem via PDT following NIR light irradiation, activation of pro‐apoptotic proteins expression, and regulation of the junB proto‐oncogene (JUNB)/ROS signaling pathway in which, JUNB suppression by NPs contributed to higher rate of ROS generation, mitochondrial dysfunction, and tumor tissue expansion hindrance.^132^

Similarly, another hypocrellin B‐based biomimetic nanosystem was developed for HCC‐targeted PDT.^133^ In this study, HepG2 cancer cell membranes surface‐functionalized with transferrin (a tumor‐targeting ligand) were capable of improving the efficacy and safety of PDT interventions via enhancing photosensitisers' accumulation in homologous HCC tumor sites, when they were applied to cover hypocrellin B bionic PEG‐PLGA NPs. Long‐time stability, low cytotoxicity, and increased PDT‐triggered ROS generation were accomplished.^133^

**FIGURE 15:**
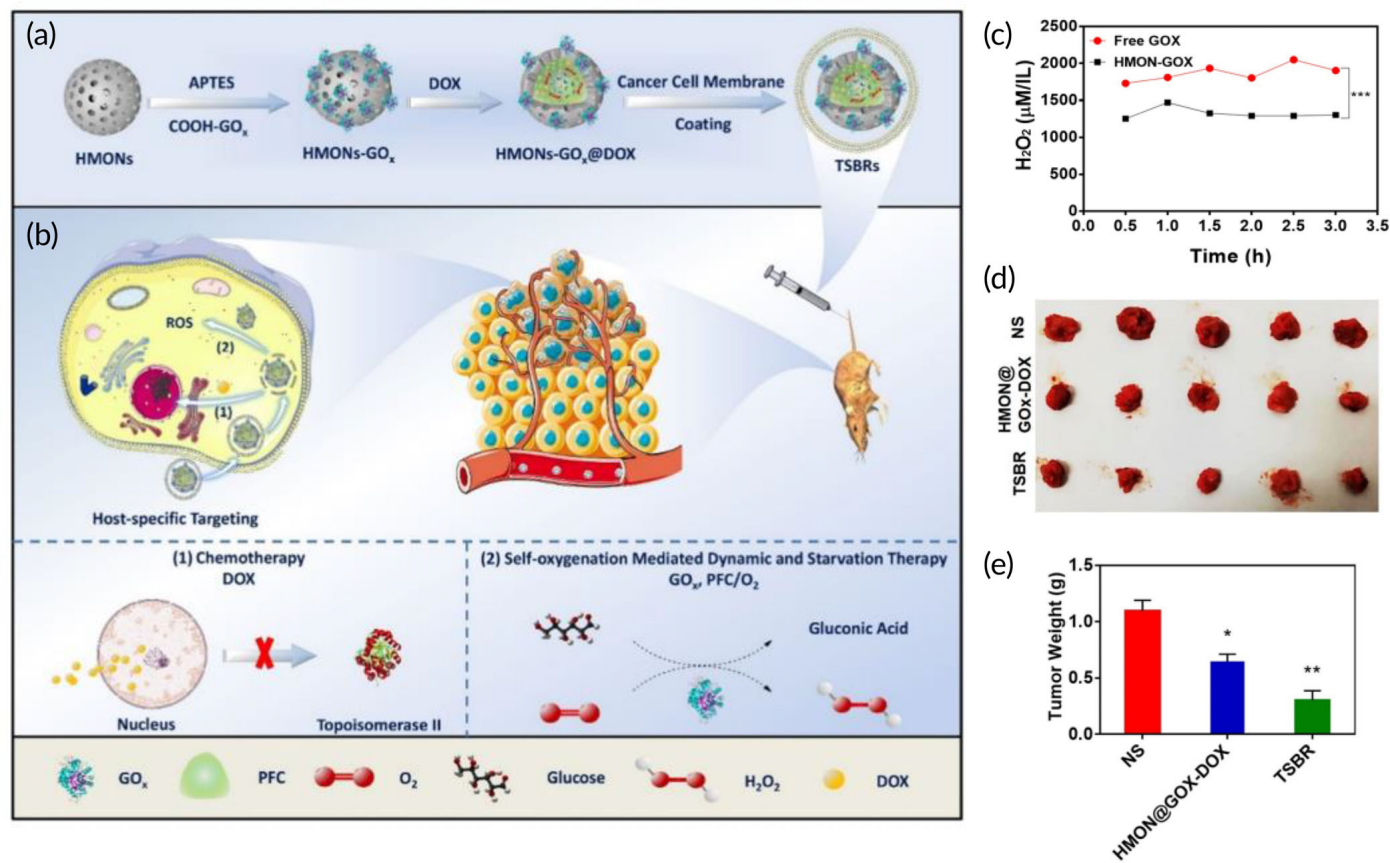
Liver cancer cell membrane‐coated nanosystems for dual chemo‐cancer starvation therapy of liver cancer. (a) Schematic illustration of the TSBRs synthesis by HepG2 liver cancer cell membrane coating. (b) Depicting of the anticancer effects of TSBRs following in vivo injection in tumor‐bearing mice: (1) The doxorubicin‐mediated chemotherapeutic effects via topoisomerase II inhibition and DNA synthesis suppression; (2) The self‐oxygenation effects of O_2_‐loaded perfluorocarbon, and the GOx‐mediated cancer starvation effects. (c) H_2_O_2_ concentration derived from the reaction between free GOx and HMONs‐GOx in glucose medium. (d) Photographs of tumor tissues of HepG2 tumor‐bearing mice after receiving NS, HMONs@GOx‐DOX NPs, and TSBR treatments. (e) Tumor weight curves of HepG2 tumor‐bearing mice after receiving NS, HMONs@GOx‐DOX NPs, and TSBR treatments. Reproduced with permission.^134^ Copyright 2023, Springer. DOX, doxorubicin; GOx, glucose oxidase; HMONs, hollow mesoporous organosilica nanoparticles; HMONs@GOx‐DOX, DOX‐loaded HMONs‐GOx; HMONs‐GOx, GOx surface‐modified HMONs; NS, normal saline; PFC, perfluorocarbon; TSBR, HepG2 liver cancer cell membrane‐coated HMONs@GOx‐DOX.

Cancer starvation therapy is another reported application of biomimetic cell‐mimicking NPs in the context of HCC‐targeted therapy.^134^ In a recent study, GOx surface‐modified hollow mesoporous organosilica NPs were loaded with chemotherapeutic drug doxorubicin and O_2_‐loaded perfluorocarbon (a O_2_ carrier for reducing tumor hypoxia and enhancing cancer starvation therapy efficacy), yielding O_2_‐PFC‐HMONs@GOx‐DOX NPs.^134^ These were further coated by HepG2 liver cancer cell membranes, for immune evasion and specific tumor targeting (Figure 15a). Following injection in tumor‐bearing mice, biomimetic NPs (TSBRs) could combine the doxorubicin‐mediated chemotherapeutic effects via DNA synthesis inhibition (Figure 15b‐1), with the GOx‐mediated cancer starvation via glucose and O_2_ conversion into gluconic acid and lethal H_2_O_2_, respectively (Figure 15c), and the intrinsic self‐oxygenation properties of O_2_‐loaded perfluorocarbon (Figure 15b‐2), to produce effective combinatorial anti‐HCC effects. Compared to other treatment groups, TSBRs showed the most remarkable tumor‐suppressing activity in vivo (Figure 15d,e), providing an effective biomimetic nanoplatform for HCC‐targeted dual chemo‐cancer starvation therapy.^134^

Surgical resection is considered a potential curative solution for early‐staged HCC patients. However, post‐surgical tumor relapse within 5 years is unfortunately very common among HCC postoperative patients (nearly 70% of patients suffer from postoperative tumor recurrence).^135^ Post‐surgical HCC relapse has been attributed to several factors, including the presence of residual tumor cells into the surgical margins after surgical interventions, that could escape and migrate through the bloodstream.^135^ To reduce the risk of post‐surgical recurrence of early‐staged HCC patients, a biomembrane‐coated nanosystem was designed. The wound‐targeted biomimetic nanodrug was prepared by coating MSNs loaded with sorafenib (a multi‐kinase inhibitor) with platelet membranes, previously modified with an immune checkpoint inhibitor (anti‐PDL1 antibody) via covalent attachment on membrane surface.^135^ Receptors on platelet membrane shell could specifically bind to collagen IV at wounded sites enabling the specific targeting to the post‐surgical wounded margins upon intraperitoneal injection. In vivo studies revealed the strong anti‐angiogenic and anti‐HCC effects of the biomimetic nanosystem, with two out of six post‐surgical HCC mice models being HCC‐free at day 70. Hence, the nanosystem showed promising results for suppressing postoperative HCC relapse.^135^

Biomimetic NPs have also been used for increasing the radiotherapy efficacy against liver cancer. In an interesting study, ultrasmall bismuth oxyiodide (BiOI) nanodots, camouflaged with M1 macrophage membrane (BiOI@M) have been applied to investigate their tumor targeting and radiosensitizing effects in HCC.^136^ The in vitro evaluations showed that BiOI@M are capable of absorbing more x‐ray energy, irradiated from low‐dose radiation, inside the tumors contributing to the existence of bismuth and iodine which highlights their potency for increasing the radiotherapy efficacy. Besides, the ultra‐small size of BiOI@M led to increased accumulation of the nanocarriers in the site of interest and rapid renal removal of BiOI@M which could avoid cytotoxicity, in vivo. In addition to these therapeutic potencies, BiOI@M have shown promising potential to be used as contrast agents via representing significant CT imaging ability to monitor the cancerous cells' proliferation and in vivo theranostic molecules' bio distribution visualization.^136^

**FIGURE 16:**
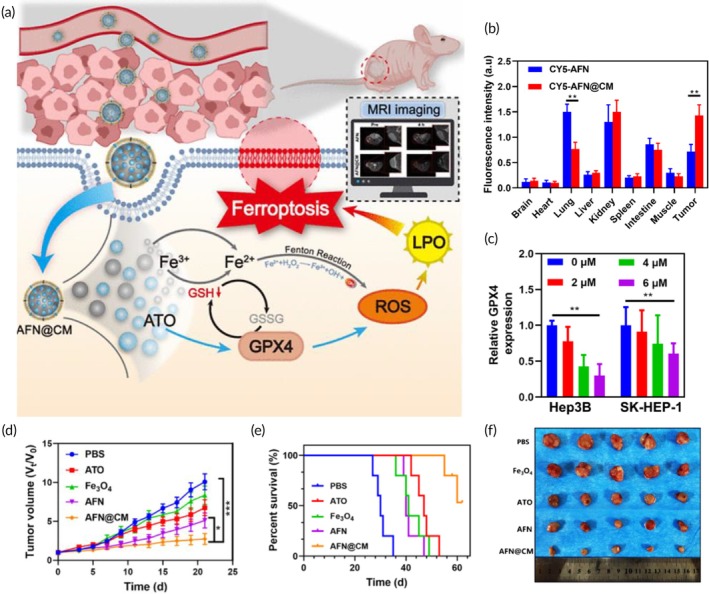
HCC cell membrane‐coated biomimetic nanosystems for HCC‐targeted cell death via ferroptosis. (a) Schematic depicting of the AFN@CM‐induced, HCC‐targeted ferroptosis via GPX4 inhibition and lipid peroxide intracellular accumulation, owing to the synergistic effects of arsenic trioxide and Fe_3_O_4_ NPs (ferroptosis inducers). (b) Fluorescence intensity of main organs after treatment of Hep3B tumor‐bearing mice with CY5‐AFN and CY5‐AFN@CMNPs. (c) Quantitative analysis of GPX4 expression in n Hep3B and SK‐HEP‐1 cells following AFN@CM treatment. (d) Tumor volume curves of Hep3B tumor‐bearing mice after different treatments. (e) Percent survival (%) of Hep3B tumor‐bearing mice after different treatments. (f) Photographs of tumor tissues of Hep3B tumor‐bearing mice after different treatments. Reproduced with permission.^137^ Copyright 2023, American Chemical Society. AFN, arsenic trioxide‐loaded Fe_3_O_4_ nanoparticle; AFN@CM, HCC cell membrane‐coated AFN; ATO, arsenic trioxide; Fe_3_O_4_ NP, iron oxide nanoparticle; GPX4, glutathione peroxidase 4; HCC, hepatocellular carcinoma; LPO, lipid peroxide; MRI, magnetic resonance imaging; ROS, reactive oxygen species.

Similar to other malignancies, ferroptosis induction in HCC tissues is a crucial potency of biomimetic NPs, which has been recently investigated.^137^ In a recent study, arsenic trioxide‐embedded Fe_3_O_4_ magnetic NPs (both potent ferroptosis inducers), abbreviated as AFN, were cloaked by HCC cell membranes, for immune escape and homologous targeting (Figure 16a). Resulting biomimetic NPs (AFN@CM) showed significant accumulation at the tumor tissue compared to non‐coated NPs (Figure 16b), also demonstrated by in vivo MRI, and revealed promising efficacy for increasing ROS and lipid peroxide intracellular accumulation for ferroptosis induction in HCC cells.^137^ While arsenic trioxide increased ROS and lipid peroxide accumulation via GSH and GPX4 consumption (Figure 16c), the Fe_3_O_4_ NPs could accelerate ROS and lipid peroxide accumulation via Fenton reaction, resulting in anticancer synergistic effects (Figure 16a). As a result, effective anti‐HCC and tumor suppression effects were achieved by AFN@CM‐induced ferroptosis (Figure 16d–f).^137^

### 6.4 Esophageal cancer

Esophageal cancer is currently one of the most prevalent and deadliest cancers worldwide, ranking as the sixth major cause of cancer‐associated mortality.^167^ There are two different types of esophageal cancer: esophageal squamous cell carcinoma (ESCC)—the most prevalent type of esophageal cancer globally—and esophageal adenocarcinoma (EAC).^36^, ^167^

Similar to other gastrointestinal cancers, nanotechnology‐based strategies—including the application of biomimetic NPs—have been gaining increasing attention in esophageal cancer treatment.^138^ In this context, researchers investigated the tumor‐killing potency of doxorubicin and curcumin co‐loaded PLGA NPs (constructed by solvent evaporation method), posteriorly camouflaged by TE10 esophageal cancer cell‐derived membranes and distearoyl phosphatidylethanolamine‐polyethylene glycol (named PMPNs), in doxorubicin‐resistant esophageal tumor models. Acceptable hindrance of tumor cells' proliferation has been observed in vitro, whereas in vivo assessments demonstrated improved tumor targeting and promising therapeutic potential.^138^

Chemo‐gene therapy has also been investigated in esophageal cancer settings.^139^ A study conducted recently has revealed the antitumor potential of pro‐inflammatory leukocyte membrane‐cloaked lipid nanovector (EYLN), which were used to co‐deliver doxorubicin and small interfering RNA (siRNA) targeting the anti‐lipid anabolic metabolism gene (siLPCAT1), to yield mEYLNs‐DOX/siLPCAT1.^139^ In vitro studies revealed considerable blockade of cancer cells' growth, migration, and metastasis, as well as enhanced NPs uptake by the target cancer cells. This was attributed to the presence of lymphocyte function‐associated antigen 1 (LFA‐1) on the leukocyte membrane. Regarding in vivo investigations, intravenous injection of the biomimetic nanocarriers in mice models led to improved tumor‐specific targeting and prolonged blood circulation time, resulting in significant tumor suppression, with the tumor volume in the treated group being approximately 20‐fold smaller than in the untreated group. Additionally, there was no evidence of toxicity, side effects in other organs, or significant changes in body weight.^139^

**FIGURE 17:**
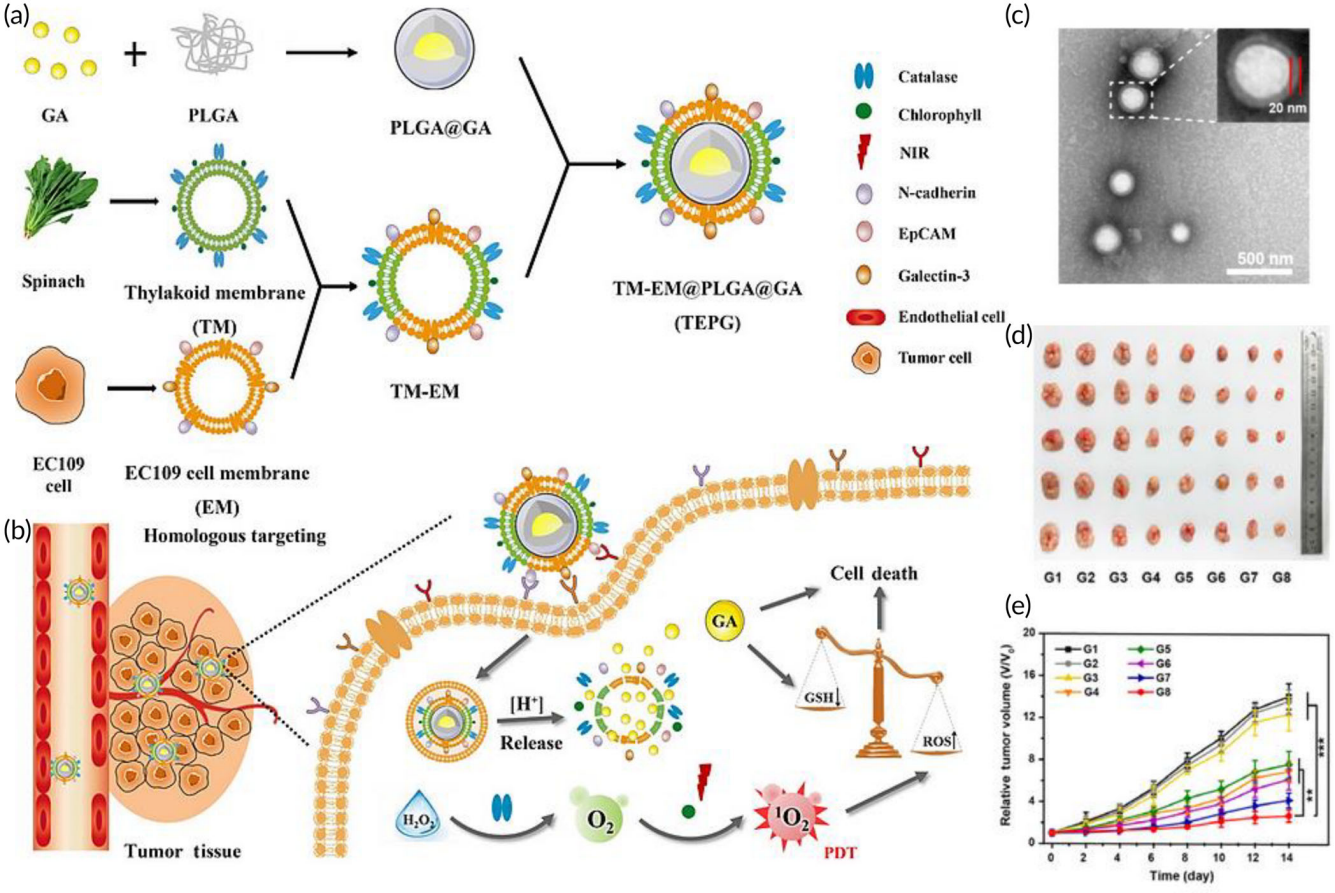
Hybrid thylakoid‐ESCC membrane‐coated nanosystems for ESCC‐targeted PDT. (a) Preparation of TM‐EM@PLGA@GA (or TEPG) by coating PLGA@GA cores with hybrid ESCC‐thylakoid membranes. (b) In vivo injection of TEPG NPs in ESCC‐bearing mice models, and their applicability for ESCC‐targeted PDT via effective delivery of photosensitizers to homotypic tumors, relieving tumor hypoxia and reducing intratumoral GSH. (c) TEM images of TEPG in which a core‐shell structure can be observed (red lines are meant to highlight membrane shell coating). (d) Photographs of ESCC tumor tissues after different treatments. (e) Tumor volume curves of ESCC tumor‐bearing mice after different treatments. (G1: Saline, G2: Saline + NIR, G3: TEP, G4: GA, G5: TEP + NIR, G6: TEPG, G7: TPG + NIR, and G8: TEPG + NIR). Reproduced with permission.^140^ Copyright 2023, Chinese Chemical Society. EM, ESCC membrane; ESCC, esophageal squamous cell carcinoma; GA, gambogic acid; GSH, glutathione; NIR, near‐infrared; PDT, photodynamic therapy; PLGA, poly lactic acid‐*co*‐glycolic acid; PLGA@GA, GA‐loaded PLGA nanosystem; TEP, TM‐EM‐coated PLGA; TM, thylakoid membrane; TM‐EM, hybrid thylakoid‐ESCC membrane; TM‐EM@PLGA@GA (also named TEPG), TM‐EM‐coated PLGA@GA; TPG, TM‐coated PLGA@GA.

Biomembrane‐coated NPs have also been studied in combination with cancer phototherapy to improve the efficacy of ESCC‐targeted PDT.^140^ The efficacy of this NIR light‐triggered approach is hindered by the reduced tumor‐targeted delivery of photosensitizers, the limited O_2_ intratumoral levels, as well as the neutralization of PDT‐generated ROS by excessive intratumoral GSH. To overcome these limitations, a biomimetic nanosystem, named TM‐EM@PLGA@GA (abbreviated as TEPG), was recently developed by coating PLGA cores carrying gambogic acid (a GSH‐consuming drug) (named PLGA@GA) with thylakoid‐EC109 esophageal cancer cell hybrid membranes (TM‐EM) (Figure 17a,c), aiming the effective delivery of photosensitizers to tumors, while simultaneously relieving tumor hypoxia and reducing intratumoral GSH levels.^140^ Thylakoid membranes (derived from green plants) are effective hypoxia reversers and photosensitizers for PDT owing to their O_2_ self‐generation ability. These properties are attributed to the presence of catalase and chlorophyll (a natural photosensitizer), which can catalytically decompose the intratumoral H_2_O_2_ into O_2_, and generating ROS under NIR light irradiation for PDT, respectively. After in vivo injection (Figure 17b), the biomimetic TEPG showed: (1) specific tumor‐homing and enhanced accumulation at ESCC tumors, due to the homotypic tumor‐targeting features of the ESCC membrane; (2) tumor hypoxia relief via the catalase‐mediated H_2_O_2_‐to‐O_2_ decomposition; (3) superior NIR‐induced PDT effects via chlorophyll photosensitivity, and (4) effective GSH depletion by gambogic acid. In vivo studies (Figure 17d,e) showed that the ESCC tumor growth suppression and killing abilities were considerably superior in the TEPG plus NIR light irradiation treatment group reaching a survival rate of 80% at day 34.^140^

### 6.5 Pancreatic cancer

**FIGURE 18:**
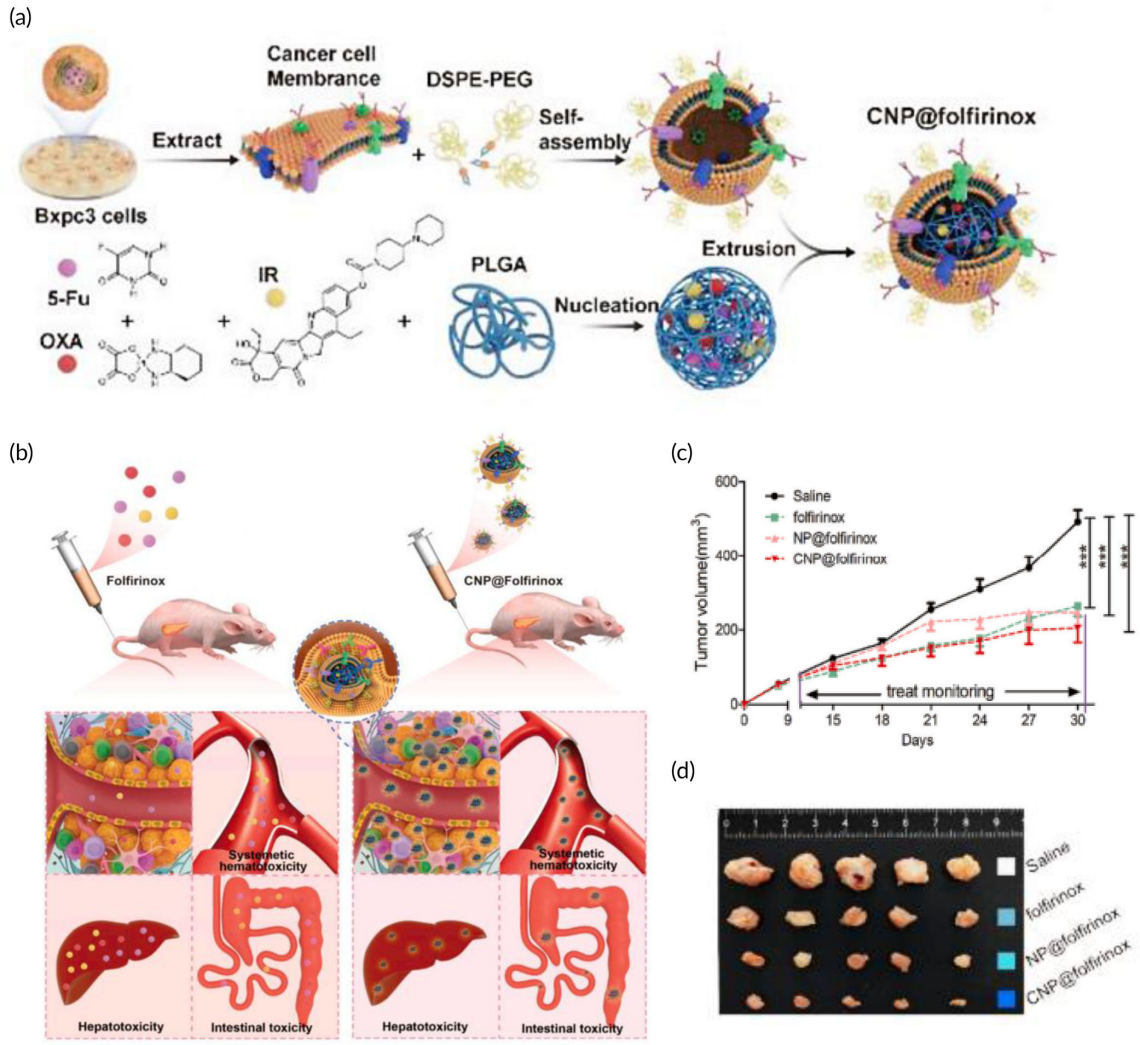
Pancreatic cancer cell membrane‐coated, FOLFIRINOX‐loaded nanosystems for improved and safer pancreatic cancer‐targeted chemotherapy. (a) Schematic illustration of CNP@folfirinox preparation by coating FOLFIRINOX‐loaded PLGA NPs with BxPC‐3 pancreatic cancer cell membranes. (b) Schematic depicting comparing the off‐target toxicity (hepatotoxicity and intestinal toxicity) of FOLFIRINOX‐based chemotherapy following in vivo administration of the FOLFIRINOX regimen and CNP@folfirinox. (c) Tumor volume curves of BxPC‐3 tumor‐bearing mice after different treatments. (d) Photographs of tumor tissues of BxPC‐3 tumor‐bearing mice after different treatments. Reproduced with permission.^141^ Copyright 2023, Elsevier. 5‐Fu, 5‐fluorouracil; CNP@folfirinox, BxPC‐3 pancreatic cancer cell membrane‐coated NP@folfirinox; IR, irinotecan; NP@folfirinox, FOLFIRINOX (5‐fluorouracil, irinotecan, and oxaliplatin)‐loaded PLGA nanoparticle; OXA, oxaliplatin; PLGA, poly (lactic‐*co*‐glycolic acid).

Pancreatic cancer is the seventh leading cause of cancer‐related mortality globally. Pancreatic ductal adenocarcinoma (PDAC), the most prevalent pancreatic cancer, is among the deadliest neoplastic diseases.^168^, ^169^ PDAC is featured by an immunosuppressive tumor microenvironment surrounded by a highly fibrotic stroma that severely compromises drug penetration and the success of established therapy. Current approaches for pancreatic cancer treatment include chemotherapy, radiotherapy, and surgical removal of the tumor.^169^, ^170^ Administration of chemotherapeutics is the preferred therapeutic approach in advanced and metastatic stages, and when surgery cannot completely remove the cancer.^168^ Systemic chemotherapy clinically employed in advanced PDAC includes single‐drug therapies (e.g., gemcitabine, paclitaxel, cisplatin, oxiplatin) and combinatorial therapies, such as the FOLFIRINOX regimen (5‐fluorouracil, leucovorin, irinotecan, and oxaliplatin), and gemcitabine plus nab‐paclitaxel. However, chemo‐based PDAC therapy remains unsatisfactory in both therapeutic efficacy and safety, due to drug resistance and off‐target toxicity.^171^ Hence, biomimetic nanomedicine‐based treatments, aiming to deliver the anti‐cancer agents more efficiently to the tumor site, have been used in pancreatic cancer. To increase the therapeutic efficacy and safety of the anti‐PDAC FOLFIRINOX strategy, the main components of the FOLFIRINOX regimen (namely 5‐fluorouracil, irinotecan, and oxaliplatin) were co‐loaded in PLGA NPs, and then camouflaged with BxPC‐3 pancreatic cancer cell membranes, producing biomimetic CNP@folfirinox (Figure 18a).^141^ In comparison to FOLFIRINOX i.v. administration, biomembrane‐coated, FOLFIRINOX‐loaded NPs showed pronounced immune escaping features, prolonged systemic circulation, and superior penetration and accumulation at homologous pancreatic cancer cells, thus overcoming off‐target toxicity of FOLFIRINOX‐based chemotherapy in healthy tissues (Figure 18b), and producing remarkable tumor growth suppression effects in vivo (Figure 18c,d).^141^

Anticancer compounds, such as chemotherapeutic drug gemcitabine^142^ and celastrol,^143^ have been also investigated in biomembrane‐coated nanosystems for PDAC therapy. Increasing evidence has demonstrated that the suppression of the EGFR signaling pathway leads to reduced pancreatic cancer growth, being therefore, recognized as a powerful anticancer approach.^142^ Combinational application of extracted macrophage cell membrane‐coated, gemcitabine‐embedded PLGA NPs and erlotinib (an EGFR inhibitor) in pancreatic cancer models indicated superior targeting of the nanocarriers to the tumor site, plus tumor cells' growth and angiogenesis impediment due to targeting the Ras/Raf/MEK/ERK and PI3K/Akt/ mammalian target of rapamycin (mTOR) signaling pathways, which together emphasizes their efficient therapeutic potency.^142^

In another study, PEG‐PLGA NPs were loaded with celastrol (a natural pentacyclic triterpenoid with reported anti‐PDAC potential), and then camouflaged by neutrophil‐derived membranes.^143^ This biomembrane‐coated nanotherapy led to higher survival rate, minimized liver metastasis, and tumor‐specific drug delivery due to the ability of immune neutrophil cell membranes to overcome the blood‐pancreas barrier in vivo. Moreover, strong blockade of tumor cells' growth has been observed, both in vitro and in vivo.^143^

**FIGURE 19:**
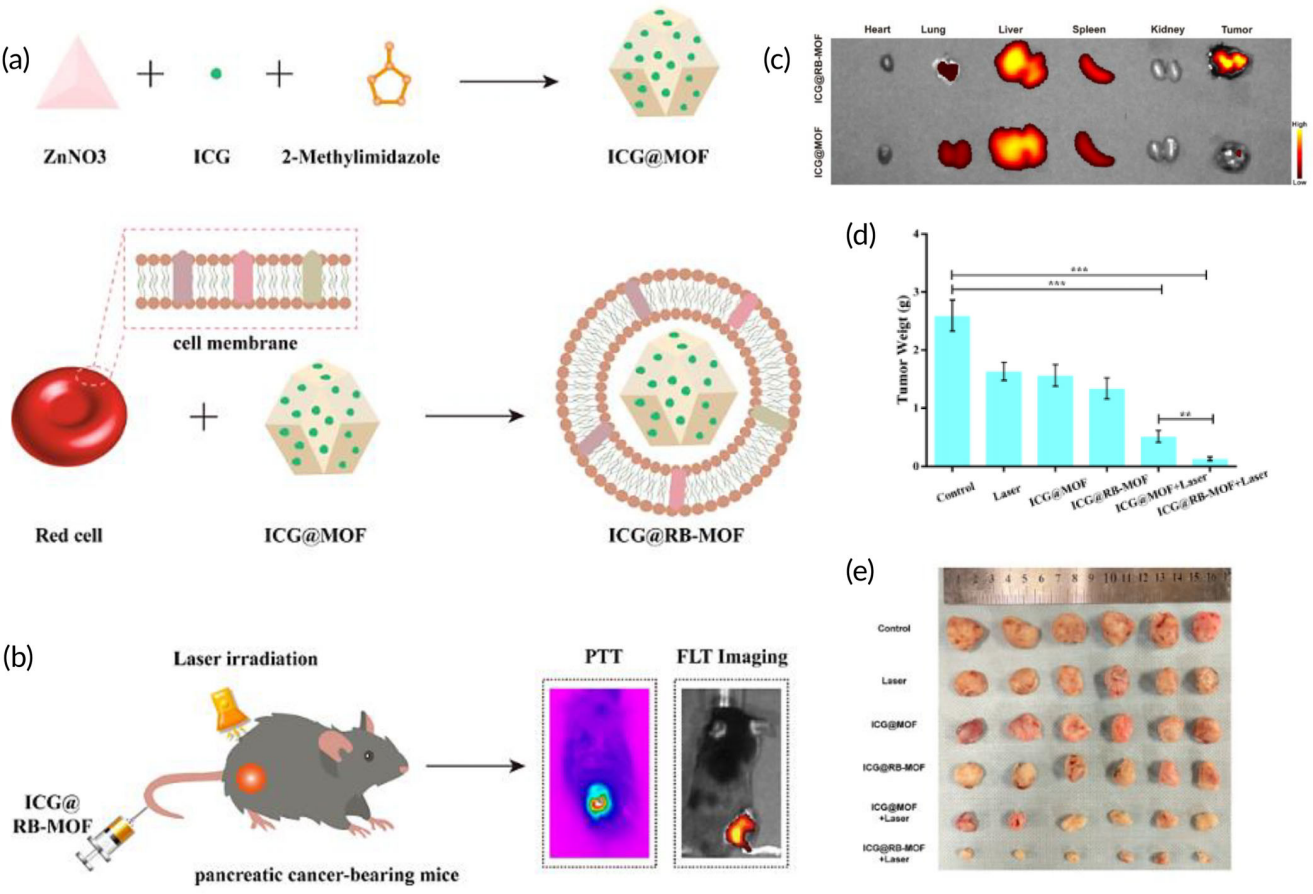
Red blood cell membrane‐coated nanosystems for pancreatic cancer‐targeted PTT and fluorescence imaging. (a) Preparation of ICG@RB‐MOF NPs by coating ICG@MOF NPs with red blood cell membranes. (b) In vivo injection of ICG@RB‐MOF NPs in pancreatic cancer‐bearing mice models, and their applicability for PTT and in vivo fluorescence imaging upon NIR laser exposure. (c) In vivo distribution of ICG@RB‐MOF NPs and ICG@MOF NPs at major organs and tumors 24 h after injection. (d) Tumor weight (g) after receiving different treatments. (e) Photographs of tumor tissues after different treatments. Reproduced with permission.^144^ Copyright 2023, Elsevier. FLT, fluorescence imaging; ICG, indocyanine green; ICG@MOF, ICG‐loaded MOF nanoparticle; ICG@RB‐MOF, red blood cell membrane‐coated, ICG@MOF; MOF, metal–organic framework (zeolitic imidazolate framework‐8) nanoparticle; PTT, photothermal therapy.

Biomembrane‐coated nanosystems have also been investigated for pancreatic cancer‐targeted PTT to enable targeted delivery and tumor‐specific accumulation of hyperthermia‐inducing photothermal agents.^144^ One study exploited the immune evasion and prolonged systemic half‐life of red blood cell membranes to enhance the tumor‐targeting ability of zeolitic imidazolate framework‐8 (MOF) NPs carrying ICG (a photosensitizer), named ICG@MOF NPs (Figure 19a).^144^ After in vivo injection, ICG was released in a pH‐responsive manner in the acidic conditions of the tumor microenvironment, and biomembrane‐coated ICG@MOF (ICG@RB‐MOF) NPs were employed for simultaneous NIR‐triggered PTT and fluorescence imaging (Figure 19b). The in vivo tracking of ICG@RB‐MOF NPs revealed a superior fluorescence intensity at tumors (by 3.84 times) compared to ICG@MOF NPs, indicating their enhanced tumor‐targeting and accumulation features (Figure 19c). Due to the heat‐generating property of ICG, a superior tumor suppression efficacy was reported in the ICG@RB‐MOF NPs plus NIR laser irradiation treatment group in vivo (Figure 19d,e).^144^

The combination of NIR two‐zone (NIR‐II) fluorescence imaging, chemotherapy, PTT, and PDT has also been investigated for PDAC theranostics.^145^ In this study, liposomes co‐loading chemotherapeutic drug doxorubicin and ICG (a photothermal agent for PTT and photosensitizer for PDT), named LIPO‐I/D, were camouflaged by SW1990 pancreatic cancer cell membranes, to yield a biomimetic core‐shell nanosystem (BLIPO‐I/D) with enhanced targeting features to homologous SW1990 pancreatic cancer cells.^145^ The ICG‐mediated NIR‐II fluorescence imaging enabled the in vivo tracking of BLIPO‐I/D, which revealed a superior fluorescence imaging intensity at tumors compared to LIPO‐I/D. Exposure to NIR light irradiation (808 nm) could disrupt BLIPO‐I/D structure, rapidly releasing doxorubicin for chemotherapy, and improving the ICG‐mediated photothermal and photodynamic effects. By combining the antitumor chemotherapeutic effects of doxorubicin, and the heat‐generating and cytotoxic ^1^O_2_‐producing properties of ICG under NIR irradiation, enhanced antitumor effects and superior suppression of tumor growth were observed in vivo.^145^

The fibrotic stroma in PDAC has been regarded as a substantial hurdle toward successful therapy, as it hinders drug delivery and intratumoral penetration. The dense PDAC desmoplastic stroma reaction leads to dense extracellular matrix, reduced vascularization and hypoxia, overall restricting drug accessibility to tumors.^171^ Hence, the combination of stromal remodeling strategies and conventional anti‐PDAC therapies (e.g., chemotherapy, immunotherapy, and gene therapy) have proven successful for effective PDAC management.

Having this in mind, erythrocyte‐extracted membranes have been used for covering various kinds of nanodelivery systems in order to enhance their tumor‐targeting and pancreatic cancer‐fighting profiles in both chemotherapeutic agents' delivery, PTT strategy and fibrotic stromal remodeling.^146^ In an interesting investigation, the researchers demonstrated that the application of erythrocyte membrane‐cloaked gold nanorods combined with cyclopamine (a strong tumor microenvironment modulator) results in significant disruption of pancreatic cancer dense extracellular matrix and better tumor blood perfusion. Furthermore, notable improvement in PTT efficacy, NPs' accumulation at tumor site (by 1.8‐fold), plus prolonged circulation time and tumor cell reduced size have been observed in vitro and in vivo, respectively.^146^

Likewise, in another study, employing erythrocyte membrane‐coated PLGA NPs, loading cyclopamine (a hedgehog signaling pathway inhibitor), in pancreatic cancer cells and mouse models showed significant tumor microenvironment modulation, prolonged circulation time and effective delivery of the NPs to the site of interest plus enhanced functional vessels and tumor perfusion. More interestingly, the research results demonstrated considerable in vivo tumor growth hindrance following combination of these nanodelivery systems with paclitaxel‐encapsulated erythrocyte membrane‐camouflaged PLGA NPs.^147^

**FIGURE 20:**
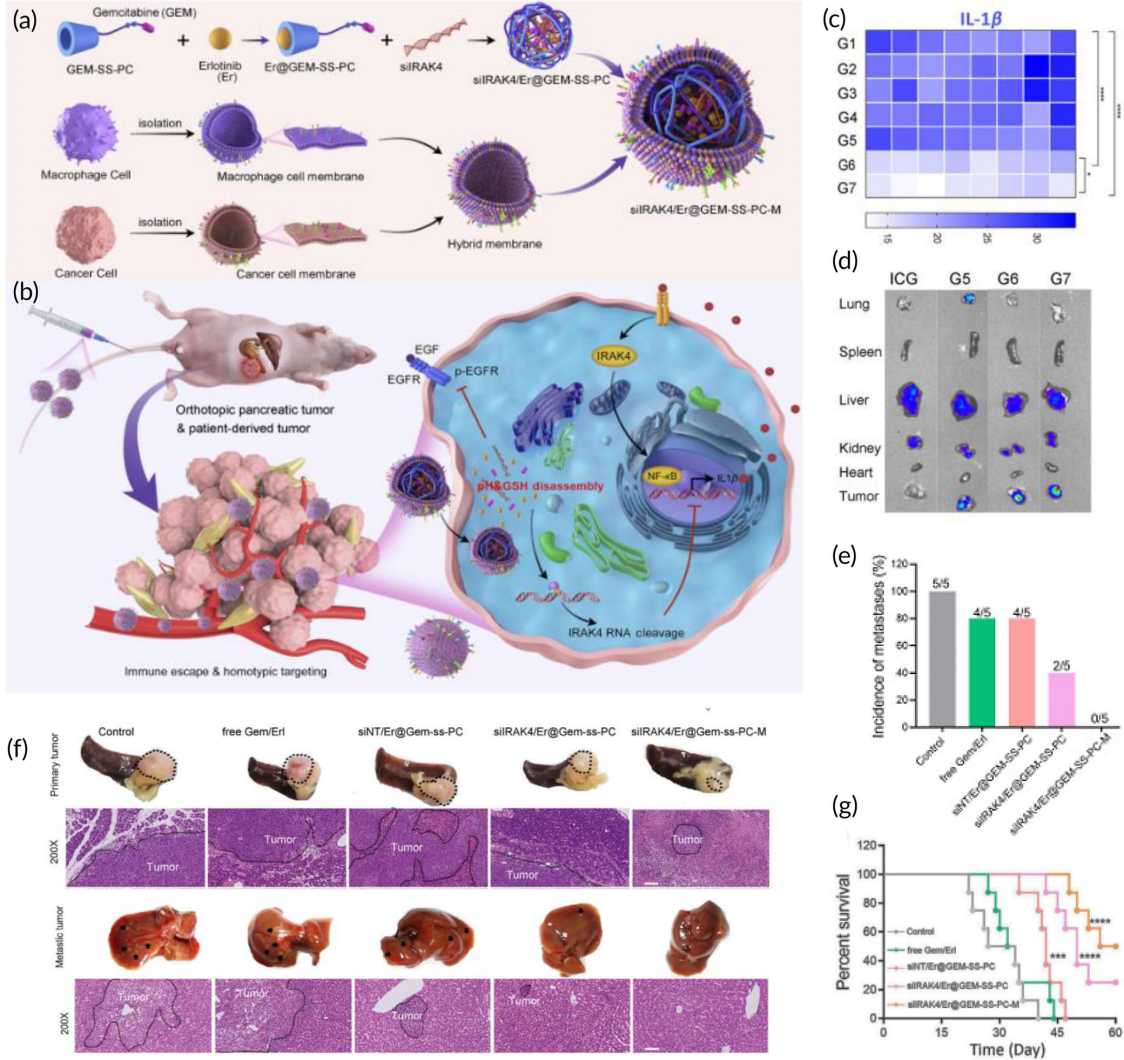
Cancer cell‐macrophage hybrid membrane‐coated nanosystems for pancreatic cancer‐targeted combinatorial therapy via chemotherapy, molecular‐targeted therapy and stroma remodeling (anti‐fibrotic gene therapy). (a) Schematic illustration of siIRAK4/Er@GEM‐SS‐PC‐M preparation by coating siIRAK4/Er@GEM‐SS‐PC with SW1990 pancreatic cancer cell‐RAW264.7 macrophage hybrid membranes. (b) Schematic depicting of the immune escaping, homotypic tumor‐targeting and anticancer effects after in vivo injection of siIRAK4/Er@GEM‐SS‐PC‐M in SW1990 tumor‐bearing mice. (c) Analysis of IL‐1β secretion assessed by ELISA after different treatments. (d) In vivo fluorescence imaging of main organs and tumors of the tumor‐bearing mice receiving different treatments. (G1: PBS, G2: Gem, G3: Er, G4: Free Gem and Er, G5: SiNT/Er@GEM‐SS‐PC, G6: SiIRAK4/Er@GEM‐SS‐PC, and G7: SiIRAK4/Er@GEM‐SS‐PC‐M). (e) Incidence of liver metastases (%) of SW1990 tumor‐bearing mice after different treatments. (f) Photographs of excised SW1990 tumors after different treatments, and H&E staining of pancreatic tissues (up), and photographs of excised livers after different treatments, and H&E staining of liver metastasis (down). (g) Percent survival (%) of SW1990 tumor‐bearing mice after different treatments. Reproduced with permission.^148^ Copyright 2022, Elsevier. EGFR, epidermal growth factor receptor; Er, erlotinib; GEM, gemcitabine; GEM‐SS‐PC, GEM‐based polymer prodrug; siIRAK4, siRNA targeting IRAK4; siIRAK4/Er@GEM‐SS‐PC, siIRAK4 and Er co‐loaded GEM‐SS‐PC; siIRAK4/Er@GEM‐SS‐PC‐M, SW1990 pancreatic cancer cell‐RAW264.7 macrophage hybrid membrane‐coated siIRAK4/Er@GEM‐SS‐PC.

In a recent attempt to increase the synergistic effects of chemotherapy, molecular‐targeted therapy and anti‐fibrotic gene therapy for pancreatic cancer‐targeted therapy and fibrotic stroma remodeling, a cationic polymer gene vector has been fabricated by assembling β‐cyclodextrin (β‐CD) with low‐molecular‐weight polyethylenimine (PEI), which turned into a polymer prodrug following conjugation with chemotherapeutic drug gemcitabine through GSH‐responsive linker (GEM‐SS‐PC).^148^ After encapsulating erlotinib (an EGFR inhibitor) and siRNA targeting IRAK4 (siIRAK4), the prepared nanodrugs (siIRAK4/Er@GEM‐SS‐PC) were camouflaged by SW1990 pancreatic cancer cell‐RAW264.7 macrophage hybrid membranes, originating biomimetic NPs (siIRAK4/Er@GEM‐SS‐PC‐M), aiming to reduce immune clearance, prolong systemic circulation and enhance homologous tumor‐targeting (Figure 20a,b). Interleukin‐1β (IL‐1β)‐IRAK4 signaling pathway has been regarded as crucial for extensive and dense fibrotic stroma, restricted intratumoral drug delivery, chemoresistance, and therapeutic failure. IRAK4 blockade and/or silencing has the potential to suppress NF‐ĸB activity, reducing fibrosis and increasing tumor chemosensitivity. The results showed that these nanocarriers performed their cancer‐fighting functions mostly through the combinatorial anticancer effects of gemcitabine and erlotinib, which were augmented via pancreatic cancer fibrotic stroma modulation—attributed to NF‐ĸB‐mediated IL‐1β production hindrance via IRAK4 suppressive activity (Figure 20c).^148^ In tumor‐bearing mice models, intravenous infusion of siIRAK4/Er@GEM‐SS‐PC‐M resulted in increased accumulation at homologous SW1990 tumors (Figure 20d), superior anticancer efficacy with considerable eradication of both SW1990 tumor cells and liver metastatic lesions (Figure 20e,f), and enhanced survival rate (Figure 20g). Hence, this study provides an innovative biomimetic nanosystem for pancreatic cancer‐targeted combinatorial therapy via chemotherapy, molecular‐targeted therapy and fibrotic stroma remodeling (anti‐fibrotic gene therapy).^148^

The shape design of different nanocarriers is an important factor to be considered for enhancing tumor‐specific targeting and inhibition.^149^ In a study, chemotherapeutic drug doxorubicin‐encapsulated BxPC‐3 cancer cell membrane‐cloaked nanorods, showed higher endocytosis efficiency due to the caveolin‐mediated pathway, increased drug accumulation in the endoplasmic reticulum region and higher drug concentration in the nucleus, when compared with nanospheres, contributing to endoplasmic reticulum stress and apoptosis induction. In addition, excellent immune evasion and extracellular matrix penetration were observed following intravenous injection of the nanoformulation in BxPC‐3 and pancreatic stellate cell hybrid tumor‐bearing nude mouse models, resulting in significant tumor eradication.^149^

Proteolysis‐targeting chimeric (PROTAC) is a strategy for pathologic proteins degradation through modulating the activity of endogenous ubiquitin‐proteasome system. Kirsten RAS (KRAS) mutation occurs in 95% of pancreatic cancers, however, direct targeting of KRAS represents a major challenge in pancreatic cancer therapy. PDEẟ shuttling factor, on the hand, has captured attention due to its potential to block the KRAS's binding to endomembranes and subsequent diffusion throughout the cell.^150^ Recently, a novel smart biomimetic nanodelivery system was developed for effective delivery of PROTAC‐induced PDEẟ degrader (PIPD), (CM8988‐PIPD), to pancreatic cancer cells. Application of cancer cell membrane‐coated NPs resulted in controlled drug release, acceptable biocompatibility and immunocompatibility, remarkable serum stability, apoptosis induction, and considerable cancerous cells' growth blockade through RAS signaling pathway inhibition, both in vitro and in vivo.^150^

Nanosecond pulsed electric field (nsPEF) has been recognized as a promising, non‐invasive and selective anticancer strategy. nsPEF technology employs a high electric field with ultra‐short pulses (nanosecond duration) to destroy tumor cells. This bioelectrical and non‐thermal technology induces tumor cell ablation via several mechanisms, which are mainly attributed to the ability of nanosecond electric pulses to alter the permeability and electrical properties of the membranes of tumor cells and intracellular organelles. This induces pores' formation and tumor cell apoptosis. Despite the considerable success of nsPEF technology for cancer therapy, some limitations exist, such as the risk of remaining residual tumors when insufficient electric energy is employed.^151^ Thus, combining nsPEF with other anticancer therapies has been studied to produce synergistic effects and greatly enhance antitumor efficacy. Recently, neutrophil membrane‐coated, gemcitabine‐loaded liposomal NPs have been engineered and employed as a complementary strategy to nsPEF for anti‐PDAC therapy.^151^ The biomimetic NPs could efficiently accumulate in nsPEF‐pre‐treated PDAC cells, due to the tumor‐targeting features of the neutrophil membrane, which were amplified by the nsPEF‐triggered release of pro‐inflammatory signals (e.g., tumor necrosis factor‐α [TNF‐α]). Thus, by combining nsPEF and chemotherapeutics delivery via biomembrane‐coated NPs, this study provided an effective anti‐PDAC approach.^151^

## 7 OTHER BIOMEMBRANE‐COATED NANOTECHNOLOGICAL APPROACHES FOR GASTROINTESTINAL CANCERS

In addition to natural cell membranes, other biomembrane sources have been exploited in this top‐down coating strategy. These range from cell‐secreted extracellular vesicles (exosomes), to bacterial membranes and bacterial OMVs (secreted by Gram‐negative bacteria).

Exosomes are small extracellular vesicles naturally secreted by eukaryote cells with prominent roles in cell‐to‐cell communication.^172^ These nanosized vesicles have captured attention as biomimetic coatings for NPs, as they are naturally secreted in an optimized nanosize with intrinsic biocompatibility, stability, and cell‐targeting features.^15^, ^16^

In addition to mammalian cells and mammalian cell‐secreted exosomes, bacteria have also been investigated in this field. Although there are fewer research studies on bacterial membrane‐coated NPs than on mammalian cell membrane‐coated NPs, bacterial membranes have emerged as attractive coating biomaterials for nanomaterials.^63^, ^173^

Bacterial OMVs are membrane‐shaped structures naturally secreted by Gram‐negative bacteria (from the bacteria's outer membrane), which play a vital role in mediating bacterial communication. Bacterial OMVs have also been investigated for NP coatings.^64^

Table 8 summarizes the studies employing OMV‐, bacterial membrane‐, and exosomal membrane‐coated biomimetic NPs for the management of gastrointestinal cancers.

**TABLE 8:** Summary of the studies employing OMV‐, bacterial membrane‐, and exosomal membrane‐coated biomimetic nanocarriers for the treatment of gastrointestinal cancers.

Cancer type	Therapeutic strategy	Membrane source	Inner core	Drug(s)	Coating method	Size/zeta potential	In vivo tumor model	Outcomes	Refs.
Colorectal cancer	Chemotherapy	*Escherichia coli*‐secreted OMV	MSN	5‐Fluorouracil	Co‐extrusion	90.4 ± 9.1 nm −18.22 ± 0.17 mV	HT‐29 tumor‐bearing mouse model	Immune evasion Targeted delivery to the colorectum ↓ tumor growth ↓ off‐target toxicity	174
	Imaging and targeted chemotherapy	*Lactobacillus reuteri* biomembrane	ZGGO@SiO_2_ NP	5‐Fluorouracil	Co‐extrusion through 400 and 200 nm porous membranes (3 cycles)	164.0 nm −3.90 mV	Colorectal tumor‐bearing mouse model	Immune evasion ↓ gastric acid digestion Sustained and controlled drug release Targeted delivery to the colorectum ↓ tumor growth ↑ survival time of mice	175
	SDT and Ca^2+^‐induced tumoral mitochondria damage	CT26 colon cancer cell‐derived exosome membranes	CaCO_3_ NP	Curcumin (sonosensitizer)	Direct incubation of CT26 colon cancer cells with NPs	—	CT26 tumor‐bearing mouse model	Prolonged systemic circulation time ↑ Immune escape ↑ Tumor targeting ↑ Tumor site accumulation	61
Liver cancer	Targeted molecular therapy	huh‐7 liver cancer cell‐derived exosome membrane	PEG‐PLGA NP	Sorafenib	Sonication	231.79 ± 20.09 nm −27.29 ± 1.46 mV	H22 tumor‐bearing mouse model	↑ Tumor site accumulation ↑ Anticancer effects ↑ Tumor penetration ↓ Tumor volume	176

Abbreviations: CaCO_3_, mesoporous calcium carbonate; MSN, mesoporous silica nanoparticle; NP, nanoparticle; OMV, outer membrane vesicle; PEG‐PLGA, PEGylated‐poly (lactic‐*co*‐glycolic acid); SDT, sonodynamic therapy; ZGGO@SiO_2_, persistent luminescence mesoporous silica; ↑, enhancement; ↓, reduction.

### 7.1 Colorectal cancer

Although there have been many studies regarding NPs coated with cell membranes for colorectal cancer therapy, coating nanosystems with bacterial biofilms,^175^ and OMVs secreted by Gram‐negative bacteria have also been reported.^174^

For instance, in a study aimed at developing bacterial OMV‐coated NPs for colorectal cancer therapy, *Escherichia coli* (*E. coli*)‐secreted OMVs were selected as membrane coatings for MSNs loading 5‐fluorouracil (a thymidylate synthase inhibitor chemotherapeutic drug).^174^ The bacterial‐bioinspired nanosystem could preserve the high loading capacity of MSNs and the enhanced intestinal absorption of *E. coli*‐secreted OMVs, improving the intestinal absorption of the biomimetic nanosystem, while remaining undetected by the mononuclear phagocyte system. While the OMV‐coated biomimetic nanosystem was mainly absorbed in the colon, non‐coated NPs were distributed equally throughout the intestinal tract. In addition to the intrinsic targeting properties of OMVs, the tumor‐targeted delivery of 5‐fluorouracil and selective uptake by HT‐29 colon cancer cells were also attributed to the surface modification of MSNs with hyaluronic acid molecules. This improved active tumor‐targeting and selective cell uptake via CD44 receptor‐mediated endocytosis pathway. Hence, OMV‐coated NPs concentrated mainly in the colorectum, reducing off‐target effects in liver and spleen.^174^

**FIGURE 21:**
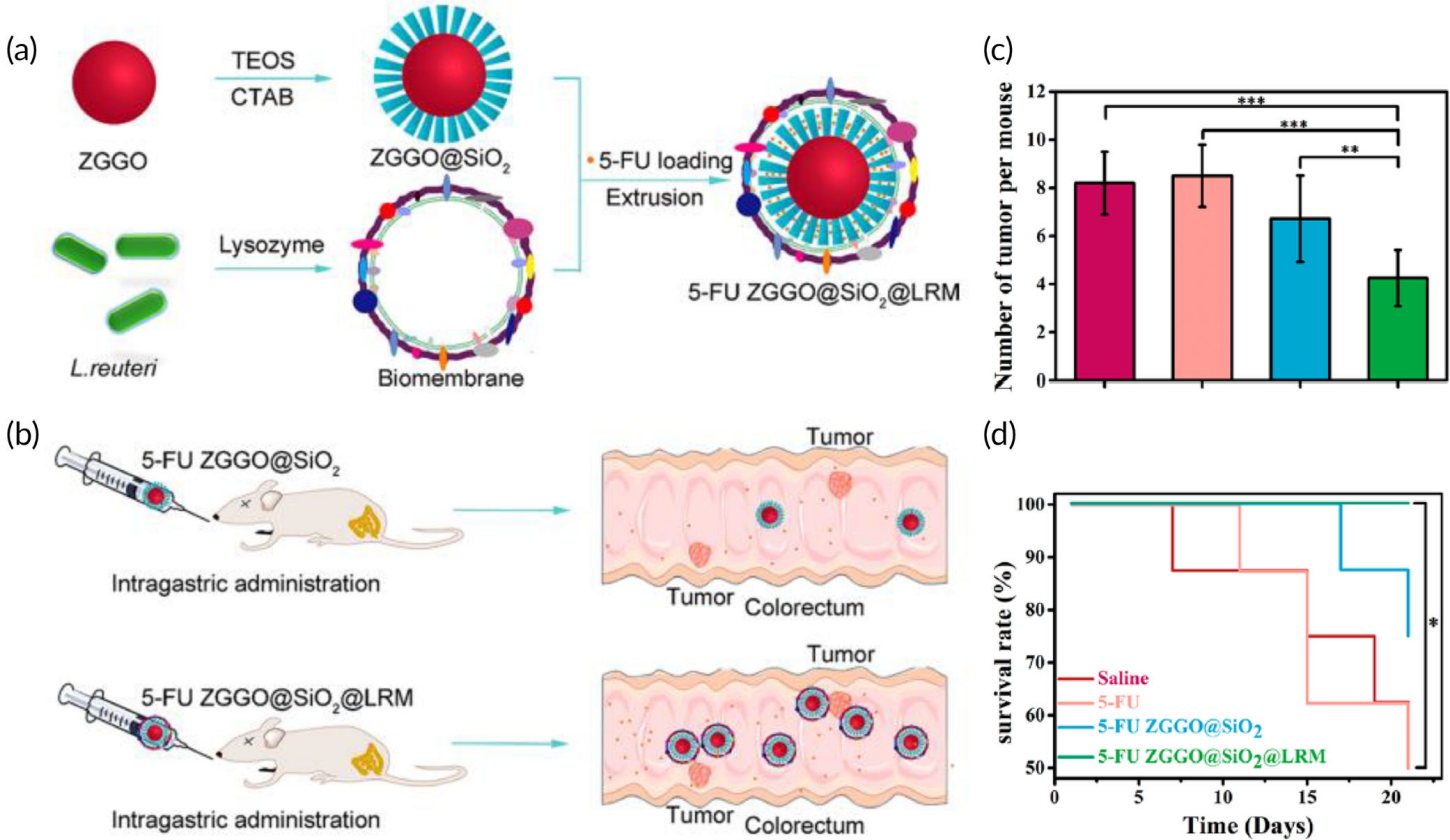
Bacterial‐bioinspired nanostructures for imaging and targeted chemotherapy of colorectal cancer. (a) Schematic depicting of the preparation of 5‐FU ZGGO@SiO_2_@LRM by coating 5‐FU ZGGO@SiO_2_ with *Lactobacillus reuteri* biomembranes. (b) Accumulation of 5‐FU ZGGO@SiO_2_@LRM in the colorectum tumor area after intragastric administration in tumor‐bearing mice. (c) Evaluation of the number of tumors per mouse in the colorectum in tumor‐bearing mice receiving different treatment (saline, 5‐FU, 5‐FU ZGGO@SiO_2_, and 5‐FU ZGGO@SiO_2_@LRM, respectively in that order from left to right). (d) Survival rate (%) of tumor‐bearing mice after intragastric administration of different treatments. Reproduced with permission.^175^ Copyright 2019, American Chemical Society. 5‐FU, 5‐fluorouracil; 5‐FU ZGGO@SiO_2_, ZGGO@SiO_2_ carrying anti‐cancer dug 5‐FU; 5‐FU ZGGO@SiO_2_@LRM, 5‐FU ZGGO@SiO_2_ coated with *Lactobacillus reuteri* biofilms; ZGGO@SiO_2_, persistent luminescence mesoporous silica nanoparticle.

Similarly, another bacterial biofilm‐coated, 5‐fluorouracil‐loaded biomimetic nanosystem was developed for colorectal cancer‐targeted chemotherapy. In this study, 5‐fluorouracil was loaded onto NIR persistent luminescence mesoporous silica NPs (5‐FU ZGGO@SiO_2_), which were then coated with a *Lactobacillus reuteri* (*L. reuteri*) biofilm (LRM), a Gram‐positive commensal bacterium of the human gastrointestinal tract, to yield a bacterial‐bioinspired nanostructure (5‐FU ZGGO@SiO_2_@LRM) (Figure 21a).^175^ The LRM coating offered protection against gastric acid digestion, avoided immune phagocytosis, improved the active‐targeting delivery to the colorectum, and enabled the sustained release of 5‐fluorouracil for more than 24 h following intragastric administration. The in vivo location of LRM‐coated NPs was investigated by in vivo luminescence imaging of ZGGO@SiO_2_. The nanosystem concentrated mainly in the colorectum, and has not entered into the bloodstream, thus avoiding damage to the liver and spleen (Figure 21b). In summary, LRM‐coated NPs showed promising antitumor effects by suppressing the growth of colorectal tumors and prolonging the survival time of tumor‐bearing mice in vivo (Figure 21c,d).^175^

**FIGURE 22:**
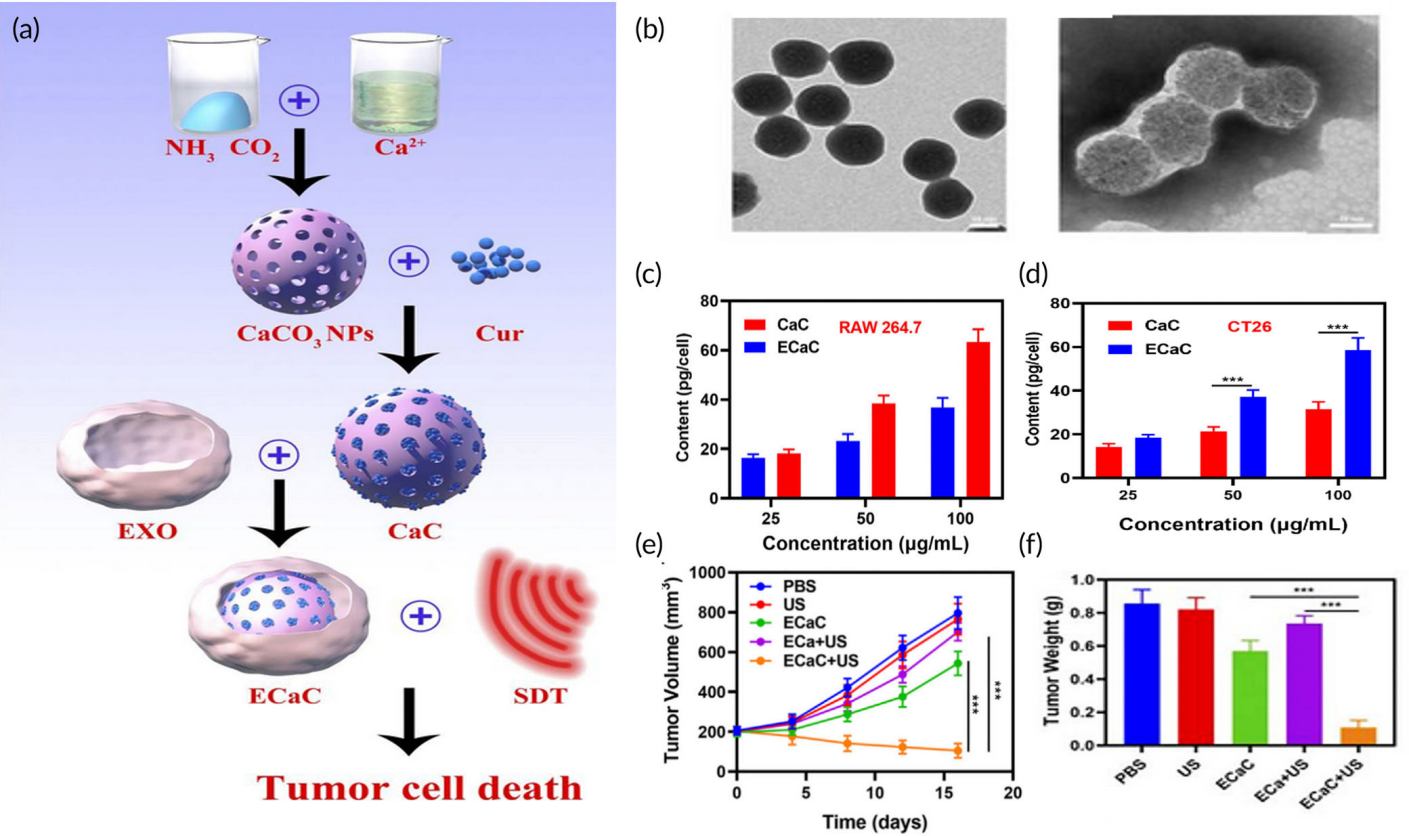
Colon cancer cell‐derived exosomal membrane‐coated, sonosensitizer‐loaded nanosystems for colorectal cancer‐targeted SDT upon US treatment. (a) Schematic depicting of the ECaC preparation by coating CaC with CT26 colon cancer cell‐derived exosomal membranes. (b) TEM images of (I) CaCO_3_ NPs (II) ECaC (in which a core‐shell nanostructure can be observed). (c) Uptake of CaC and ECaC (at different concentrations) by RAW 246.7 macrophage cells. (d) Uptake of CaC and ECaC (at different concentrations) by CT26 colon cancer cells. (e) Tumor volume (mm^3^) after different treatments. (f) Tumor weight (g) after different treatments. Reproduced with permission.^61^ Copyright 2022, Frontiers Media S.A. CaC, Cur‐loaded CaCO_3_ NP; CaCO_3_ NP, mesoporous calcium carbonate nanoparticle; Cur, curcumin; ECaC, EXO‐coated CaC; EXO, CT26 colon cancer cell‐derived exosomal membrane; SDT, sonodynamic therapy; TEM, transmission electron microscopy; US, ultrasound.

Naturally, cancer cell‐secreted exosomes also present themselves as promising biomembrane coatings for nanomaterials to provide highly specific, effective, and safer cancer interventions. In one study, CT26 colon cancer cell‐derived exosomal membranes were employed to coat mesoporous calcium carbonate (CaCO_3_) NPs loading curcumin (Cur, a sonosensitizer), named CaC, yielding a biomimetic exosome‐mimicking core‐shell nanosystem (ECaC) for colorectal cancer‐targeted SDT (Figure 22a,b).^61^ Compared to non‐coated CaC, ECaC showed enhanced immune escaping, prolonged duration of systemic circulation, and superior tumor‐targeting features, resulting in reduced uptake by RAW 246.7 macrophage cells (Figure 22c), and enhanced accumulation at homotypic CT26 tumors (Figure 22d). Once internalized by tumors, CaCO_3_ NPs were degraded in the acidic conditions of the tumor microenvironment, releasing both Cur for SDT and Ca^2+^ for destroying tumoral mitochondria, rendering tumor cells vulnerable. This sensitized tumor cells to Cur‐mediated SDT upon US treatment to produce synergistic antitumor effects. A superior tumor growth suppression efficacy was observed in the ECaC plus US treatment group (Figure 22e,f), indicating the great potential of exosome‐mimicking nanosystems for colorectal cancer therapy.^61^

### 7.2 Liver cancer

Exosomal membrane‐coated NPs have also been studied to improve the overall efficacy of anti‐HCC approaches.^176^ Molecular‐targeted therapy has brought new insights for cancer therapy. Sorafenib, a protein kinase inhibitor, is a molecular targeted drug indicated for advanced HCC therapy. However, concerns related to the lack of tumor‐targeting, the rapid macrophage‐mediated immune clearance in vivo, as well as the low drug concentration at tumors hinder its anticancer efficacy. To overcome these issues, one study harnessed the high biocompatibility, immune escaping, and intrinsic tumor‐homing features of huh‐7 liver cancer cell‐derived exosomes to encapsulate sorafenib‐loaded mPEG‐PLGA NPs aiming the targeted delivery of sorafenib to HCC.^176^ In vitro studies showed the enhanced tumor accumulation of exosome membrane‐coated NPs not only at Huh‐7 cells, but also at MHCC97H cells, which reveals their cross‐reactivity against different HCC cell lines. In the in vivo studies using H22 tumor‐bearing mice models, a superior antitumor efficacy was reported into the exosomal membrane‐coated NPs treatment group, in which a tumor volume reduction by 1.6 times was observed on day 14. In summary, the greater tumor penetration and accumulation mediated by the exosome membrane coating resulted in greater tumor enrichment with sorafenib for effective HCC therapy.^176^

## 8 CURRENT CHALLENGES TOWARD CLINICAL TRANSLATIONS: CRITICAL OBSTACLES

Translating cell membrane‐coated NPs from laboratory experiments to clinical settings poses several critical obstacles that are not only related to the challenging large‐scale/industrial scale production and lack of standardized and optimized preparation methods, but also to the safety and toxicological concerns of biomimetic NPs.^59^, ^68^

### 8.1 Large‐scale production and standardization of preparation techniques

Despite all the advancements in the field of utilizing cell membrane‐camouflaged nanocarriers for therapeutic purposes, there are still challenges concerning their clinical applications like the fact that they are limited to lab‐scale experiments, causing problems for industrial production, mainly centered on their low efficiency and inconsistent results, their intricate functionalization, the lack of standardized and optimized methods for membrane extraction and membrane coating, as well as sustaining the cell membrane's integrity and functionality during extraction process for effective membrane‐coated NPs fabrication. Moreover, careful considerations are needed to provide their long‐term storage stability and protect them against endotoxins, viruses, and pyrogens to prevent loss of therapeutic efficiency, since the biomimetic membranes hinder premature drug release and prolong circulation time in the bloodstream, facilitating the site of interest targeting.^177^, ^178^

The financial cost, operational complexity, and time efficiency are other challenges for effective clinical applications of these nano‐delivery systems which should be considered seriously despite their successful potential in laboratory settings with model animals. Taken together, the present limitations demand technical proficiency and high‐quality control to make the from bench to clinical practice, possible.^177^, ^178^

Additionally, it should be noted that the current technologies for the scaling up fabrication of cell membrane‐coated NPs still face several challenges.^59^, ^68^ In the first place, the production of cell membrane‐coated NPs relies on biological membranes derived from cells, such as red blood cells, cancer cells and immune cells, the scale‐up production of cell membrane‐coated NPs requires large quantities of various cells membranes and a continuous supply of cells or biological membranes, which can be challenging in cell culture and membrane purification. Besides, due to the heterogeneity of cells, the cell membranes extracted from various batches may exhibit batch‐to‐batch variability in protein composition, lipid structure, and integrity, leading to inconsistent NP behavior. On the second place, the scale‐up production of cell membrane‐coated NPs may impose higher requirements on fabrication techniques and control over manufacturing conditions. Currently, no industrial‐scale bioreactors are specifically designed for the fabrication of cell membrane‐coated NPs. Techniques such as extrusion, sonication, and microfluidics have been shown to work well at small scales, but they may not be as effective for large‐scale production. To address these problems, advanced bioreactors for large‐scale cell culture and automated purification systems can be used for the large‐scale purification of cell membranes. Meanwhile, more efficient methods should be developed to improve the uniformity of membrane coating on NPs.^59^, ^68^

### 8.2 Safety and toxicological considerations

In addition, it is crucial to perform a comprehensive evaluation of the biomimetic NPs' toxicological and safety profiles before utilizing them in vivo and in preclinical investigations. A comprehensive understating of the short‐ and long‐term safety of cell membrane‐coated NPs must be performed over different conditions, mainly in chronic and repeated administrations, since long‐term exposure raises important toxicological and immunological concerns. Thus, the cumulative and long‐term effects on organs' biodistribution, function, and toxicity, and unwanted systemic immune responses must be evaluated over time.^59^, ^68^

On the other side, the nano‐related toxicity must also be considered in the short‐ and long‐term, demanding a rigorous evaluation of the NPs' cumulative effects in terms of their immunological, oxidative stress and inflammatory potential, as well as the NPs‐induced toxicity (carcinogenicity, genotoxicity and teratogenicity).^59^, ^68^ Thus, the NP's size and shape assessments, particularly in those possessing the ability to generate excessive ROS, are critical as this could determine their biodistribution in vivo and toxicity. Research has shown that in the case of inorganic core NPs and micron‐sized CuO NPs would deliver safely despite their potency to cause DNA damage, whereas SiO2 NPs (particle size from 30–40 to 100–150 nm) significantly decrease cytotoxicity.^179^, ^180^ The type of the membrane coating could also perform a key role in nanocarriers' safety, since biocompatible red blood cell membranes resolve material toxicity on bare NPs (including carbon nanotubes and iron NPs), successfully, leading to prolonged survival time without immune reactions evoking and extending circulation around 5.8 times longer than bare NPs.^177^, ^180^

Surfactants, amphiphilic molecules used for NPs' surface decorating, are other factors that considerably influence the bioactivity and colloidal stability of NPs. Covering NPs with various surfactants contributes to NPs' stabilization via interfacial tension reduction through electrostatic repulsive interactions and studies demonstrated good colloidal stability, low toxicity, and better uptake in cancer cells following NPs' functionalization with surfactants such as amphoteric ionic sulfobetaine silanes.^181^

## 9 CONCLUSIONS AND FUTURE PROSPECTIVES

In this review, we introduced the recent progress of the biomembrane‐coated biomimetic NPs for the diagnosis and treatment of gastrointestinal cancers. Although the biomimetic NPs have been attracted as a promising next‐generation delivery system for the treatment and diagnosis of gastrointestinal cancers, it is still in its infancy in terms of clinical applications to enhance patient survival and improve quality of life. The main challenges hindering the clinical application of nanomedicines can be broadly divided into three main parts, including controlled and reproducible synthesis, large‐scale production, and evaluation and screening.^182^

Looking toward the future, future research will likely focus on the production of the biomimetic NPs with optimal physicochemical parameters. Determining the optimal physicochemical parameters of the biomimetic NPs is critical for their successful clinical application. Much research has focused on the individual physicochemical parameter that can influence effective immune escape, tumor penetration and accumulation, cellular targeting and internalization, as well as controlled drug release. However, systematic parallel screening of the numerous properties of NPs is still difficult, due to the difficulties in synthesizing identical NPs rapidly, precisely, and reproducibly.^12^, ^183^, ^184^, ^185^

Regarding coating NPs core with biomembrane, maintaining the integrity of the biomembrane is of paramount importance. Researchers are likely to invest more in exploring novel quality control techniques to ensure a uniform and desire coating of the NPs with cell‐derived proteins. The integrity of the cell membrane coating is a factor that cannot be overlooked as it affects the internalization mechanism of the biomimetic NPs.^186^ However, due to the complex three‐dimensional structures of NPs, quality control of the uniformly coating of NPs with the same density of the desired cell‐derived proteins remains a drawback that limits its clinical applications. Furthermore, it is still difficult to remove the undesirable proteins but retain the desired proteins from the mass of membrane proteins used for coating NPs. Some of these proteins can cause immune rejection and thus raise safety issues, while others can lead to evasion of immune surveillance and thus enhance the circulation time of the NPs in the body to strengthen their efficacy.^71^ As a result, new large‐scale production techniques are now needed to be developed to minimize batch‐to‐batch variability and to ensure performance and quality. Currently, most of cell membrane‐coated NPs have a low storage stability and short shelf life, which is far away to achieve commercialization.^187^ Anticipating technological advances in the large‐scale production and quality control of biomimetic NPs, we hope to see reduced batch‐to‐batch variability and improved storage stability and shelf life, ultimately pushing biomimetic NPs closer to commercialization.

Lastly, the complex and diverse pathological features of malignant tumors currently remain a major obstacle to the clinical use of NPs. Preclinical animal tumor models can not accurately simulate the tumor characteristics of clinical human cancers and the synergistic effects of the human immune system and multiple organs on tumor proliferation and metastasis, so the actual therapeutic effectiveness of anti‐tumor drugs often deviates significantly from that of the animal experimental results, which greatly hinders the clinical translation of nanomedicines.^188^ We envisage significant investment in developing more representative preclinical models that can accurately simulate the complexity of human cancers, such as advanced animal tumor models or recently emerged organ on a chip system. The aim is to create models that mirror the intricate interplay between the human immune system and multiple organs in tumor proliferation and metastasis, thus providing more reliable estimates of the therapeutic effectiveness. Although current in vivo and in vitro research has proven the safety of cell membrane‐coated NPs, there is still a lack of clinical data on the biocompatibility and efficacy of these NPs in treating gastrointestinal cancers. In the future, related clinical trials can be conducted to further evaluate the therapeutic efficacy of these materials.

In recent years, those questions have been paid more and more attentions and some studies have brought us some encouraging results. While the road toward clinical applications of biomimetic NPs poses challenges, the potential rewards are vast. As more attention is focused on these areas, and as our understanding deepens, we are already beginning to see promising results. With the continuous research efforts, we can expect further breakthroughs in the years to come. It can be expected that with the continuous study, the clinical use of the biomimetic NPs still has a bright prospect.

## AUTHOR CONTRIBUTIONS

**Joana Lopes:** Writing – original draft. **Daniela Lopes:** Writing – original draft. **Mahzad Motallebi:** Writing – original draft. **Mengguang Ye:** Writing – original draft. **Yuxiang Xue:** Writing – original draft. **Amélia C. F. Vieira:** Writing – original draft; writing – review and editing. **Sachin Kumar Singh:** Writing – review and editing. **Kamal Dua:** Writing – review and editing. **Francisco Veiga:** Writing – review and editing. **Gautam Sethi:** Writing – review and editing. **Ana Cláudia Paiva‐Santos:** Writing – review and editing; conceptualization; supervision. **Pooyan Makvandi:** Writing – review and editing.

## CONFLICT OF INTEREST STATEMENT

The authors declare no conflict of interest.

## Data Availability

Data sharing is not applicable to this article as no new data were created or analyzed in this study.
